# Metabolic Variations in Bamboo Shoot Boiled Liquid During *Pediococcus pentosaceus* B49 Fermentation

**DOI:** 10.3390/foods14152731

**Published:** 2025-08-05

**Authors:** Juqing Huang, Meng Sun, Xuefang Guan, Lingyue Zhong, Jie Li, Qi Wang, Shizhong Zhang

**Affiliations:** 1 Institute of Food Science and Technology, Fujian Academy of Agricultural Sciences, Fuzhou 350003, China; jq_huang@zju.edu.cn (J.H.); guan-619@163.com (X.G.); lingyue_zhong@163.com (L.Z.); lijie282008@126.com (J.L.); 2 Key Laboratory of Processing of Subtropical Characteristic Fruits, Vegetables and Edible Fungi, Ministry of Agriculture and Rural Affairs of China, Fuzhou 350003, China; 3 Fujian Key Laboratory of Agricultural Product (Food) Processing, Fuzhou 350003, China; 4 College of Food Science, Fujian Agriculture and Forestry University, Fuzhou 350002, China; yuta1010@163.com; 5 Zhongsha Biological Health Industry Research Institute, Xiamen 361026, China; 6 Institute of Animal Husbandry and Veterinary Medicine, Fujian Academy of Agricultural Sciences, Fuzhou 350003, China

**Keywords:** metabolites of bamboo shoot boiled liquid, fermentation, *Pediococcus pentosaceus* B49, phenylpropanoids, bitter peptides

## Abstract

Bamboo shoot boiled liquid (BSBL), a processing byproduct containing soluble proteins, peptides, amino acids, carbohydrates, and phenolics, is typically discarded, causing resource waste and environmental issues. This study analyzed metabolic changes in BSBL during *Pediococcus pentosaceus* B49 fermentation. The result of partial least squares discriminant analysis (PLS-DA) revealed significant metabolite profile differences across fermentation times (0 h, 24 h, 48 h, 72 h, 96 h). The most substantial alterations occurred within the first 24 h, followed by stabilization. Compared to unfermented BSBL, fermented samples exhibited significantly elevated signal intensities for 5,7-dimethoxyflavone, cinnamic acid, 3,4-dihydro-2H-1-benzopyran-2-one, 6,8-dimethyl-4-hydroxycoumarin, and 2-hydroxycinnamic acid (*p* < 0.05), showing upward trends over time. Conversely, (+)-gallocatechin intensity decreased gradually. Bitter peptides, such as alanylisoleucine, isoleucylisoleucine, leucylvaline, and phenylalanylisoleucine, in BSBL exhibited a significant reduction following fermentation with *P. pentosaceus* B49 (*p* < 0.05). KEGG enrichment indicated tyrosine metabolism (ko00350) and arginine/proline metabolism (ko00330) as the most impacted pathways. These findings elucidate metabolic regulation in BSBL fermentation, supporting development of functional fermented bamboo products.

## 1. Introduction

Bamboo shoots, the edible young culms of bamboo plants, are a traditional and nutritious vegetable widely consumed in Asia, particularly in China [1]. Rich in dietary fiber, vitamins (e.g., B1, B2, and C), minerals (e.g., potassium and calcium), and bioactive compounds (e.g., phenolics and flavonoids), bamboo shoots exhibit numerous health benefits, including antioxidant, anti-inflammatory, and hypolipidemic effects [2]. Reflecting its pivotal role in the global bamboo economy, China stands as the world’s largest producer, yielding approximately 1.03 million tons of bamboo shoots annually, with an associated industry value exceeding USD 21.7 million [3]. Despite their nutritional merits, fresh bamboo shoots are highly perishable, necessitating processing to ensure shelf stability and year-round availability [4]. A substantial portion of the harvest enters the industrial processing stream. Industry reports and studies indicate that approximately 60% of China’s bamboo shoots are processed commercially, primarily into forms such as canned (boiled), dried, marinated, fermented products, and medicinal extracts, while the remaining 40% are consumed fresh [5]. Among these processed forms, boiled bamboo shoots, predominantly packaged in cans for domestic and international markets, represent a major industrial product category. A critical environmental challenge arising from this large-scale processing, particularly the industrial boiling step essential for preservation, is the generation of vast volumes of nutrient-rich wastewater, known as bamboo shoot boiled liquid (BSBL). At the industrial scale, it is estimated that processing one ton of bamboo shoots generates approximately 2 tons of BSBL [6]. Extrapolating from China’s annual processed shoot volume (roughly 0.618 million tons, based on 60% of 1.03 million tons), this translates to an industrial-scale generation exceeding 1.2 million tons of BSBL annually. This represents a significant waste stream distinct from the comparatively negligible quantities produced sporadically at the domestic level. Currently, this nutrient-laden effluent is predominantly treated as wastewater or discarded directly, leading to substantial resource wastage of valuable compounds and posing considerable environmental burdens due to its organic load and potential eutrophication effects [5]. BSBL contains soluble proteins, peptides, free amino acids, carbohydrates, and phenolic compounds leached from bamboo shoots during boiling [6]. We assumed that BSBL possesses potential prebiotic and antioxidant properties, making it a promising substrate for microbial fermentation. However, current utilization of BSBL remains limited. Exploring microbial fermentation to valorize BSBL could enhance its functional properties and enable its application in functional foods or bioactive ingredient production.

*Pediococcus pentosaceus*, a widely recognized lactic acid bacteria (LAB), is frequently applied in food fermentation thanks to its strong acid production capability and excellent tolerance, which significantly improve the flavor and quality of fermented products [7,8]. However, systematic studies focusing on metabolic changes during the fermentation of bamboo shoots or their by-products (i.e., BSBL) by *P. pentosaceus* remain insufficient. Employing *P. pentosaceus* to ferment BSBL presents a promising strategy to valorize this byproduct. The process aligns with circular economy goals by converting waste into high-value ingredients for nutraceuticals or functional beverages. Untargeted metabolomics can elucidate dynamic metabolic changes during fermentation, providing insights into strain-specific pathways and optimizing fermentation conditions for targeted bioactivity enhancement.

*P. pentosaceus* B49 is a strain that was isolated from human colostrum [9]. Our previous study has demonstrated its ability to relieve constipation in mice [9]. However, the effects of this strain fermentation on BSBL have not been investigated. This study aimed to investigate the changes in metabolite profiles during BSBL fermentation by *P. pentosaceus* B49. We utilizes BSBL as the raw material, with *P. pentosaceus* B49 as the fermentation strain, employing untargeted metabolomics via ultrahigh performance liquid chromatography (UHPLC-MS/MS) to systematically assess metabolite changes at different fermentation stages (0 h, 12 h, 24 h, 48 h, 72 h). Through multivariate statistical analysis, significant metabolites are identified, and combined with KEGG pathway enrichment analysis, the key metabolic pathways and changes in active substances during fermentation are clarified. The findings will enhance the understanding of metabolic regulation mechanisms in this process, providing a theoretical foundation for developing functional bamboo shoot fermentation products, and offering new pathways for the advanced development and utilization of bamboo shoot resources.

## 2. Materials and Methods

### 2.1. Materials and Reagents

Fresh spring shoots of moso bamboo (*Phyllostachys edulis*) were purchased from local markets. LC-MS-grade methanol was purchased from Fisher Scientific UK Ltd. (Loughborough, UK). 2-Amino-3-(2-chloro-phenyl)-propionic acid was obtained from Shanghai Aladdin Biochemical Technology Co., Ltd. (Shanghai, China). LC-MS-grade acetonitrile was purchased from UK Fisher Scientific Ltd. (Loughborough, UK). Formic acid was obtained from TCI (Shanghai) Development Co., Ltd. (Shanghai, China). Ammonium formate was obtained from Sigma-Aldrich (Shanghai) Trading Co., Ltd.‌ (Shanghai, China). Ultrapure water was generated using a Milli-Q system (Millipore, Bedford, MA, USA).

The *P. pentosaceus* B49 strain was obtained from the human colostrum according to our previous procedure [9]. It was preserved at the China General Microbiological Culture Collection Center (CGMCC, No. 15957). The strain was cultured in MRS broth at 37 °C for 24 h before fermentation.

### 2.2. Preparation of BSBL Broth for Fermentation

Fresh spring shoots of moso bamboo (*Phyllostachys edulis*) were peeled and washed, then the edible portion was sliced into approximately 1 cm × 2 cm × 0.3 cm pieces. The slices were heated in a boiling water bath for 30 min. After cooling, the bamboo shoot boiled liquid (BSBL) was obtained through filtration. Subsequently, the liquid was further concentrated to approximately one-tenth of the fresh weight of the edible portion of bamboo shoots to prepare the BSBL broth, which facilitates enhanced microbial growth. Then, a 5% inoculum of activated *P. pentosaceus* B49 strain was aseptically added to the BSBL broth within a laminar flow hood. Fermentation broth samples were collected at 0, 24, 48, 72, and 96 h (defined as BS0H, BS24H, BS48H, BS72H and BS96H, respectively), centrifuged (3000 rpm, 4 °C, 15 min), and the supernatants were stored at −20 °C for subsequent untargeted metabolomic analysis.

### 2.3. Extraction of Metabolites

The metabolites were extracted following a previously described method [10]. Briefly, frozen samples were thawed at 4 °C, vortexed for 1 min to ensure homogeneity, and mixed thoroughly. A precise volume of each sample was transferred into a 2 mL centrifuge tube. A volume of 400 µL of methanol was added to the aliquot, followed by vortexing for 1 min to facilitate metabolite extraction. The mixture was centrifuged at 12,000 rpm and 4 °C for 10 min. The supernatant was carefully transferred to a new 2 mL tube, concentrated under reduced pressure (or nitrogen stream), and dried. The dried residue was reconstituted in 150 µL of 2-chloro-l-phenylalanine (4 ppm) solution (prepared in 80% methanol/water), vortexed to ensure complete dissolution, and filtered through a 0.22 μm membrane. The filtrate was transferred to an LC-MS vial for subsequent analysis.

### 2.4. UHPLC-MS/MS Detection

The analysis was performed according to established methods [11,12] using a Vanquish UHPLC system (Thermo Fisher Scientific, Waltham, MA, USA) coupled to a Q Exactive Focus mass spectrometer (Thermo Fisher Scientific, Waltham, MA, USA). Chromatographic separation was achieved on an ACQUITY UPLC HSS T3 column (2.1 × 100 mm, 1.8 μm; Waters, Milford, MA, USA) maintained at 40 °C with a flow rate of 0.3 mL/min and injection volume of 2 μL. For positive ion mode (ESI+), mobile phases consisted of 0.1% formic acid in water (A2) and 0.1% formic acid in acetonitrile (B2), while negative ion mode (ESI−) used 5 mM ammonium formate (A3) and acetonitrile (B3). Both modes employed identical gradient programs: 0–1 min at 10% B, 1–5 min linear increase to 98% B, 5–6.5 min hold at 98% B, 6.5–6.6 min rapid return to 10% B, followed by 6.6–8 min re-equilibration. Mass spectrometric detection utilized an ESI ion source operating in full MS-ddMS^2^ mode with alternating spray voltages of +3.50 kV (ESI+) and −2.50 kV (ESI−). Key parameters included: sheath gas at 40 arbitrary units (arb), auxiliary gas at 10 arb, capillary temperature at 325 °C, MS1 scan range m/z 115–1720 with a resolution 70,000 (full width at half maximum, FWHM), and data-dependent MS^2^ scans (top 3 precursors per cycle) at 17,500 resolution with 30 eV normalized collision energy and automatic dynamic exclusion.

### 2.5. Metabolite Identification and Quantitative Analysis

Raw data were initially converted to the mzXML format using MSConvert from the ProteoWizard software suite (v3.0.8789) [13]. Subsequent data processing was conducted with the R package XCMS (version 3.12.0), which included feature extraction, retention time adjustment, and alignment [14]. Key processing parameters included: ppm set to 15, peakwidth at c(5, 30), mzdiff at 0.01, and centWave as the detection method. Systematic variations were minimized through area normalization. Metabolite identification was achieved by matching accurate mass and MS/MS spectral data against several databases: HMDB (http://www.hmdb.ca, accessed on 20 April 2025), MassBank (https://massbank.eu/MassBank/), KEGG (https://www.genome.jp/kegg/, accessed on 20 April 2025), LipidMaps (http://www.lipidmaps.org, accessed on 20 April 2025), mzCloud (https://www.mzcloud.org, accessed on 20 April 2025), as well as a proprietary metabolite database from Panomix Biomedical Tech Co., Ltd. (Suzhou, China). The molecular weights of metabolites were determined by analyzing the m/z values of precursor ions in the MS data. Molecular formulas were inferred based on ppm tolerance and adduct ion types, followed by database matching. Additionally, MS/MS spectra from the quantitative data table were compared with the characteristic fragment ions and related information for each compound within the databases to confirm metabolite identities.

For multivariate statistical analysis, supervised partial least squares discriminant analysis (PLS-DA) was performed using the ropls package (version 1.22.0) in R (version 3.12.0) [15]. Differential metabolites (DMs) were defined according to criteria including statistical significance (*p* < 0.05), variable importance in projection (VIP > 1), and specified fold change (FC) thresholds.

### 2.6. Pathway Analysis

Differential metabolites (DMs) were analyzed for pathway involvement using MetaboAnalyst (Version R 4.0) [16], which integrates robust pathway enrichment and pathway topology analysis. The metabolites identified through metabolomics were subsequently mapped onto KEGG pathways to facilitate the biological interpretation of complex systemic functions. Visualization of these metabolites and their associated pathways was accomplished with the KEGG Mapper tool.

### 2.7. Statistical Analysis

All experiments were carried out with three independent biological replicates. Data visualization was accomplished using Origin (version 2019) and Microsoft PowerPoint (version 2019). Statistical evaluation of the experimental results was conducted through one-way ANOVA, and subsequent comparisons were made using Duncan’s multiple range test with IBM SPSS Statistics 22. A *p*-value of less than 0.05 was considered statistically significant.

## 3. Results and Discussion

### 3.1. Overall Differences in Metabolite Profiles

**Figure 1:**
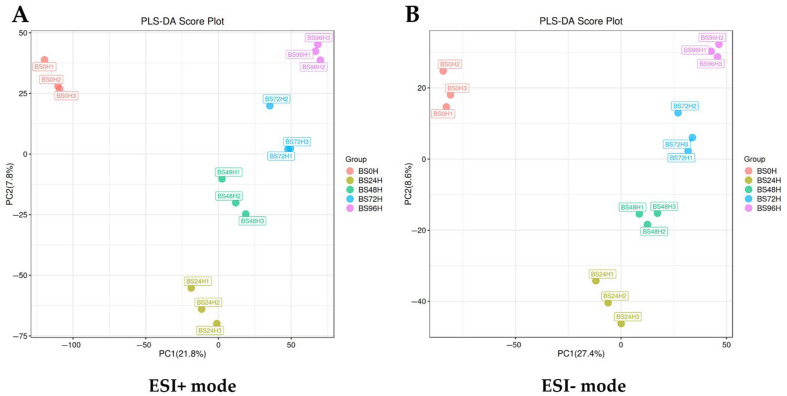
Partial least squares discriminant analysis (PLS-DA) of metabolites from BS0H, BS24H, BS48H, BS72H, and BS96H in both ESI+ (**A**) and ESI− (**B**) modes.

As a supervised learning method that emphasizes inter-group variation, PLS-DA was employed to analyze metabolite differentiation among BS0H, BS24H, BS48H, BS72H, and BS96H. PLS-DA analyses were conducted in both ESI+ and ESI− modes, and the results are presented in Figure 1. The results indicated a clear statistical separation among the metabolite profiles of BS0H, BS24H, BS48H, BS72H, and BS96H in both ESI+ and ESI− modes, demonstrating that the metabolite compositions of the fermented samples differed significantly at various time points.

### 3.2. Volcano Plot Analysis

**Figure 2:**
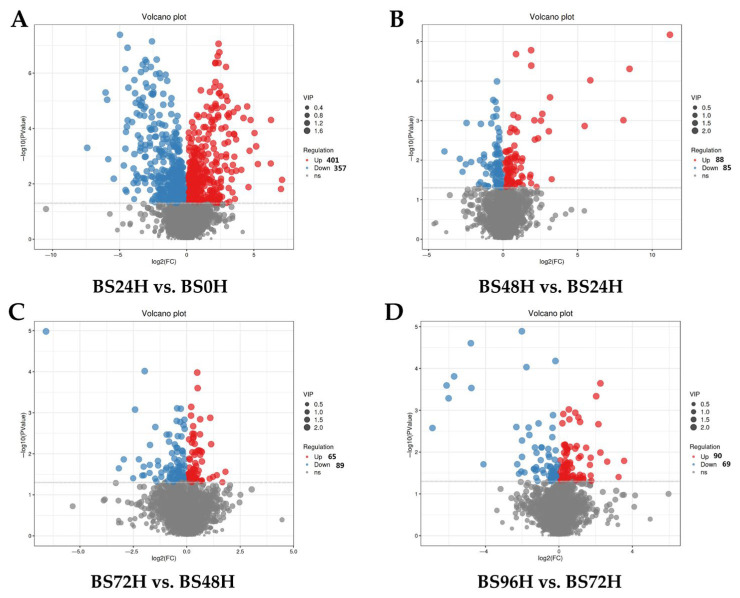
Volcano plots of identifed metabolites for the comparison of BS24H vs. BS0H (**A**), BS48H vs. BS24H (**B**), BS96H vs. BS48H (**C**), and BS96H vs. BS72H (**D**).

Volcano plot analyses were employed to visualize differential expression across sequential time comparisons: BS24H vs. BS0H, BS48H vs. BS24H, BS72H vs. BS48H, and BS96H vs. BS72H. These analytical outputs are systematically presented in Figure 2. The comparative analysis of BS0H vs. BS24H (Figure 2A) identified 401 significantly upregulated metabolites compared to 357 downregulated metabolites, highlighting substantial metabolic alterations in BSBL within the initial 24 h period. The BS48H vs. BS24H comparison (Figure 2B) demonstrated 88 upregulated metabolites versus 85 downregulated counterparts. Subsequent analysis of BS72H vs. BS48H (Figure 2C) revealed 65 upregulated metabolites compared to 89 downregulated compounds. The final comparison between BS96H and BS72H (Figure 2D) indicated 90 upregulated metabolites with 69 metabolites exhibiting significant downregulation (*p* < 0.05). The findings reveal that the most substantial metabolic alterations in BSBL occurred during the initial 24 h of fermentation, while subsequent changes between 24 and 96 h showed an overall stabilization pattern, indicating distinct metabolic phases during the fermentation process.

### 3.3. Identification of DMs

To further elucidate the metabolic variations in BSBL during the fermentation process of *P. pentosaceus* B49, we identified and listed the top 30 most significant and relevant differential metabolites (DMs) from the following comparisons: BS24H vs. BS0H, BS48H vs. BS24H, BS72H vs. BS48H, and BS96H vs. BS72H, with their corresponding log_2_ fold change (log_2_FC) values presented in Table 1 and Table 2.

As shown in Table 1, during the initial 24 h period, the primary upregulated DMs were 5,7-dimethoxyflavone; 1,3-nonanediol; (3Z,6Z)-3,6-nonadienal; medicocarpin; N-succinyl-2-amino-6-ketopimelate; 1-methoxy-2-hydroxyanthracene; 6-hydroxymelatonin; D-phenyllactic acid; N-succinyl-L,L-2,6-diaminopimelate; 10-hydroxydecanoic acid; (E)-3-heptenyl acetate; toluene-cis-dihydrodiol; citramalate; 2-inosose; N-acetyl-serylaspartic acid; ornithine; and β-alanine. Of particular note, 5,7-dimethoxyflavone, a naturally occurring flavonoid, has been reported to demonstrate various biological activities, including antioxidant, antidiabetic, anti-obesity, anti-inflammatory, and anticancer properties [17,18,19]. Medicocarpin has demonstrated strong binding ability with angiotensin-converting enzyme 2, a property closely associated with potential anti-COVID-19 activity [20]. The compound 6-hydroxymelatonin is the primary metabolite of melatonin, which is a major biologically active molecule secreted by the pineal gland [21]. It possesses a variety of functions, including free-radical scavenging and the induction of both protective and reparative cellular mechanisms [21]. D-phenyllactic acid, a metabolite produced by lactic acid bacteria, has been demonstrated to exhibit antimicrobial and immunomodulatory activities [22]. Notably, 1,3-nonanediol and (3Z,6Z)-3,6-nonadienal, and (E)-3-heptenyl acetate were identified as flavor substances generated during BSBL fermentation by *P. pentosaceus* B49 within the initial 24 h period. The DMs showing downregulation in the BS24H vs. BS0H comparison primarily comprised oligopeptides (Table 1). These oligopeptides included: Ile-Val-Gln, Ile-Val-Gly, Phe-Ile, Ala-Ile, Ile-Ile, Trp-Ile, Val-Val-Ala, Val-Ile-Thr, Thr-Leu, and Ile-Val-Val. These peptides may originate from two distinct pathways: microbial degradation of BSBL proteins by *P. pentosaceus* B49 metabolites, or alternatively, as native components inherent to the BSBL matrix prior to fermentation. The compound 3-phosphoglycerate serves as a central metabolic intermediate in both glycolysis and the Calvin cycle, while 6-phosphogluconic acid operates within the oxidative pentose phosphate pathway, arising through glucose-6-phosphate oxidation [23]. The metabolic depletion of these compounds likely reflects active carbohydrate metabolism by *P. pentosaceus* B49 during the initial 24 h fermentation phase.

Homodolicholide is reported to be a kind of steroidal plant-growth regulator [24]. The observed accumulation of homodolicholide in BSBL fermented with *P. pentosaceus* B49 between 24 and 48 h likely stems from the microbial bioconversion of particular bamboo shoot sterols [25]. Alisol C monoacetate is a kind of triterpenoid that is reported to have an anti-liver fibrosis effect [26]. Zhang et al. demonstrated the anti-fatigue effects of a triterpenoid-rich extract obtained from Chinese bamboo shavings, suggesting that these bioactive compounds are inherently present in bamboo plants [27]. In this study, a significant upregulation of alisol C monoacetate and farnesyl acetate was observed during the 24 to 48 h BSBL fermentation process mediated by the *P. pentosaceus* B49 strain. These metabolic alterations are likely attributable to the bacterial bioconversion of terpenes constituents naturally occurring in bamboo shoots via the enzymatic activities of the *P. pentosaceus* B49 strain. The content of 7-methylxanthosine was also significantly increased during the 24~48 h fermentation of BSBL by *P. pentosaceus* B49, which may be attributed to the microbial methylation of purine compounds present in bamboo shoots. Gentisyl alcohol, a compound exhibiting radical-scavenging activity [28], was observed to be upregulated during the 24 to 48 h BSBL fermentation process mediated by *P. pentosaceus* B49. Similarly, 6-hydroxymelatonin, a metabolite of melatonin that also displays antioxidant activity [29], was continuously upregulated throughout the same fermentation period mediated by *P. pentosaceus* B49. Three distinct lipid classes including phosphatidylglyceride (PG) (16:0/16:0) and PG (18:1/18:1) along with triglycerides (TG) (24:2) are supposed to be the lipid components present in bamboo shoots, with their content exhibiting significant changes during the 24 to 48 h BSBL fermentation process mediated through *P. pentosaceus* B49. Phaseollin and vanylglycol, both classified as phenylpropanoid compounds, demonstrate distinct biological activities. Phaseollin acts as an antifungal phytoalexin [30], while vanylglycol may exhibit an anti-inflammatory property [31]. Both compounds experienced marked increases in signal intensity from 24 to 48 h in B49-fermented BSBL. N-acetylanthranilate and spermine are likely metabolic derivatives of amino acids in bamboo shoots generated by the *P. pentosaceus* B49 strain, exhibiting significant signal attenuation during the 24–48 h fermentation phase.

By comparing the overall differences in log_2_FC values at various fermentation stages (BS24H vs. BS0H, BS48H vs. BS24H, BS72H vs. BS48H, BS96H vs. BS72H) as shown in Table 1 and Table 2, it becomes evident that the magnitude of changes in metabolite signal intensity in BSBL fermentation broth gradually decreases as the fermentation progresses. Among the detected metabolites, N-demethylvindolidine—an indole alkaloid which is produced by removing a methyl group from the nitrogen atom of the vindolidine molecule [32]—was identified for the first time in BSBL fermentation broth in this study, likely representing a demethylation product of vindolidine metabolized by the *P. pentosaceus* B49 strain [33]. 5-methylthioadenosine may be a metabolic product of S-adenosylmethionine processed by *P. pentosaceus* B49, and prior research has highlighted its potential anticancer properties [34]. Val-Ile-Val-Leu-Leu is likely an oligopeptide synthesized by the B49 strain using amino acids from BSBL. Purpuritenin B, a diterpenoid compound predominantly isolated from the seeds of *Annona purpurea* [35], was also detected in the BSBL fermentation broth. 13-oxo-octadecadienoic acid (13-OxoODE), an oxylipin derived from linoleic acid oxidation, plays a significant role in inflammation and immune regulation [36]. Although direct metabolism of 13-OxoODE by LAB has not been reported, some strains are known to modulate the transformation of unsaturated fatty acids like linoleic acid [37], which may indirectly affect oxylipin levels, including 13-OxoODE, under certain conditions. The signal intensity of alisol C monoacetate increased during the 24–48 h fermentation period but declined from 48 to 96 h, reflecting the dynamic transformation of triterpenoids in bamboo shoots by the *P. pentosaceus* B49 strain. Benzoylcholine, postulated as a flavor compound in fermented bamboo shoots, exhibited a significant reduction in signal intensity during the 48–72 h fermentation period by *P. pentosaceus* B49. The decrease in citramalate from 48 to 72 h may be associated with isoleucine synthesis via the threonine deaminase pathway, while the increase from 72 to 96 h could stem from the strain’s utilization of pyruvate and acetyl-CoA for biosynthesis. Manool, a diterpenoid abundant in sage, was detected in BSBL fermentation broth and has demonstrated selective antitumor activity against melanoma cells [38]. 3-hydroxy-3-methylglutaric acid, a derivative of glutaric acid, may be produced through the metabolism of carbohydrates and amino acids by the *P. pentosaceus* B49 strain, with its signal intensity rising between 72 and 96 h of fermentation. Neoxanthin, a carotenoid widely present in plants and algae [39], was detected in the BSBL fermentation broth, with increasing signal intensity between 72 and 96 h; previous studies have shown its effectiveness in relieving symptoms related to renal failure [40]. Neocembrene, a characteristic diterpene hydrocarbon of *R. communis* [41], showed increased signal intensity during the 72–96 h fermentation of BSBL by *P. pentosaceus* B49. Methylphosphatidylcholine is an essential component of cell membranes, contributing to the maintenance of structural stability, mediation of signal transduction, and regulation of membrane fluidity [42]. LAB can decompose or transform lipids present in bamboo shoots, leading to the synthesis or consumption of various phospholipids, including methylphosphatidylcholine. In this study, a significant reduction in the signal intensity of methylphosphatidylcholine (42:9) was observed in samples fermented with BSBL for 72 to 96 h. (13S)-13-hydroperoxy-9Z,11E-octadecadienoic acid, a lipoxygenase product of linoleic acid, exhibited decreased signal intensity from 72 to 96 h of fermentation. Naturally occurring carotenoids in bamboo shoots may be converted into derivatives such as 1-hydroxy-γ-carotene glucoside through microbial activity, and the decrease in its signal intensity between 72 and 96 h may be linked to β-glucosidase activity in the *P. pentosaceus* B49 strain. Notably, the signal intensity of 6-hydroxymelatonin continuously increased throughout the fermentation process, suggesting that the *P. pentosaceus* B49 strain may modulate the dynamic balance of melatonin and its metabolites via the tryptophan metabolic pathway.

Detailed information regarding metabolite identification is provided in the Supplementary Materials. The detailed information for the top 30 significant DMs identified in the comparisons of BS24H vs. BS0H, BS48H vs. BS24H, BS72H vs. BS48H, and BS96H vs. BS72H are presented in Table S1, Table S2, Table S3, and Table S4, respectively. The experimental fragment ions are shown in the identification charts for each DM, where the green line represents the DM and the red line represents the standard from the database.

### 3.4. Variations in Selected Phenylpropanoids Metabolites

**Figure 3:**
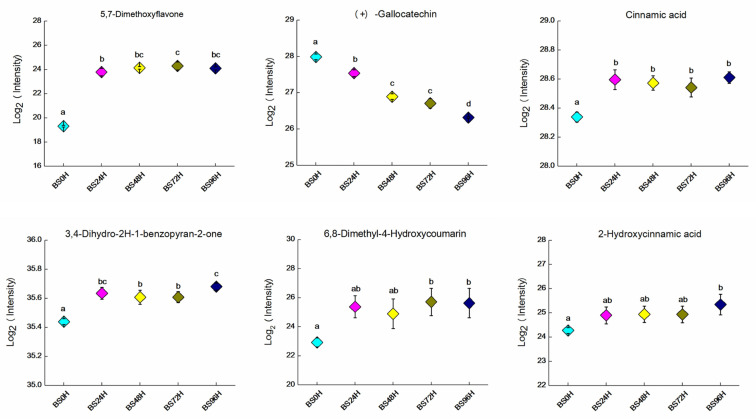
Dynamic variations in selected phenylpropanoid metabolites in BSBL during fermentation with *P. pentosaceus* B49. Data represent the mean ± SD (n = 3). (a–d), mean values with different letters over the symbols are significantly different (*p* < 0.05) according to a Duncan’s multiple range test.

LAB fermentation significantly modulates phenolic profiles and bioactivities in plant-based foods [43]. LAB hydrolyzes phenolic glycosides into bioactive aglycones, thereby enhancing free phenolic content and antioxidant capacity through enzymatic bioconversion [44,45,46]. For instance, Lu et al. reported that fermentation and enzymatic hydrolysis increased the total phenolic content of guava leaf tea and enhanced both antioxidant and α-glucosidase inhibitory activities [44]. Some studies have found that spontaneous fermentation increases the content of certain phenolic acids and flavonoids, thereby improving the antioxidant and anti-inflammatory activities of extracts derived from tomato, grape, and coffee residues [45]. Zhang et al. investigated the influence of *Bacillus subtilis* fermentation on the composition of phenolic compounds and antioxidant activity in cornmeal [46]. Their results indicated that the fermentation process significantly increased both total phenolic and total flavonoid contents. Therefore, LAB fermentation plays a crucial role in promoting the release of phenolic compounds and enhancing their bioactivities in plant-based food. Phenylpropanoids including flavonoids, coumarins, are important bioactive compounds, with numerous studies confirming their significant antioxidant, antidiabetic, and antiobesity potential [47]. Therefore, this study focused on examining the dynamic changes in selected phenylpropanoid and polyketide metabolites during the BSBL fermentation process, as illustrated in Figure 3.

As depicted in Figure 3, compared with the unfermented samples, the signal intensities of 5,7-dimethoxyflavone, cinnamic acid, 3,4-dihydro-2H-1-benzopyran-2-one, 6,8-dimethyl-4-hydroxycoumarin, and 2-hydroxycinnamic acid in the BSBL fermentation broth were significantly elevated (*p* < 0.05), and these metabolites generally exhibited an upward trend as fermentation time increased. Conversely, the signal intensity of (+)-gallocatechin decreased gradually during fermentation, which is likely associated with its metabolic utilization by the *P. pentosaceus* B49 strain.

### 3.5. Variations in Bitter Peptides

**Figure 4:**
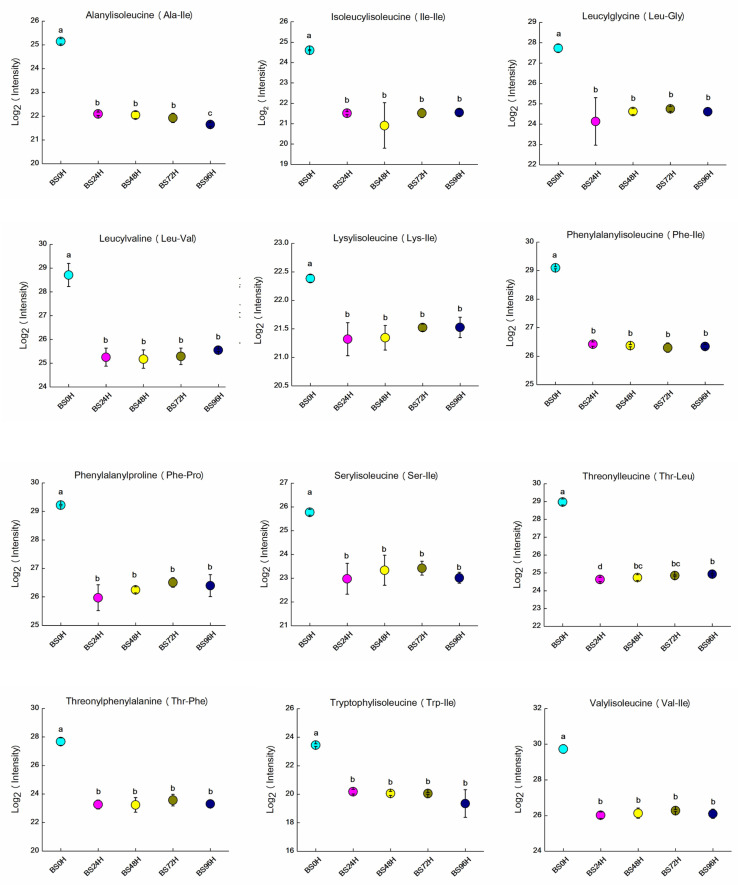
Temporal variations in bitter peptides in BSBL during fermentation with *P. pentosaceus* B49. Data represent the mean ± SD (n = 3). (a–d), mean values with different letters over the symbols are significantly different (*p* < 0.05) according to a Duncan’s multiple range test.

Preliminary experimental results indicate that B49 fermentation significantly reduces the bitterness of BSBL. Among the 20 common amino acids, those most commonly associated with bitterness are leucine, isoleucine, valine, phenylalanine, methionine, tryptophan, lysine, and threonine. Generally, peptides composed of these amino acids also exhibit a bitter taste [48]. In this study, twelve of the most bitter short peptides were identified from the BSBL fermentation sample, namely alanylisoleucine (Ala-Ile), isoleucylisoleucine (Ile-Ile), leucylglycine (Leu-Gly), leucylvaline (Leu-Val), lysylisoleucine (Lys-Ile), phenylalanylisoleucine (Phe-Ile), phenylalanylproline (Phe-Pro), serylisoleucine (Ser-Ile), threonylleucine (Thr-Leu), threonylphenylalanine (Thr-Phe), tryptophylisoleucine (Trp-Ile), and valylisoleucine (Val-Ile). Therefore, this research focused on analyzing the changes in bitter peptides in BSBL during *P. pentosaceus* B49 fermentation, with the relevant results presented in Figure 4. As shown in Figure 4, the signal intensities of bitter peptides in samples fermented for 24, 48, 72, and 96 h were significantly lower than those in the unfermented samples (all *p* < 0.05). Furthermore, there were no significant changes in these bitter peptides between 24 and 96 h of fermentation, indicating that the removal of bitter peptides by *P. pentosaceus* B49 primarily occurred within the first 24 h of fermentation.

### 3.6. Overall KEGG Pathway Enrichment Analysis

To elucidate the organism-specific metabolic dynamics during *P.* *pentosaceus* B49 fermentation of BSBL, KEGG pathway enrichment analysis was performed using the bacterial metabolic pathway database (*Pediococcus pentosaceus* KEGG organism code: ppe) as the primary reference framework, with supplemental mapping to plant pathways where relevant for bamboo-derived metabolites. This analysis specifically aimed to identify (1) metabolic pathways significantly altered by *P. pentosaceus* B49 activity across fermentation timepoints (0 h to 96 h), and (2) key bacterial biochemical transformations driving DM accumulation patterns. The DMs identified from pairwise comparisons of fermentation stages were annotated using KEGG Compound database and mapped to pathways via the KEGG Mapper search tool. Enrichment significance (Fisher’s exact test, *p* < 0.05) was calculated against the *P. pentosaceus* metabolic background.

**Figure 5:**
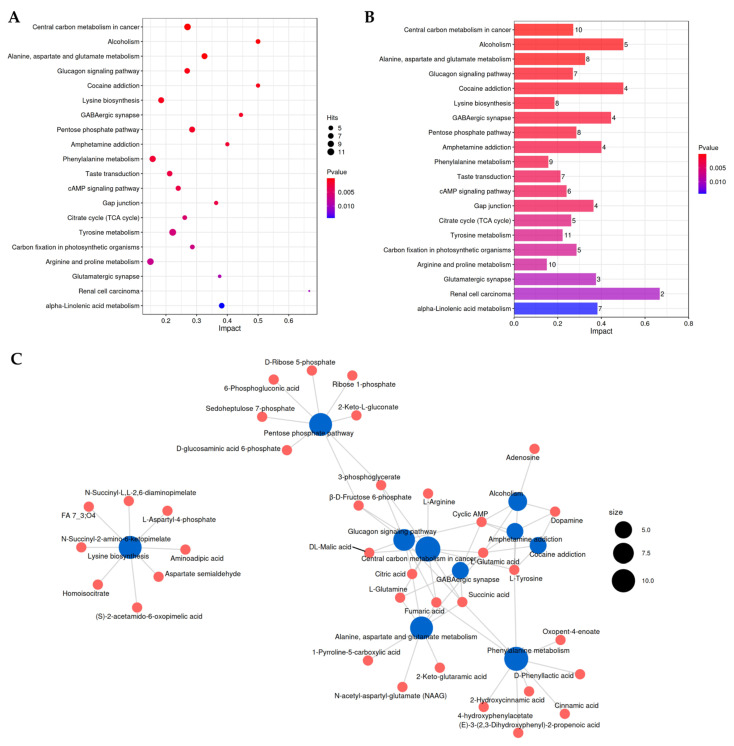
Kyoto Encyclopedia of Genes and Genomes (KEGG) pathway enrichment analysis of DMs from the comparison of BS0H vs. BS24H vs. BS48H vs. BS72H vs. BS96H; (**A**) Rich factor-based functional enrichment analysis; (**B**) Metabolite count annotation; (**C**) Metabolite interaction network analysis.

As shown in Figure 5A, the most significantly enriched pathways included core bacterial metabolic processes: alanine, aspartate and glutamate metabolism (ko00250); tyrosine metabolism (ko00350); arginine and proline metabolism (ko00330); pentose phosphate pathway (ko00030); citrate cycle (TCA cycle, ko00020); and lysine biosynthesis (ko00300). While some KEGG pathway designations reference human diseases (e.g., ko05230, ko05033), these represent evolutionarily conserved metabolic modules that function in bacterial systems. Figure 5B quantifies DM associations, with tyrosine metabolism (11 DMs), arginine/proline metabolism (10 DMs), and central carbon metabolism (10 DMs) showing highest involvement. Integration of DM profiles with enriched pathways revealed a bacterial-centric metabolic network (Figure 5C), highlighting the key pathways driving fermentation dynamics, including amino acid metabolism and energy production (pentose phosphate pathway, TCA cycle). This systematic approach clarifies how *P. pentosaceus* B49 reprograms both microbial and plant-derived metabolism during bamboo shoot fermentation.

## 4. Conclusions

This research explored metabolic shifts in BSBL during fermentation with *P.* *pentosaceus* B49. PLS-DA analyses in ESI+ and ESI− modes demonstrated significant variations in metabolite profiles at distinct time intervals (0 h, 24 h, 48 h, 72 h, 96 h). The most notable changes arose within the first 24 h, whereas subsequent modifications from 24 h to 96 h revealed a trend toward stabilization. Relative to unfermented controls, concentrations of compounds like 5,7-dimethoxyflavone, cinnamic acid, 3,4-dihydro-2H-1-benzopyran-2-one, 6,8-dimethyl-4-hydroxycoumarin, and 2-hydroxycinnamic acid were substantially higher (*p* < 0.05), with these metabolites generally rising as fermentation progressed. In contrast, (+)-gallocatechin levels steadily diminished. However, bitter peptides such as alanylisoleucine, isoleucylisoleucine, leucylglycine, leucylvaline, lysylisoleucine, and phenylalanylisoleucine registered significantly reduced abundances in fermented samples at 24 h, 48 h, 72 h, and 96 h compared to pre-fermentation states (all *p* < 0.05). KEGG pathway enrichment analysis identified tyrosine metabolism (ko00350) and arginine-proline metabolism (ko00330) as linked to the highest counts of DM species. These results deepen insights into metabolic control during BSBL fermentation, establishing a theoretical basis for functional bamboo shoot product development.

## Figures and Tables

**Table 1:** Fold change values of the top 30 significant differential metabolites (DMs) from sequential pairwise comparisons (BS24H vs. BS0H; BS48H vs. BS24H).

Name	log_2_FC (BS24H vs. BS0H)	Name	log_2_FC (BS48H vs. BS24H)
5,7-dimethoxyflavone	4.50	homodolicholide	11.19
1,3-nonanediol	3.79	alisol C monoacetate	5.47
(3Z,6Z)-3,6-nonadienal	2.93	7-methylxanthosine	3.15
medicocarpin	2.70	neoxanthin	2.54
N-succinyl-2-amino-6-ketopimelate	2.49	edetic acid	2.13
1-methoxy-2-hydroxyanthracene	2.46	norvaline	1.88
6-hydroxymelatonin	2.43	PG (16:0/16:0)	1.80
D-phenyllactic acid	2.39	gentisyl alcohol	1.30
N-succinyl-L,L-2,6-diaminopimelate	2.37	TG(24_2)	1.19
10-hydroxydecanoic acid	2.30	bruceine	1.00
(E)-3-heptenyl acetate	2.14	6-hydroxymelatonin	0.87
toluene-cis-dihydrodiol	2.14	suberylglycine	0.86
citramalate	2.12	2,3,5-hexanetrione	0.84
2-inosose	2.05	Pro-Met	0.82
N-acetyl-serylaspartic acid	1.89	farnesyl acetate	0.77
ornithine	1.73	1-octadecanethiol	0.75
β-alanine	1.49	phaseollin	0.70
Ile-Val-Gln	−2.03	5-methoxyindoleacetate	0.67
Ile-Val-Gly	−2.24	vanylglycol	0.47
DL-malic acid	−2.59	tartaric acid	−0.52
3-phosphoglycerate	−2.63	7-methylxanthine	−0.53
Phe-Ile	−2.68	(+)-gallocatechin	−0.65
Ala-Ile	−3.05	N-acetylanthranilate	−0.66
Ile-Ile	−3.09	acetyl-L-carnitine	−0.79
Trp-Ile	−3.26	spermine	−0.89
Val-Val-Ala	−3.35	phosphoribosyl pyrophosphate	−1.08
Val-Ile-Thr	−3.80	O-demethylmetoprolol	−1.30
Thr-Leu	−4.35	N-acetylprocainamide	−1.55
Ile-Val-Val	−4.41	leucocyanidin	−2.90
6-phosphogluconic acid	−4.98	PG (18:1/18:1)	−3.93

Note: Ile, Phe, Ala, Trp, Val, Thr, Pro, and Met represent isoleucine, phenylalanine, alanine, tryptophan, valine, threonine, proline, and methionine, respectively. PG: phosphatidylglycerol; TG: triglycerides.

**Table 2:** Fold change values of the top 30 significant differential metabolites (DMs) from sequential pairwise comparisons (BS72H vs. BS48H; BS96H vs. BS72H).

Name	log_2_FC (BS72H vs. BS48H)	Name	log_2_FC (BS96H vs. BS72H)
N-demethylvindolidine	1.10	citramalate	2.26
5-methylthioadenosine	0.71	Gly-Val	2.03
cyclic melatonin	0.71	manool	1.69
acetyl-L-carnitine	0.65	3-hydroxy-3-methylglutarate	1.48
Val-Ile-Val-Leu-Leu	0.63	neoxanthin	1.15
purpuritenin B	0.63	neocembrene	1.08
6-hydroxymelatonin	0.62	Gly-Pro-Arg	0.97
DG(15_0)	0.56	3-oxalomalic acid	0.89
canavaninosuccinate	0.51	ethyl tetradecanoate	0.61
Gly-Pro-Hyp	0.49	(S)-styrene oxide	0.60
sinuatol	0.44	suberoyl-L-carnitine	0.54
andrographic acid	0.41	Lys-Phe	0.46
3-phenylcatechol	0.40	5-methyltricosanoylcarnitine	0.46
obacunone	0.39	6-hydroxymelatonin	0.43
magnoflorine	0.34	N-hexanoyl-L-homoserine lactone	0.29
lysionotin	−0.52	dihydrovaltrate	0.28
9-methylhenicosanoylcarnitine	−0.58	myristoleate	0.28
9-cis-Retinoic acid	−0.69	7-methylxanthine	0.27
13-OxoODE	−0.72	Val-Arg-Ser	0.25
3-oxalomalic acid	−0.75	Thr-Arg-Glu	0.24
Ile-Gly-Lys	−0.95	Gly-Pro-Hyp	−0.36
palmitic acid	−0.98	matairesinoside	−0.47
γ-glutamyl-L-putrescine	−1.34	carviolin	−0.47
geranylhydroquinone	−1.35	andrographic acid	−0.56
Leu-Leu-Asp-Leu-Leu	−1.47	N-acetylanthranilate	−0.58
citramalate	−1.97	ethyl 3-indoleacetate	−0.64
peonidin 3-rhamnoside 5-glucoside	−2.24	DG (15:0/15:0)	−0.83
sorbose	−2.95	methylphosphatidylcholine (42:9)	−1.20
alisol C monoacetate	−3.18	13(S)-hydroperoxy-9,11-octadecadienoic acid	−1.77
benzoylcholine	−6.59	1-hydroxy-γ-carotene glucoside	−2.32

Note: Val, Ile, Leu, Gly, Pro, Hyp, Lys, Arg, Phe, Ser, and Thr represent valine, isoleucine, leucine, glycine, proline, hydroxyproline, lysine, arginine, phenylalanine, serine, and threonine, respectively. DG: dipentadecanoyl diacylglycerol.

## Data Availability

The original contributions presented in the study are included in the article/Supplementary Materials. Further inquiries can be directed to the corresponding authors.

## Supplementary Materials

The following supporting information can be downloaded at: https://www.mdpi.com/article/10.3390/foods14152731/s1, Table S1: Detailed information of the top 30 significant differential metabolites (DMs) from the comparison of BS24H vs. BS0H; Table S2: Detailed information of the top 30 significant differential metabolites (DMs) from the comparison of BS48H vs. BS24H; Table S3: Detailed information of the top 30 significant differential metabolites (DMs) from the comparison of BS72H vs. BS48H; Table S4: Detailed information of the top 30 significant differential metabolites (DMs) from the comparison of BS96H vs. BS72H.
