# Supplementary material for: Metabolic Variations in Bamboo Shoot Boiled Liquid During Pediococcus pentosaceus B49 Fermentation
Source: Foods. 2025 Aug 5;14(15):2731. doi: 10.3390/foods14152731 (PMC12346048; doi:10.3390/foods14152731)
Supplement: Supplementary file 1 [file foods-14-02731-s001.zip › foods-3766529-supplementary.pdf]

Table S1. Detailed information of the top 30 significant differential metabolites (DMs) from the comparison of BS24H vs. BS0H.

Name	Molecular formula	[M-H]-/[M-H]+ mass	Retention time (s)	mSigma values (ppm)	MSI_levels
5,7-dimethoxyflavone	$C_{17}H_{1}4O_{4}$	281.0892	103.3	7.363	MSI_level2
1,3-nonanediol	$C_{9}H_{20}O_{2}$	161.1463	231.2	11.78	MSI_level2
(3Z,6Z)-3,6-nonadienal	$C_{9}H_{14}O$	137.1045	189.4	3.31	MSI_level3
medicocarpin	$C_{22}H_{24}O_{9}$	431.1420	51.9	6.845	MSI_level1
N-succinyl-2-amino-6-ketopimelate	$C_{11}H_{15}NO_{8}$	290.0798	245.8	4.736	MSI_level3
1-methoxy-2-hydroxyanthracene	$C_{15}H_{12}O_{2}$	223.0837	53	14.015	MSI_level1
6-hydroxymelatonin	$C_{13}H_{16}N_{2}O_{3}$	249.1161	201.7	0.424	MSI_level1
D-phenyllactic acid	$C_{9}H_{10}O_{3}$	165.0630	205.7	4.935	MSI_level1
N-succinyl-L,L-2,6-diaminopimelate	$C_{11}H_{18}N_{2}O_{7}$	291.1114	245.8	12.39	MSI_level3
10-hydroxydecanoic acid	$C_{10}H_{20}O_{3}$	187.1412	219.5	4.599	MSI_level2
(E)-3-heptenyl acetate	$C_{9}H_{16}O_{2}$	157.1150	245.8	1.273	MSI_level2
toluene-cis-dihydrodiol	$C_{7}H_{10}O_{2}$	127.0681	54.4	13.938	MSI_level3
citramalate	$C_{5}H_{8}O_{5}$	147.0372	58	7.45	MSI_level2
2-inosose	$C_{6}H_{10}O_{6}$	177.0477	57.5	2.867	MSI_level1
N-acetyl-serylaspartic acid	$C_{9}H_{14}N_{2}O_{7}$	263.0801	225.2	8.131	MSI_level2
ornithine	$C_{5}H_{12}N_{2}O_{2}$	133.0899	44.7	3.378	MSI_level2
β-alanine	$C_{3}H_{7}NO_{2}$	88.0477	76.1	0.151	MSI_level1
Ile-Val-Gln	$C_{16}H_{30}N_{4}O_{5}$	359.2216	206.8	1.63	MSI_level3
Ile-Val-Gly	$C_{13}H_{25}N_{3}O_{4}$	288.1845	82.4	1.99	MSI_level3
DL-malic acid	$C_{4}H_{6}O_{5}$	133.0215	46.5	9.369	MSI_level1
3-phosphoglycerate	$C_{3}H_{7}O_{7}P$	184.9929	47.4	0.19	MSI_level1
Phe-Ile	$C_{15}H_{22}N_{2}O_{3}$	279.1630	210.4	3.262	MSI_level2
Ala-Ile	$C_{9}H_{18}N_{2}O_{3}$	201.1317	75	3.278	MSI_level2

Ile-Ile	$C_{12}H_{24}N_2O_3$	243.1787	164.8	1.262	MSI_level2
Trp-Ile	$C_{17}H_{23}N_3O_3$	316.1739	205	0.178	MSI_level2
Val-Val-Ala	$C_{13}H_{25}N_3O_4$	288.1845	131.7	1.99	MSI_level3
Val-Ile-Thr	$C_{15}H_{29}N_3O_5$	332.2107	192.8	0.261	MSI_level3
Thr-Leu	$C_{10}H_{20}N_2O_4$	233.1423	102.5	2.043	MSI_level2
Ile-Val-Val	$C_{16}H_{31}N_3O_4$	330.2314	203.7	0.239	MSI_level3
6-phosphogluconic acid	$C_6H_{13}O_{10}P$	275.0246	45.2	12.521	MSI_level1

Note: The Metabolomics Standards Initiative (MSI) categorizes metabolite identification into four hierarchical levels. Level 1 comprises identified compounds verified with standards under at least two orthogonal conditions; for GC-MS, this involves matching retention times or retention indices and mass spectra with standards, while LC-MS demands consistency in retention time, isotope distribution, and tandem mass spectra. Level 2 includes putatively annotated compounds lacking standard verification but showing mass spectra consistent with reference spectra from literature or databases. Level 3 consists of putatively characterized compound classes without standard verification, where mass spectra resemble those of known compound classes. Finally, Level 4 encompasses unknown compounds, representing metabolites that remain unidentified.

le S2. Detailed information of the top 30 significant differential metabolites (DMs) from the comparison of BS48H vs. BS24H.

Name	Molecular formula	[M-H]-/[M-H]+ mass	Retention time (s)	mSigma values (ppm)	MSI_levels
homodolicholide	$C_{29}H_{48}O_6$	491.34507	372.3	7.12	MSI_level2
alisol C monoacetate	$C_{32}H_{48}O_6$	527.34507	380.3	6.247	MSI_level2
7-methylxanthosine	$C_7H_{12}O_3$	145.0786	179.2	2.23	MSI_level3
neoxanthin	$C_9H_{11}NO_3$	180.07389	300	2.182	MSI_level1
edetic acid	$C_{11}H_15N_4O_6^+$	298.09915	53.3	12.326	MSI_level1
norvaline	$C_{40}H_{56}O_4$	599.4179	382.7	7.34	MSI_level3
PG(16_0_16_0)	$C_{10}H_{16}N_2O_8$	293.0907	324.5	0.759	MSI_level3
gentisyl alcohol	$C_5H_{11}NO_2$	118.079	196.6	6.575	MSI_level3
TG(24_2)	$C_{38}H_{75}O_{10}P$	723.50976	385.6	0.546	MSI_level2

bruceine	$C_7H_8O_3$	139.0473418	371.6	14.049	MSI_level3
6-hydroxymelatonin	$C_{27}H_{46}O_6$	467.32942	334.3	1.336	MSI_level2
suberylglycine	$C_{20}H_{26}O_9$	409.15767	293.8	5.137	MSI_level2
2,3,5-hexanetrione	$C_{13}H_{16}N_2O_3$	249.11609	201.7	0.424	MSI_level1
Pro-Met	$C_{10}H_{17}NO_5$	232.11067	250.4	0.604	MSI_level2
farnesyl acetate	$C_6H_8O_3$	129.04734	33.7	4.016	MSI_level2
1-octadecanethiol	$C_{18}H_{38}S$	287.26941	313.7	11.936	MSI_level2
phaseollin	$C_{20}H_{18}O_4$	323.1205	239.7	3.835	MSI_level2
5-methoxyindoleacetate	$C_{11}H_{11}NO_3$	206.0738896	202.6	0.005	MSI_level3
vanylglycol	$C_9H_{12}O_4$	185.0735552	321.7	2.474	MSI_level3
tartaric acid	$C_4H_6O_6$	149.01644	380.1	3.766	MSI_level1
7-methylxanthine	$C_6H_6N_4O_2$	165.04907	380.3	8.466	MSI_level2
(+)-gallocatechin	$C_{15}H_{14}O_7$	307.074	53.9	8.542	MSI_level3
N-acetylanthranilate	$C_9H_9NO_3$	178.05824	141.9	3.729	MSI_level1
acetyl-L-carnitine	$C_9H_{17}NO_4$	204.11575	83.9	1.12	MSI_level2
spermine	$C_{10}H_{26}N_4$	203.21574	44.7	0.059	MSI_level2
phosphoribosyl pyrophosphate	$C_5H_{13}O_{14}P_3$	390.95182	47	8.173	MSI_level2
O-demethylmetoprolol	$C_{14}H_{23}NO_3$	254.16778	76.2	6.9	MSI_level2
N-acetylprocainamide	$C_{15}H_{23}N_3O_2$	278.17902	211.4	12.341	MSI_level2
leucocyanidin	$C_{15}H_{14}O_7$	307.07395	54.4	4.149	MSI_level1
PG(18_1-18_1)	$C_{42}H_{79}O_{10}P$	773.54106	386.8	10.756	MSI_level2

Table S3. Detailed information of the top 30 significant differential metabolites (DMs) from the comparison of BS72H vs. BS48H.

Name	Molecular formula	[M-H]-/[M-H]+ mass	Retention time (s)	mSigma values (ppm)	MSI_levels
N-demethylvindolidine	$C_{21}H_{26}N_2O_3$	355.1943	197.7	4.413	MSI_level1

5-methylthioadenosine	$C_{11}H_{15}N_5O_3S$	298.0896	129.1	13.72	MSI_level1
cyclic melatonin	$C_{13}H_{14}N_2O_2$	229.1055	194.5	0.492	MSI_level3
acetyl-L-carnitine	$C_9H_{17}NO_4$	204.1158	83.9	0.192	MSI_level2
Val-Ile-Val-Leu-Leu	$C_{30}H_{57}N_5O_6$	584.4309	449.2	9.813	MSI_level2
purpuritenin B	$C_{19}H_{16}O_3$	293.1099	92.5	1.156	MSI_level2
6-hydroxymelatonin	$C_{13}H_{16}N_2O_3$	249.1161	201.7	11.327	MSI_level1
DG(15_0)	$C_{18}H_{34}O_5$	331.2406	315.4	12.631	MSI_level2
canavaninosuccinate	$C_9H_{16}N_4O_7$	291.1019	118.3	3.562	MSI_level3
Gly-Pro-Hyp	$C_{12}H_{19}N_3O_5$	286.1325	142	5.6	MSI_level2
sinuatol	$C_{21}H_{32}O_{13}$	491.1843	196	6.001	MSI_level3
andrographic acid	$C_{20}H_{28}O_6$	365.1886	311.5	1.556	MSI_level2
3-phenylcatechol	$C_{12}H_{10}O_2$	187.0681	43.1	9.789	MSI_level2
obacunone	$C_{26}H_{30}O_7$	453.1991	329.6	2.274	MSI_level2
magnoflorine	$C_{20}H_{24}NO_4^+$	343.1705	236.3	9.243	MSI_level2
lysionotin	$C_{18}H_{16}O_7$	343.0896	302.1	5.326	MSI_level2
9-methylhenicosanoylcarnitine	$C_{29}H_{57}NO_4$	484.4287	338.4	8.284	MSI_level2
9-cis-Retinoic acid	$C_{20}H_{28}O_2$	301.2089	236.3	1.517	MSI_level2
13-OxoODE	$C_{18}H_{30}O_3$	295.2195	370.1	7.45	MSI_level1
3-oxalomalic acid	$C_6H_6O_8$	207.0063	186.4	7.025	MSI_level2
Ile-Gly-Lys	$C_{14}H_{28}N_4O_4$	317.2110	56.6	11.503	MSI_level3
palmitic acid	$C_{16}H_{32}O_2$	255.2402	383.2	6.247	MSI_level1
γ-glutamyl-L-putrescine	$C_9H_{19}N_3O_3$	216.1426	327.2	14.144	MSI_level2
geranylhydroquinone	$C_{16}H_{22}O_2$	247.1620	382.8	4.413	MSI_level3
Leu-Leu-Asp-Leu-Leu	$C_{32}H_{59}N_5O_8$	642.4363	413.9	13.72	MSI_level2
citramalate	$C_5H_8O_5$	147.0372	58	0.492	MSI_level2
peonidin 3-rhamnoside	$C_{28}H_{33}O_{15}^+$	610.1819	402.8	0.192	MSI_level2

5-glucoside					
aorbose	$C_6H_{12}O_6$	179.0634	372.8	9.813	MSI_level2
alisol C	$C_{32}H_{48}O_6$	527.3451	380.3	1.156	MSI_level2
benzoylcholine	$[C_{12}H_{18}NO_2]^+$	207.1338	63.6	11.327	MSI_level2

Table S4. Detailed information of the top 30 significant differential metabolites (DMs) from the comparison of BS96H vs. BS48H.

Name	Molecular formula	[M-H]-/[M-H]+ mass	Retention time (s)	mSigma values (ppm)	MSI_levels
citramalate	$C_5H_8O_5$	147.0372	58	7.45	MSI_level2
Gly-Val	$C_7H_{14}N_2O_3$	173.1004	54.3	4.974	MSI_level1
manool	$C_{20}H_{34}O$	291.2610	381	0.249	MSI_level2
3-hydroxy-3-methylglutarate	$C_6H_{10}O_5$	163.0528	226.1	9.731	MSI_level3
neoxanthin	$C_{40}H_56O_4$	599.4179	382.7	7.34	MSI_level3
neocembrene	$C_{20}H_{32}$	273.2504	359	13.993	MSI_level2
Gly-Pro-Arg	$C_{13}H_{24}N_6O_4$	329.1859	152.5	10.455	MSI_level2
3-oxalomalic acid	$C_6H_6O_8$	207.0063	186.4	9.789	MSI_level2
ethyl tetradecanoate	$C_{16}H_{32}O_2$	255.2402	416.6	8.852	MSI_level2
(S)-styrene oxide	C_8H_8O	119.0575	73.6	10.377	MSI_level2
suberoyl-L-carnitine	$C_{15}H_{27}NO_6$	318.1838	238.2	14.254	MSI_level2
Lys-Phe	$C_{15}H_{23}N_3O_3$	294.1739	191.5	2.751	MSI_level2
5-methyltricosanoylcarnitine	$C_{31}H_{61}NO_4$	512.4600	354	6.071	MSI_level2
6-hydroxymelatonin	$C_{13}H_{16}N_2O_3$	249.1161	201.7	0.424	MSI_level1
N-hexanoyl-L-Homoserine lactone	$C_{10}H_{17}NO_3$	200.1208	161.4	6.564	MSI_level2

dihydrovaltrate	$C_{22}H_{32}O_8$	425.2097	395.2	6.311	MSI_level2
myristoleate	$C_{14}H_{26}O_2$	225.1933	247.5	1.574	MSI_level2
7-methylxanthine	$C_6H_6N_4O_2$	165.0491	380.3	8.466	MSI_level2
Val-Arg-Ser	$C_{14}H_{28}N_6O_5$	361.2121	379.7	5.2	MSI_level2
Thr-Arg-Glu	$C_{15}H_{28}N_6O_7$	405.2019	379.7	6.134	MSI_level3
Gly-Pro-Hyp	$C_{12}H_{19}N_3O_5$	286.1325	142	0.492	MSI_level2
matairesinoside	$C_{26}H_{32}O_{11}$	519.1945	272.2	3.321	MSI_level2
carviolin	$C_{16}H_{12}O_6$	301.0634	227.1	2.863	MSI_level2
andrographic acid	$C_{20}H_{28}O_6$	365.1886	311.5	9.813	MSI_level2
N-acetylanthranilate	$C_9H_9NO_3$	178.0582	141.9	3.729	MSI_level1
ethyl 3-indoleacetate	$C_{12}H_{13}NO_2$	204.0946	86.2	1.579	MSI_level2
DG(15_0)	$C_{18}H_{34}O_5$	331.2406	315.4	4.413	MSI_level2
methylphosphatidylcholine (42:9)	$C_{51}H_{84}NO_8P$	870.5934	399.6	10.917	MSI_level2
13(S)-Hydroperoxy-9,11-octadeca dienoic acid	$C_{18}H_{30}O_4$	311.2144	347.3	7.383	MSI_level1
1-hydroxy-γ-carotene glucoside	$C_{46}H_{68}O_6$	715.5016	423.1	4.271	MSI_level2

Experimental fragments of the top 30 significant differential metabolites (DMs) from the comparison of BS24H vs. BS0H.

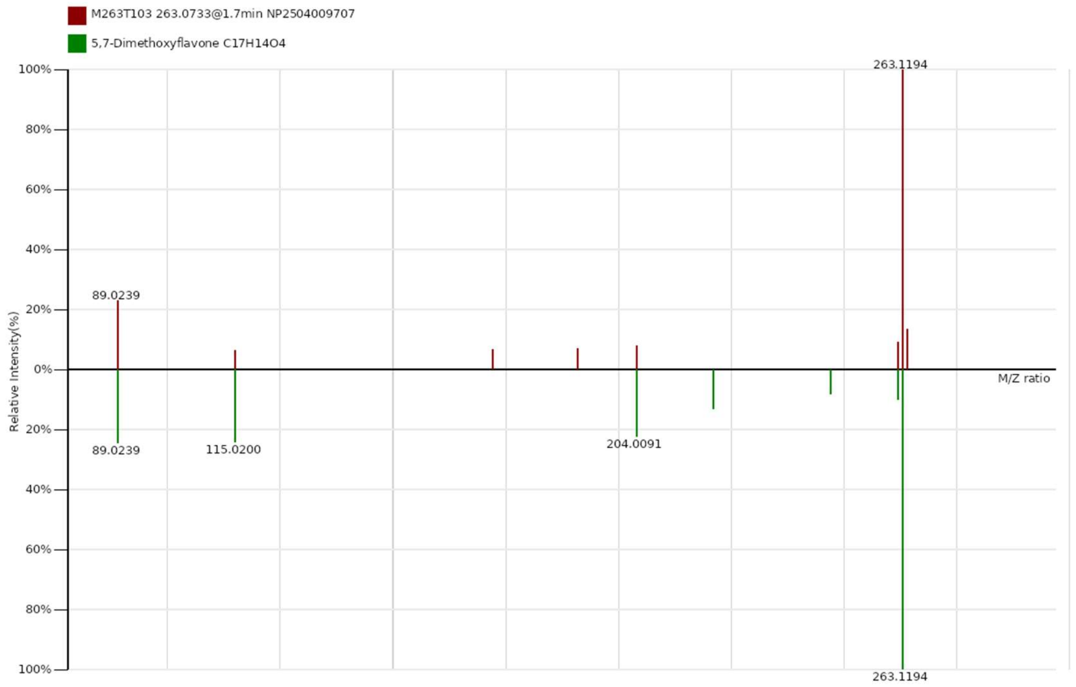

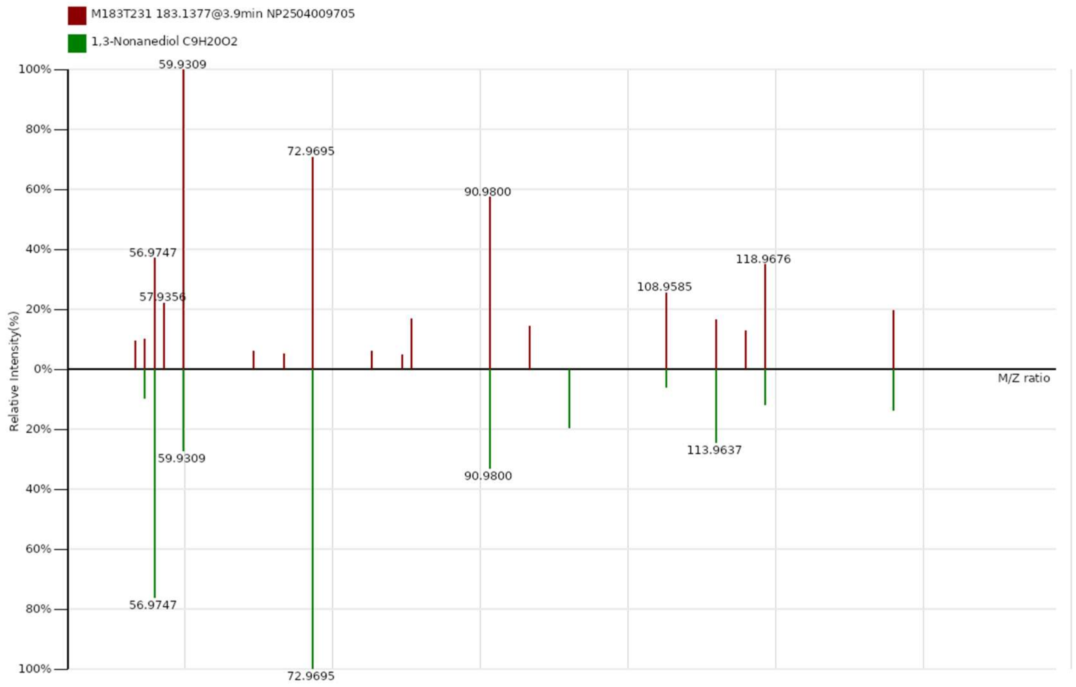

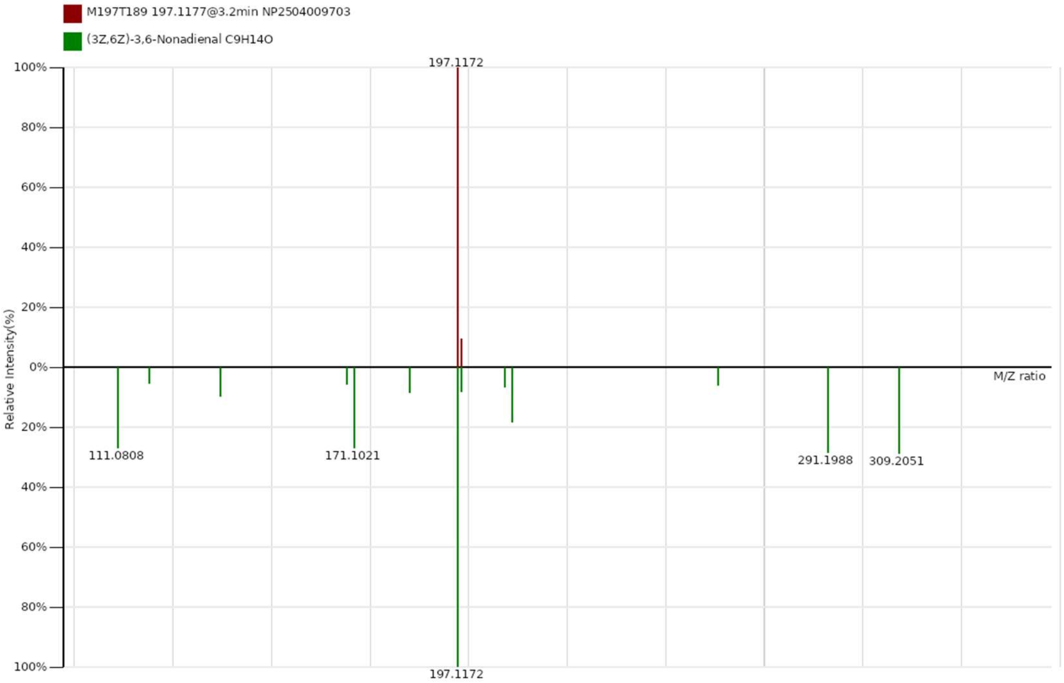

M197T189 197.1177@3.2min NP2504009703
(3Z,6Z)-3,6-Nonadienal C9H14O
Relative Intensity(%)
M/Z ratio
197.1172
111.0808
171.1021
291.1988
309.2051
197.1172

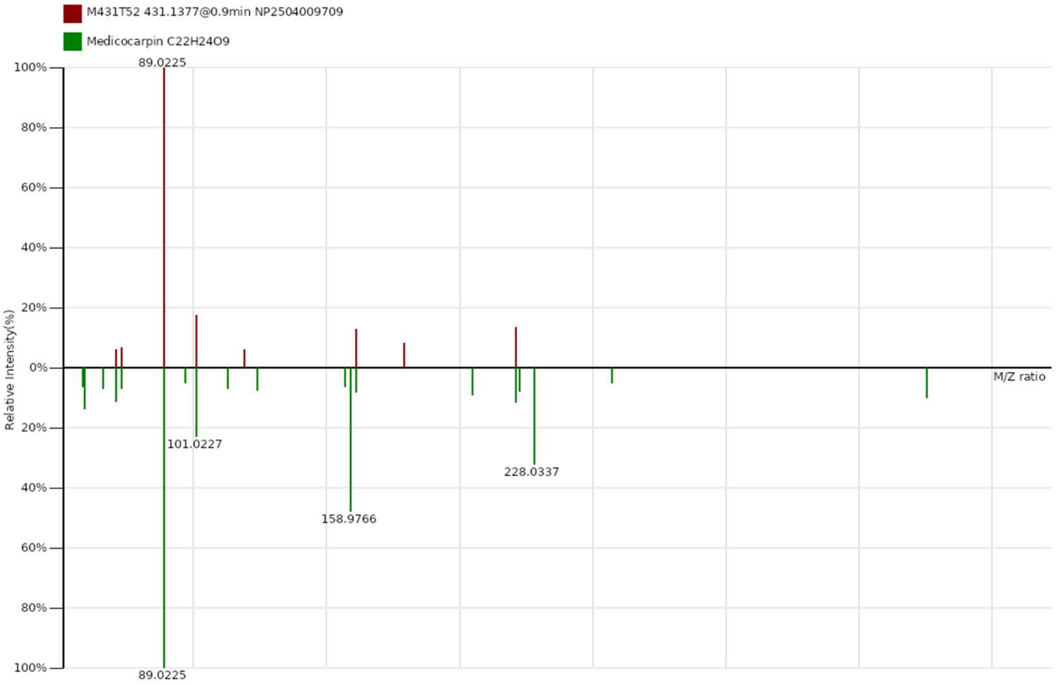

M431T52 431.1377@0.9min NP2504009709
Medicocarpin C22H24O9
Relative Intensity(%)
M/Z ratio
89.0225
101.0227
158.9766
228.0337
89.0225

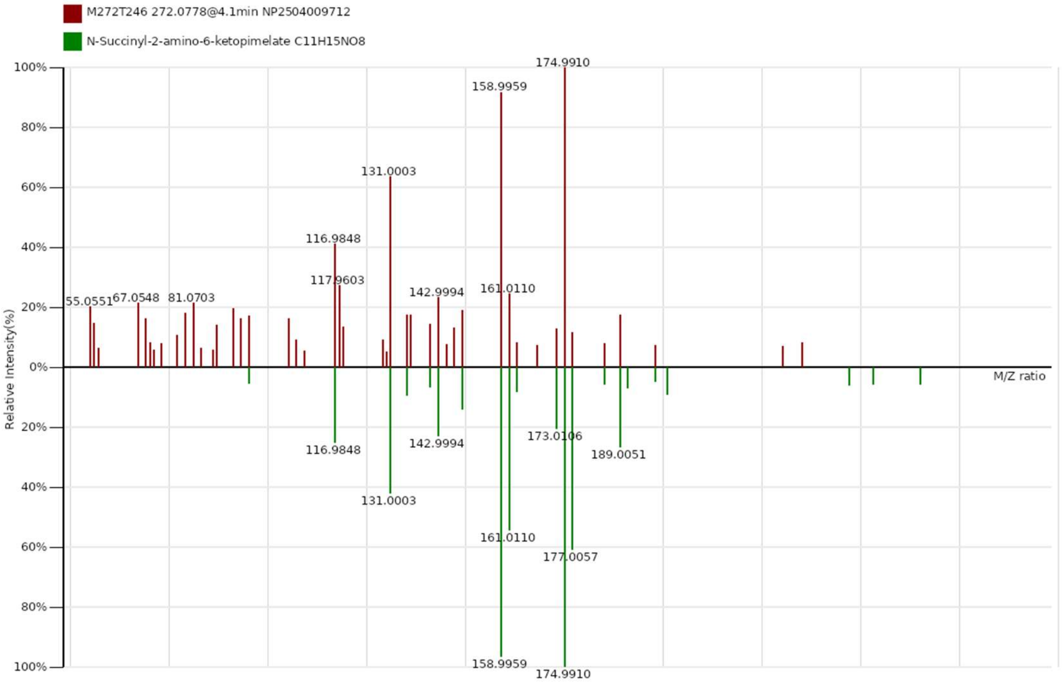
M272T246 272.0778@4.1min NP2504009712
N-Succinyl-2-amino-6-ketopimelate C11H15NO8
Relative Intensity(%)
M/Z ratio
174.9910
158.9959
131.0003
116.9848
117.9603
142.9994
161.0110
55.0551
67.0548
81.0703
173.0106
189.0051
177.0057

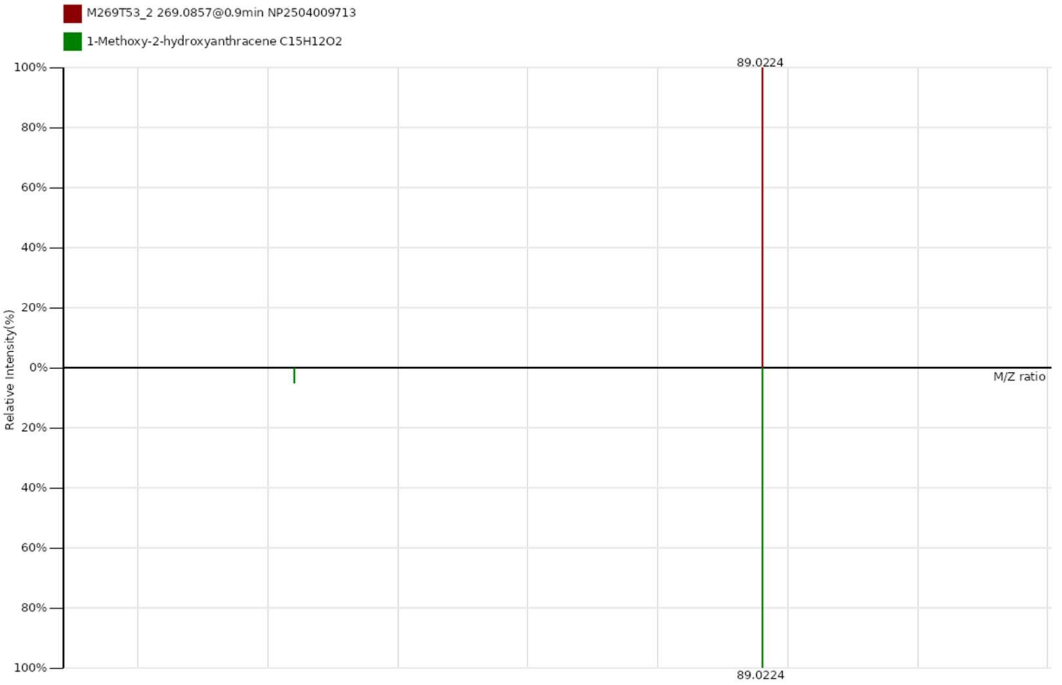
M269T53_2 269.0857@0.9min NP2504009713
1-Methoxy-2-hydroxyanthracene C15H12O2
Relative Intensity(%)
M/Z ratio
89.0224

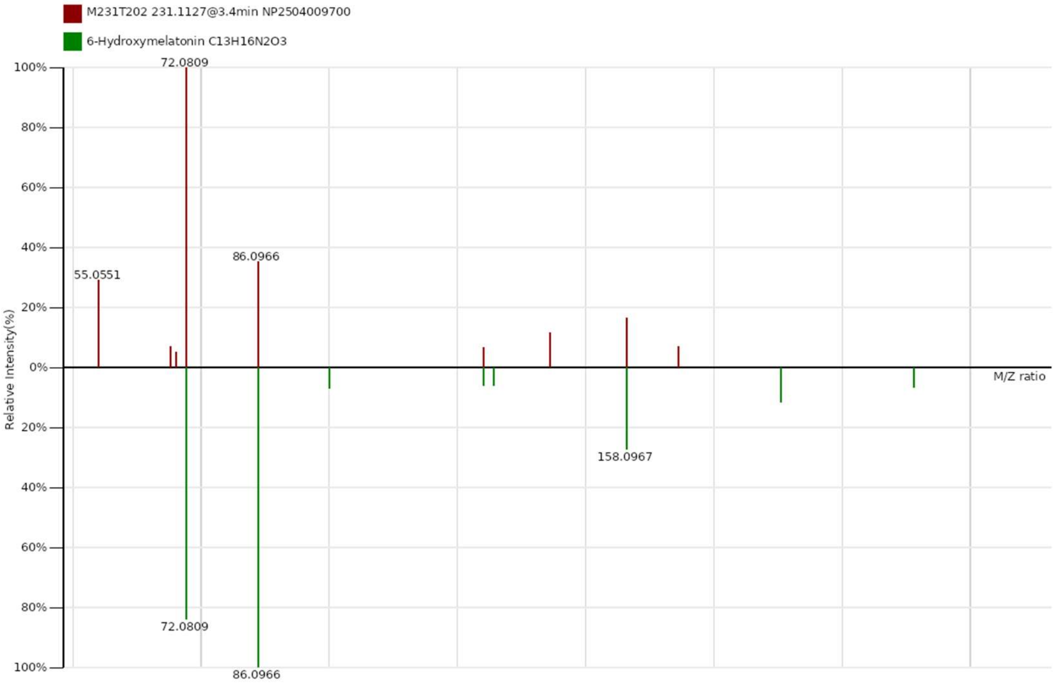
M231T202 231.1127@3.4min NP2504009700
6-Hydroxymelatonin C13H16N2O3
Relative Intensity(%)
M/Z ratio
100%
80%
60%
40%
20%
0%
20%
40%
60%
80%
100%
72.0809
55.0551
86.0966
158.0967
72.0809
86.0966

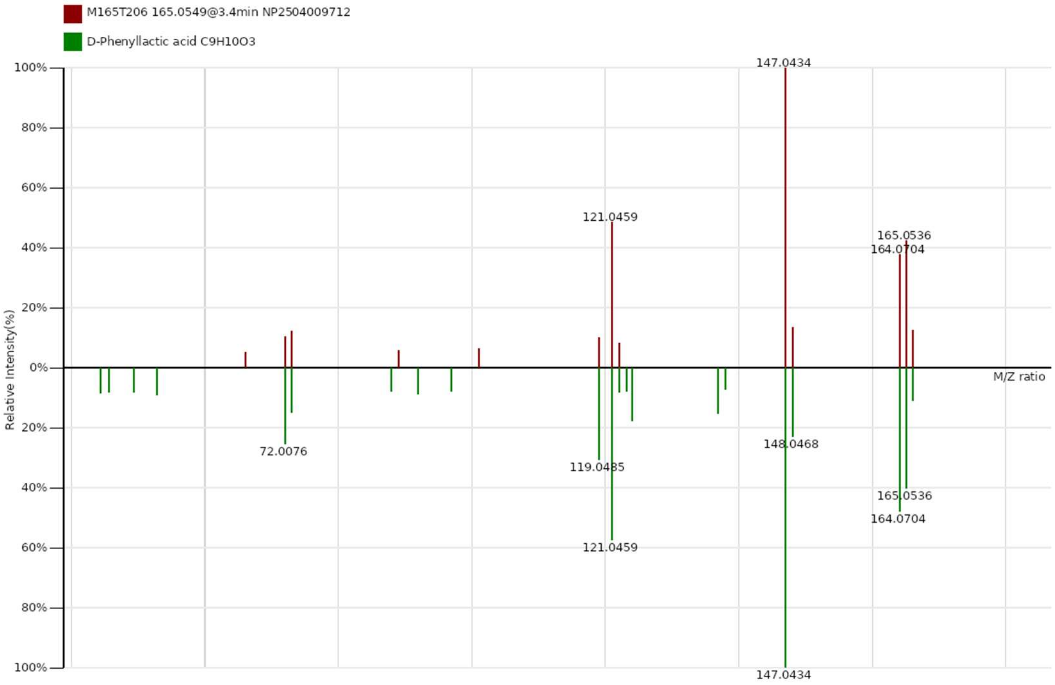
M165T206 165.0549@3.4min NP2504009712
D-Phenyllactic acid C9H10O3
Relative Intensity(%)
M/Z ratio
100%
80%
60%
40%
20%
0%
20%
40%
60%
80%
100%
147.0434
121.0459
165.0536
164.0704
72.0076
119.0485
148.0468
165.0536
164.0704
121.0459
147.0434

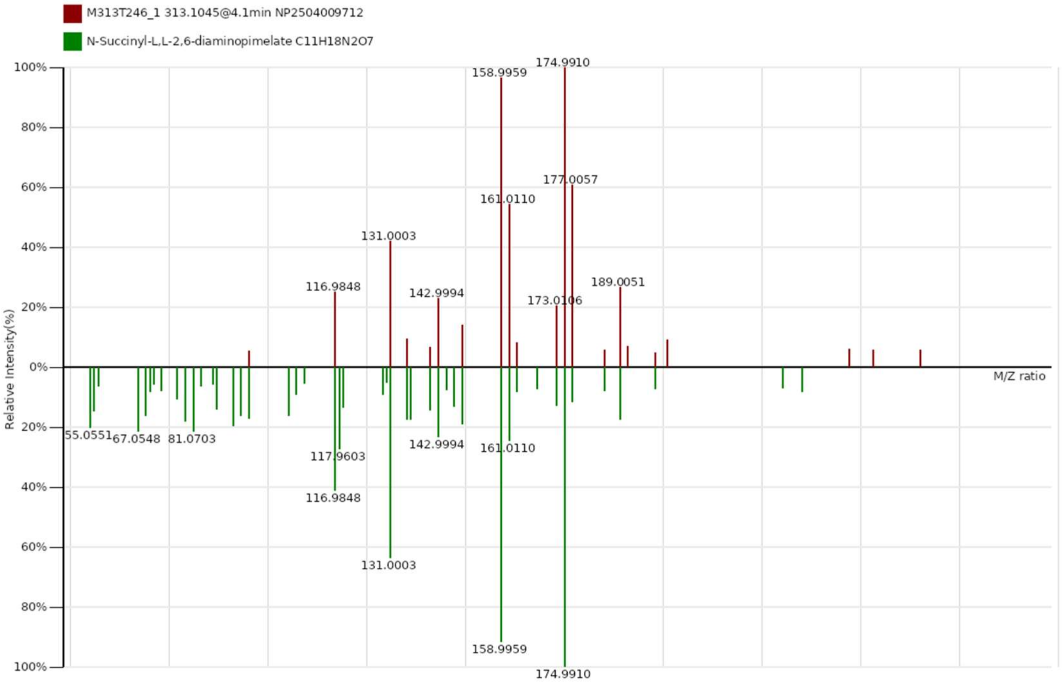

M313T246_1 313.1045@4.1min NP2504009712
N-Succinyl-L,L-2,6-diaminopimelate C11H18N2O7
Relative Intensity(%)
M/Z ratio
100%
80%
60%
40%
20%
0%
158.9959
174.9910
177.0057
161.0110
131.0003
116.9848
142.9994
173.0106
189.0051
55.0551
67.0548
81.0703
117.9603

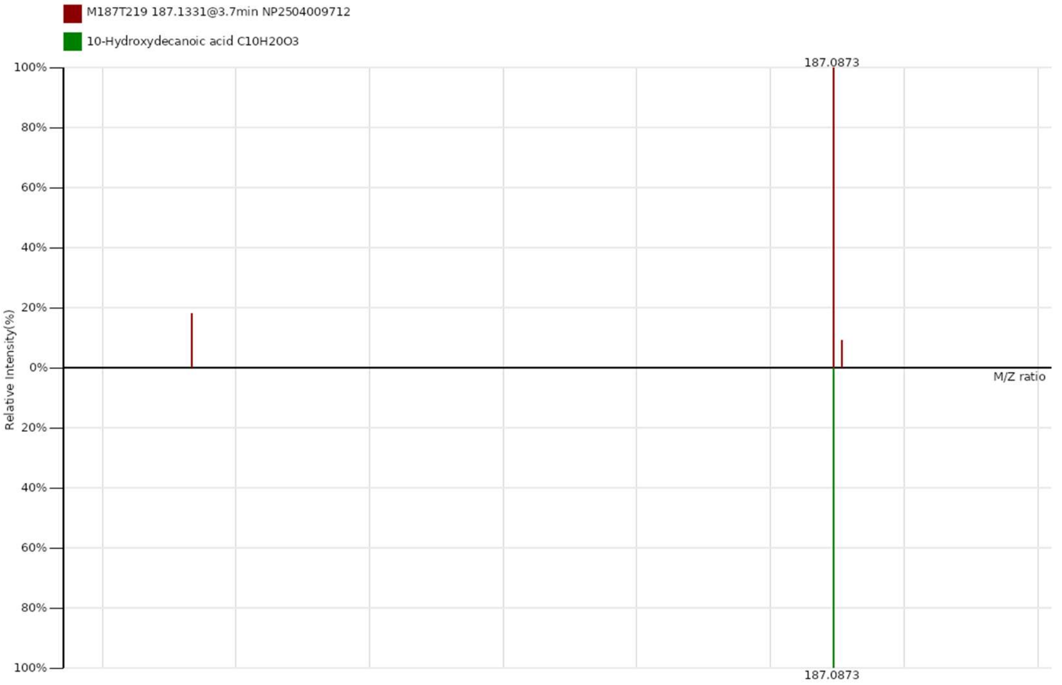

M187T219 187.1331@3.7min NP2504009712
10-Hydroxydecanoic acid C10H20O3
Relative Intensity(%)
M/Z ratio
100%
80%
60%
40%
20%
0%
187.0873

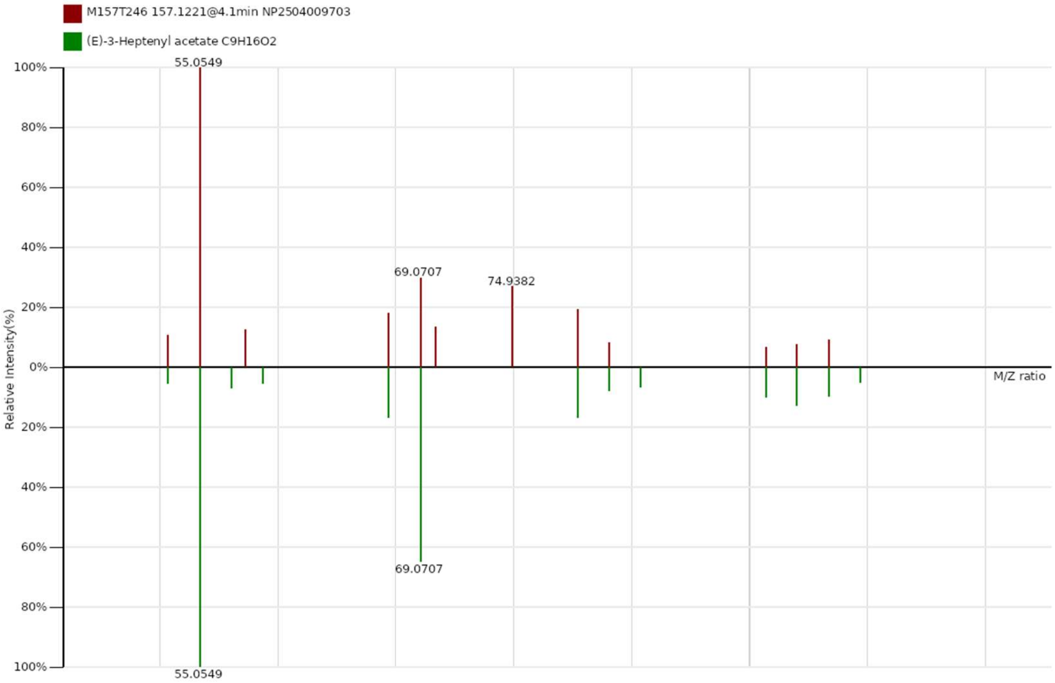
M157T246 157.1221@4.1min NP2504009703
(E)-3-Heptenyl acetate C9H16O2
55.0549
69.0707
74.9382
69.0707
55.0549
Relative Intensity(%)
M/Z ratio
100%
80%
60%
40%
20%
0%
20%
40%
60%
80%
100%

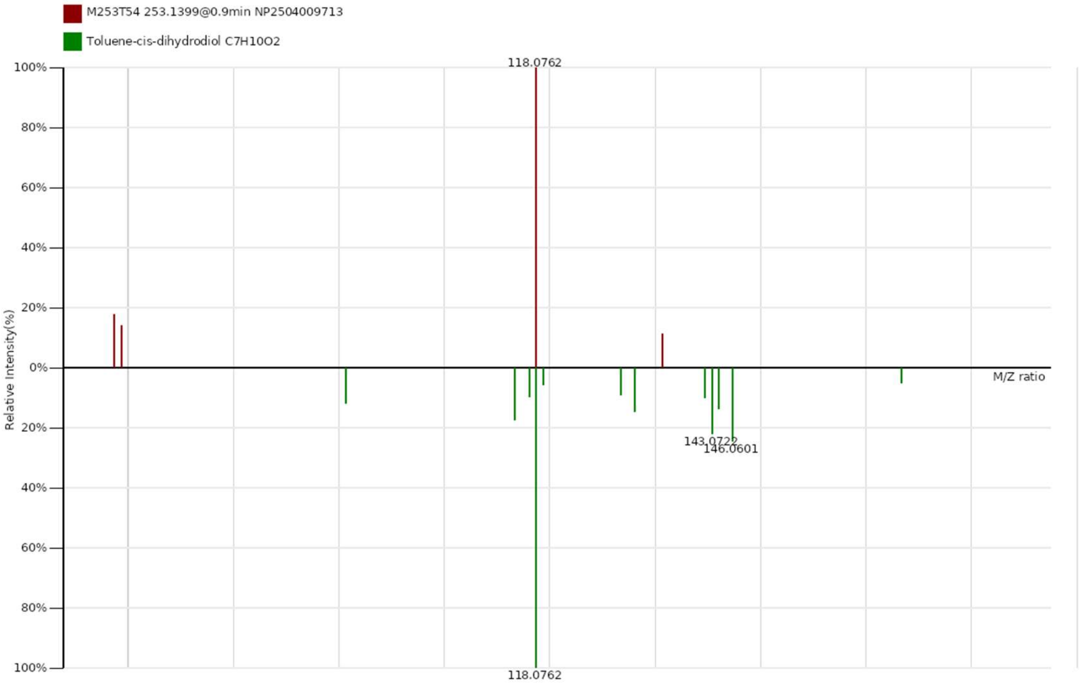
M253T54 253.1399@0.9min NP2504009713
Toluene-cis-dihydrodiol C7H10O2
118.0762
143.0722
146.0601
118.0762
Relative Intensity(%)
M/Z ratio
100%
80%
60%
40%
20%
0%
20%
40%
60%
80%
100%

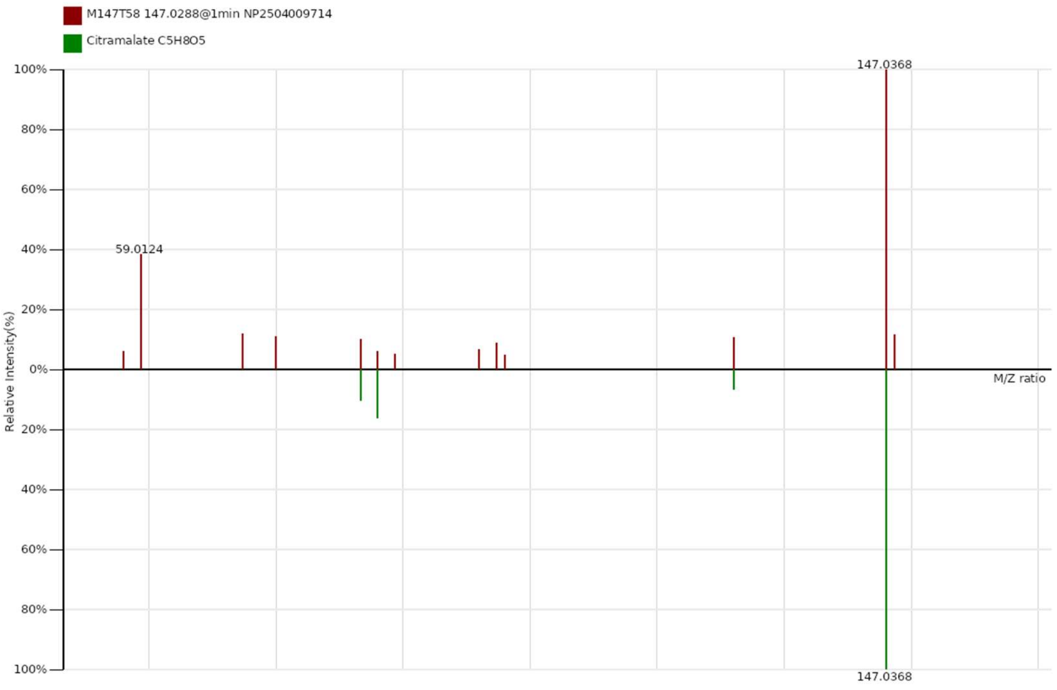
M147T58 147.0288@1min NP2504009714
Citramalate C5H8O5
100%
80%
60%
40%
20%
0%
Relative Intensity(%)
M/Z ratio
147.0368
59.0124
147.0368

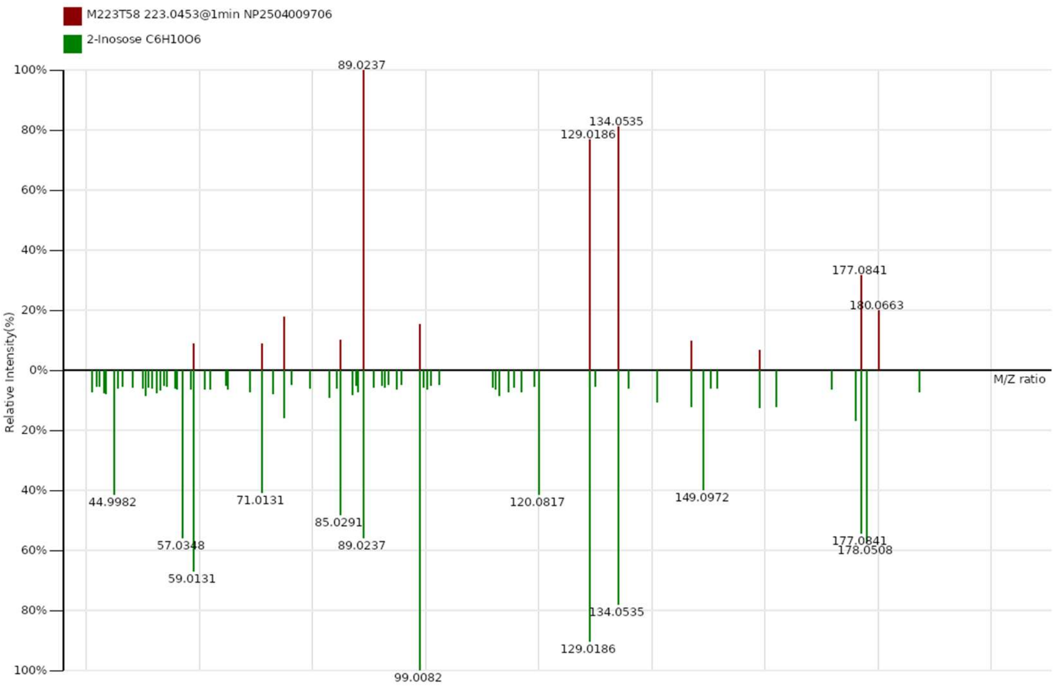
M223T58 223.0453@1min NP2504009706
2-Inosose C6H10O6
100%
80%
60%
40%
20%
0%
Relative Intensity(%)
M/Z ratio
89.0237
134.0535
129.0186
177.0841
180.0663
44.9982
71.0131
120.0817
149.0972
85.0291
57.0348
89.0237
177.0841
178.0508
59.0131
134.0535
129.0186
99.0082

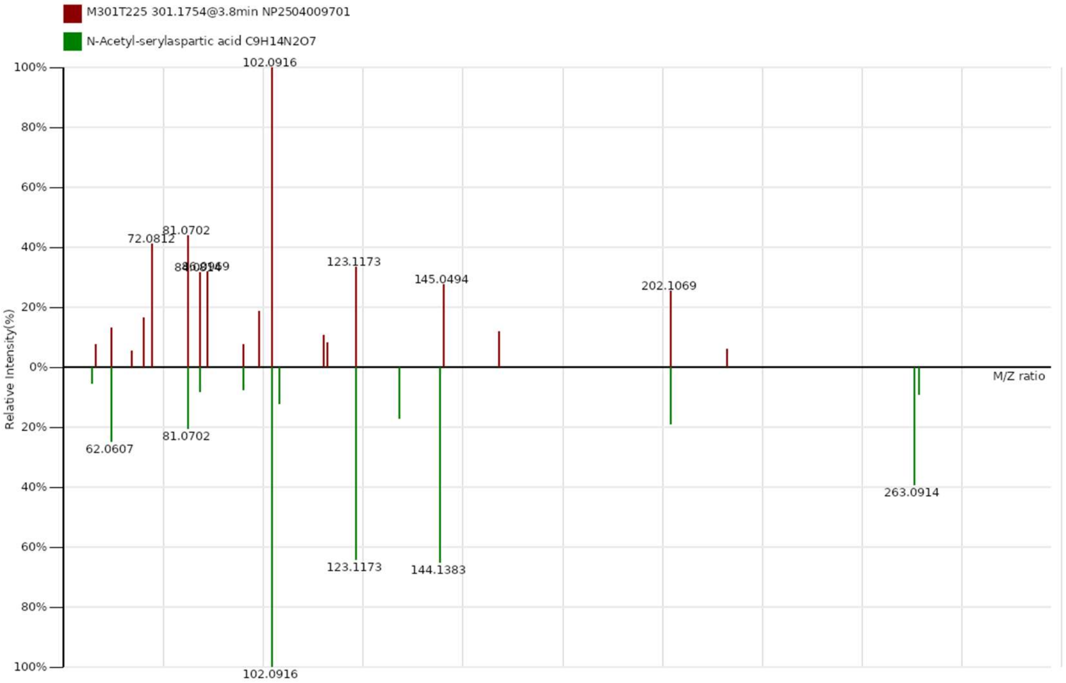
M301T225 301.1754@3.8min NP2504009701
N-Acetyl-serylaspartic acid C9H14N2O7
Relative Intensity(%)
M/Z ratio
100%
80%
60%
40%
20%
0%
20%
40%
60%
80%
100%
102.0916
72.0812
81.0702
123.1173
145.0494
202.1069
62.0607
81.0702
123.1173
144.1383
263.0914
102.0916

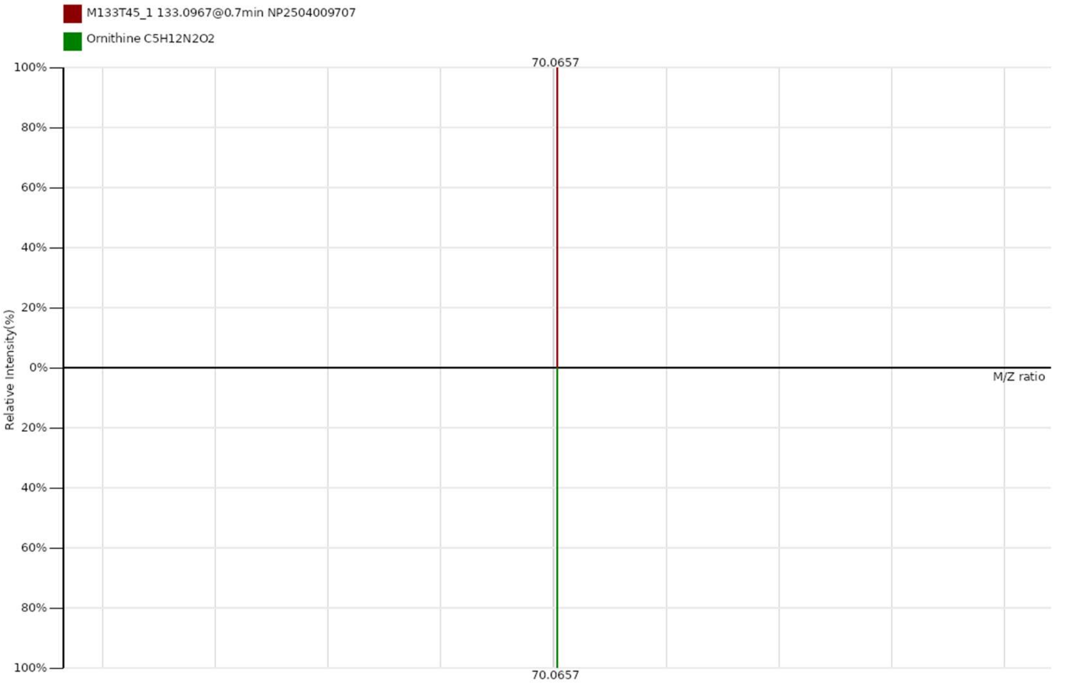
M133T45_1 133.0967@0.7min NP2504009707
Ornithine C5H12N2O2
Relative Intensity(%)
M/Z ratio
100%
80%
60%
40%
20%
0%
20%
40%
60%
80%
100%
70.0657
70.0657

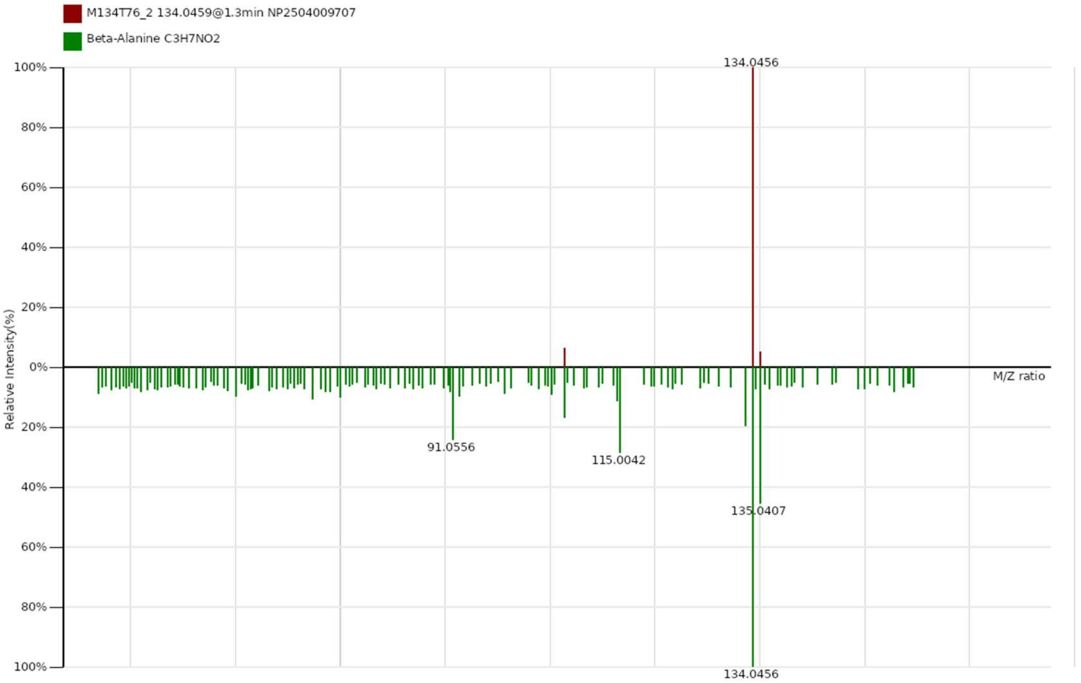
M134T76_2 134.0459@1.3min NP2504009707
Beta-Alanine C3H7NO2
Relative Intensity(%)
M/Z ratio
100%
80%
60%
40%
20%
0%
134.0456
91.0556
115.0042
135.0407
134.0456

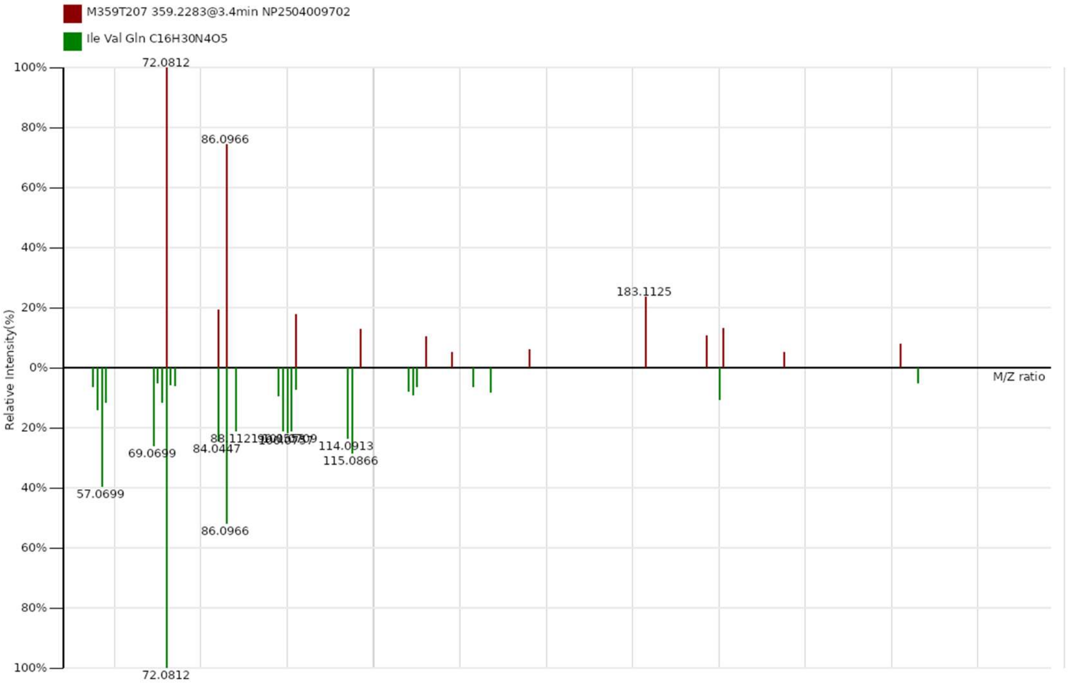
M359T207 359.2283@3.4min NP2504009702
Ile Val Gln C16H30N4O5
Relative Intensity(%)
M/Z ratio
100%
80%
60%
40%
20%
0%
72.0812
86.0966
183.1125
57.0699
69.0699
84.0447
88.1121
114.0913
115.0866
86.0966
72.0812

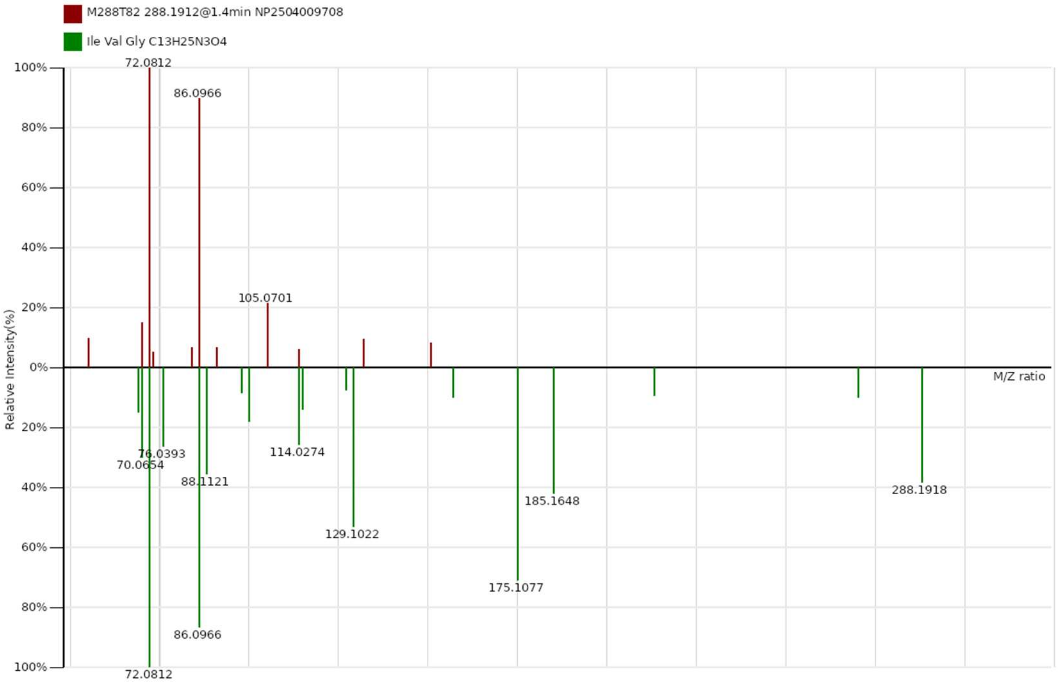
M288T82 288.1912@1.4min NP2504009708
Ile Val Gly C13H25N3O4
Relative Intensity(%)
M/Z ratio
72.0812
86.0966
105.0701
76.0393
70.0654
88.1121
114.0274
129.1022
175.1077
185.1648
288.1918

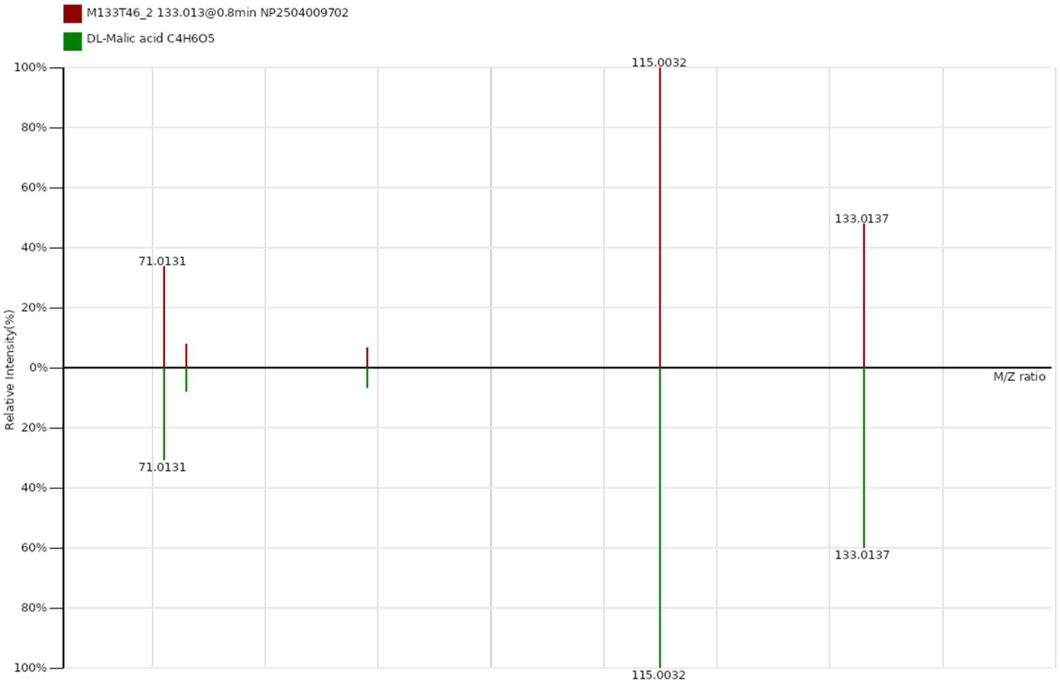
M133T46_2 133.013@0.8min NP2504009702
DL-Malic acid C4H6O5
Relative Intensity(%)
M/Z ratio
115.0032
133.0137
71.0131

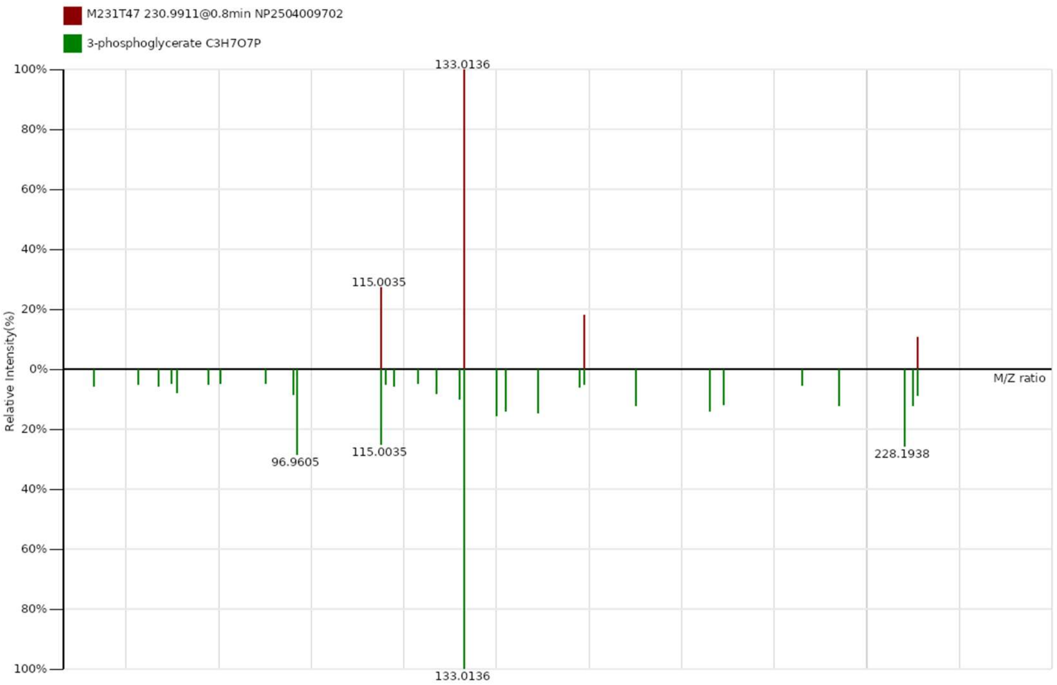
M231T47 230.9911@0.8min NP2504009702
3-phosphoglycerate C3H7O7P
100%
80%
60%
40%
20%
0%
Relative Intensity(%)
M/Z ratio
133.0136
115.0035
96.9605
115.0035
228.1938
133.0136

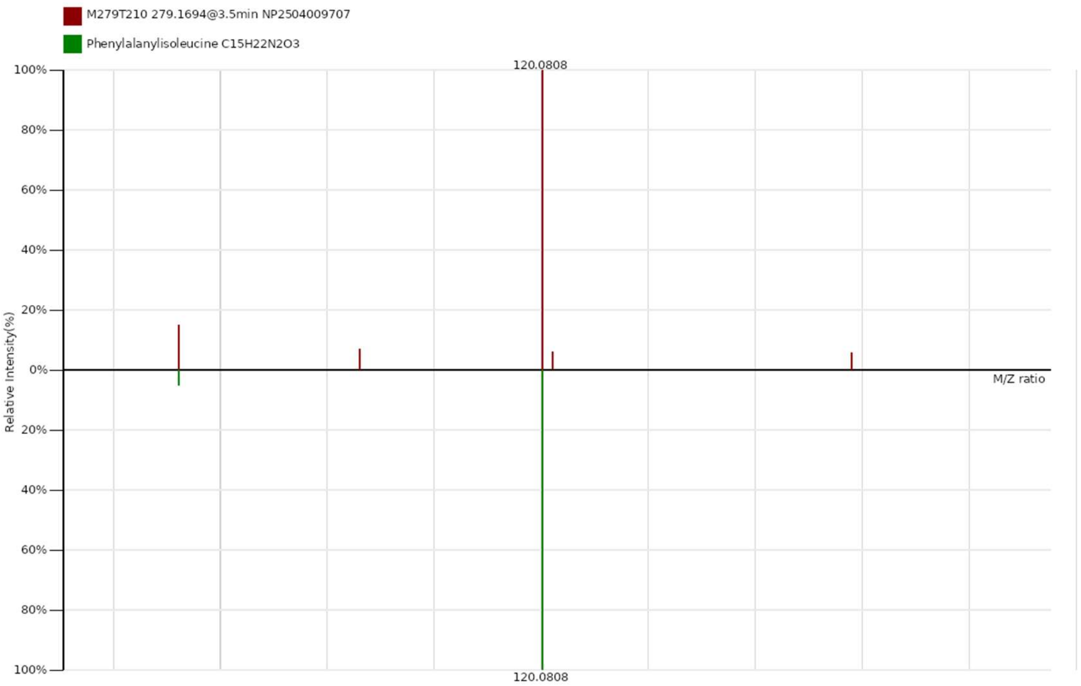
M279T210 279.1694@3.5min NP2504009707
Phenylalanylisoleucine C15H22N2O3
100%
80%
60%
40%
20%
0%
Relative Intensity(%)
M/Z ratio
120.0808
120.0808

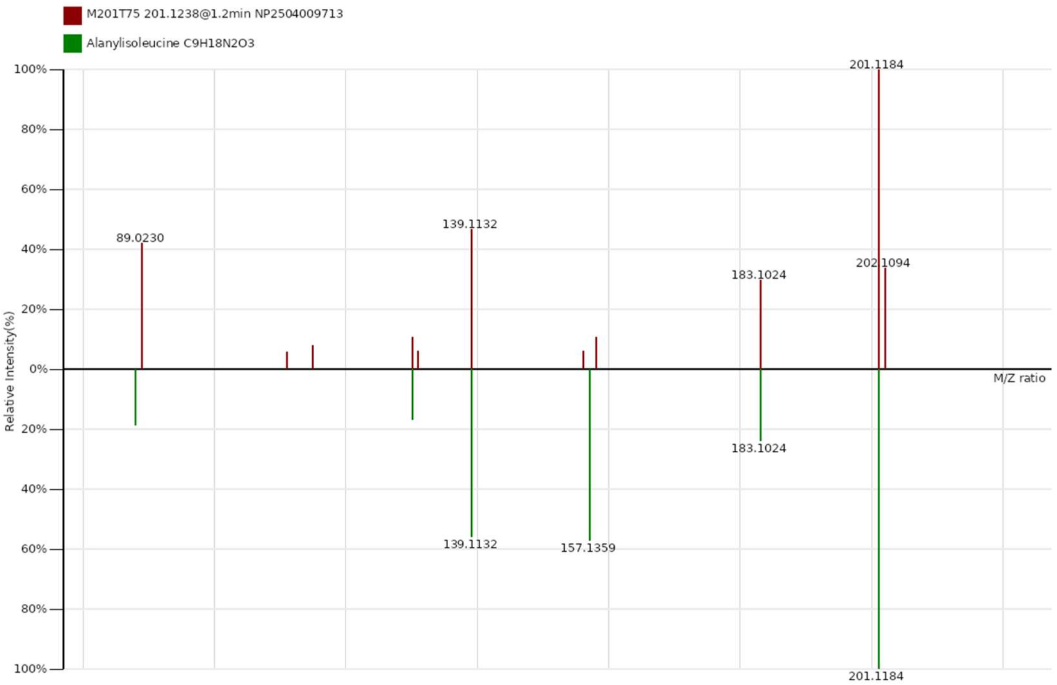
M201T75 201.1238@1.2min NP2504009713
Alanylisoleucine C9H18N2O3
100%
80%
60%
40%
20%
0%
20%
40%
60%
80%
100%
Relative Intensity(%)
M/Z ratio
89.0230
139.1132
183.1024
201.1184
202.1094
139.1132
157.1359
183.1024
201.1184

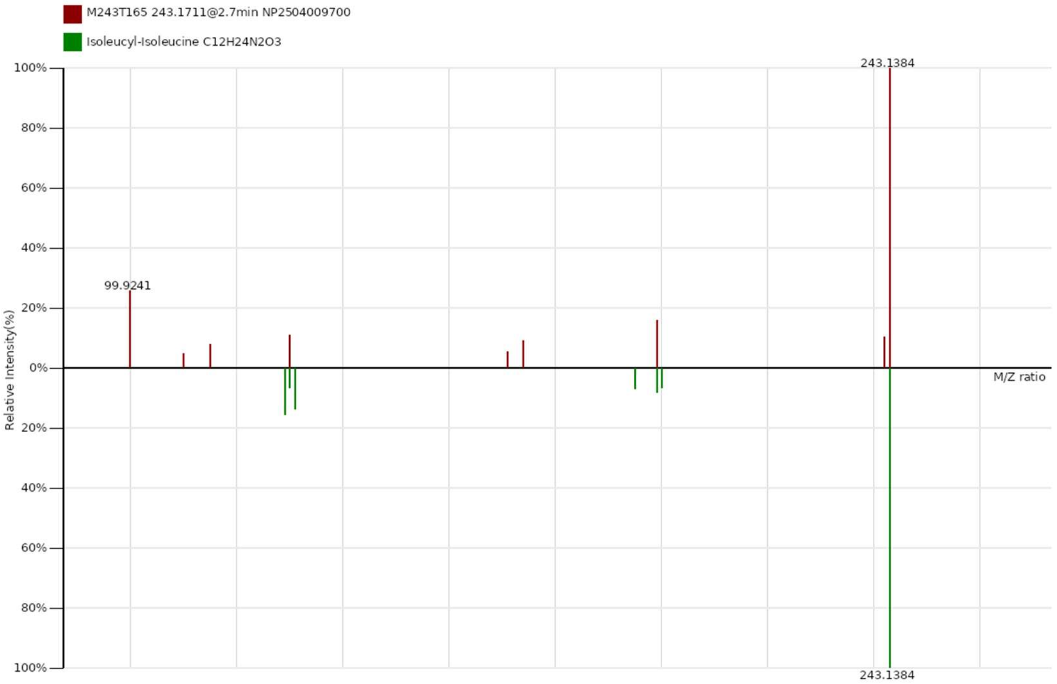
M243T165 243.1711@2.7min NP2504009700
Isoleucyl-Isoleucine C12H24N2O3
100%
80%
60%
40%
20%
0%
20%
40%
60%
80%
100%
Relative Intensity(%)
M/Z ratio
99.9241
243.1384
243.1384

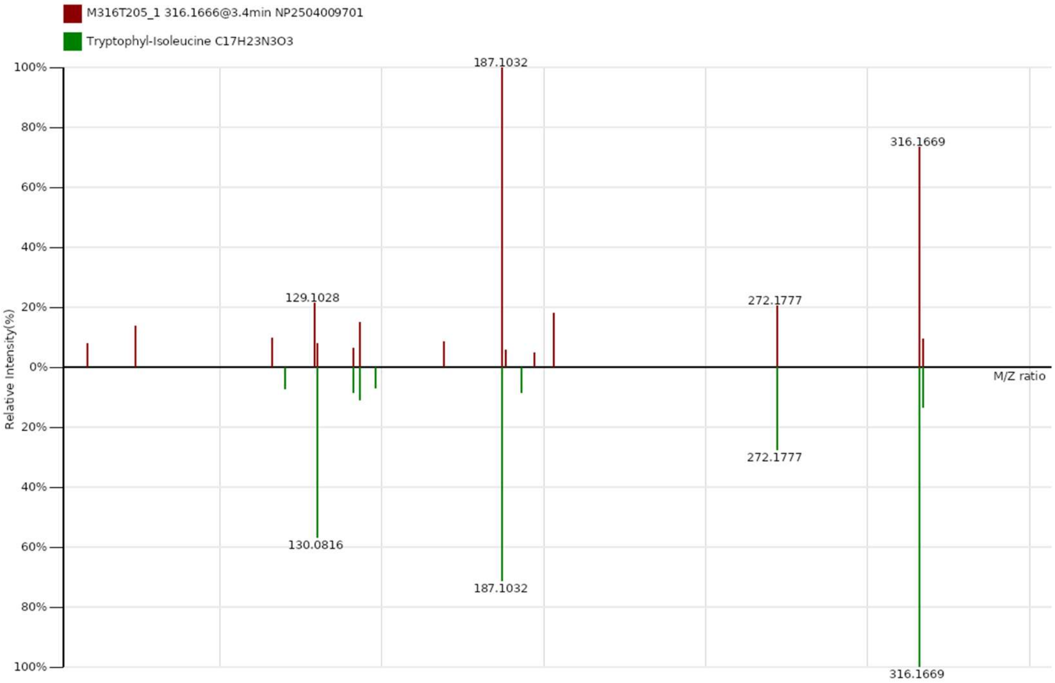
M316T205_1 316.1666@3.4min NP2504009701
Tryptophyl-Isoleucine C17H23N3O3
100%
80%
60%
40%
20%
0%
20%
40%
60%
80%
100%
Relative Intensity(%)
M/Z ratio
187.1032
316.1669
129.1028
272.1777
130.0816
187.1032
272.1777
316.1669

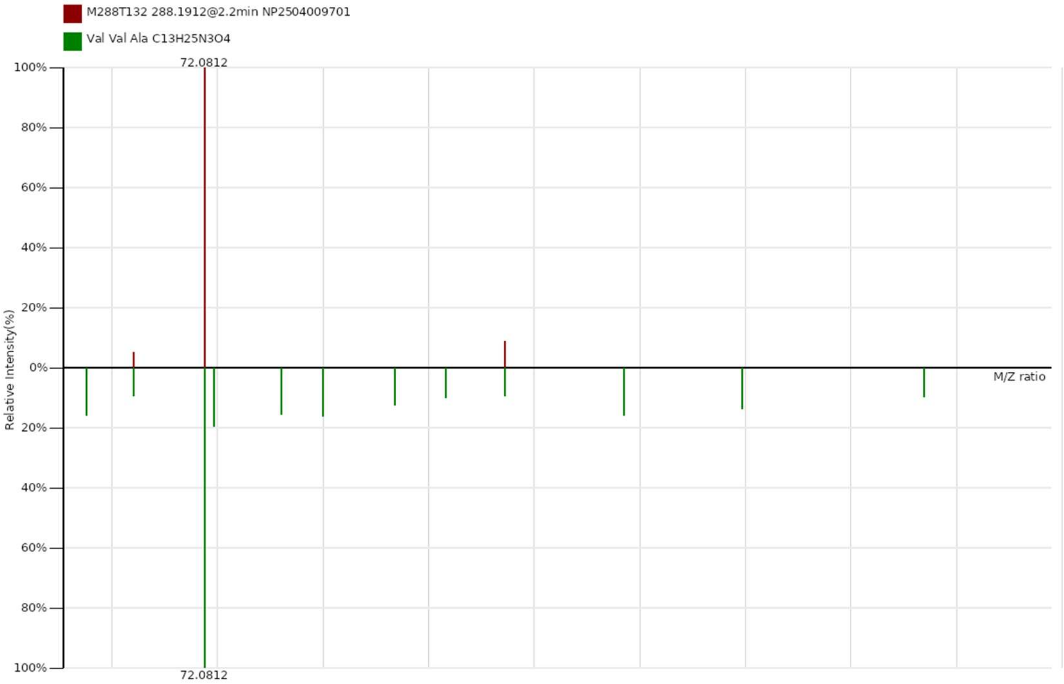
M288T132 288.1912@2.2min NP2504009701
Val Val Ala C13H25N3O4
100%
80%
60%
40%
20%
0%
20%
40%
60%
80%
100%
Relative Intensity(%)
M/Z ratio
72.0812
72.0812

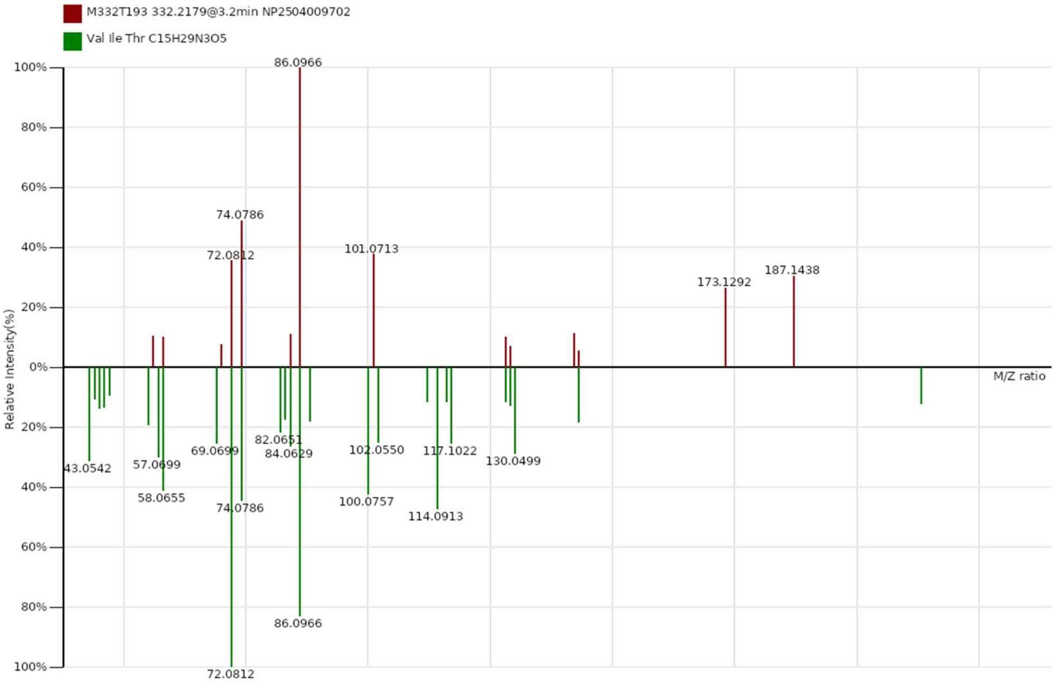
M332T193 332.2179@3.2min NP2504009702
Val Ile Thr C15H29N3O5
Relative Intensity(%)
M/Z ratio
100%
80%
60%
40%
20%
0%
86.0966
74.0786
72.0812
101.0713
173.1292
187.1438
43.0542
57.0699
58.0655
69.0699
82.0651
84.0629
102.0550
100.0757
117.1022
114.0913
130.0499

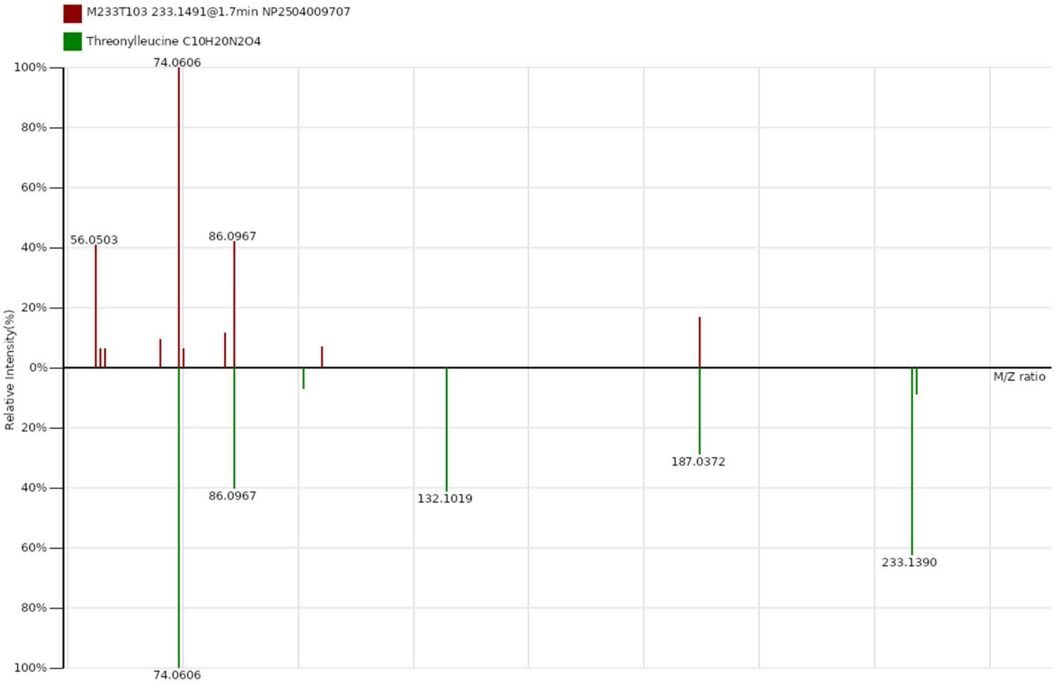
M233T103 233.1491@1.7min NP2504009707
Threonylleucine C10H20N2O4
Relative Intensity(%)
M/Z ratio
100%
80%
60%
40%
20%
0%
74.0606
56.0503
86.0967
132.1019
187.0372
233.1390

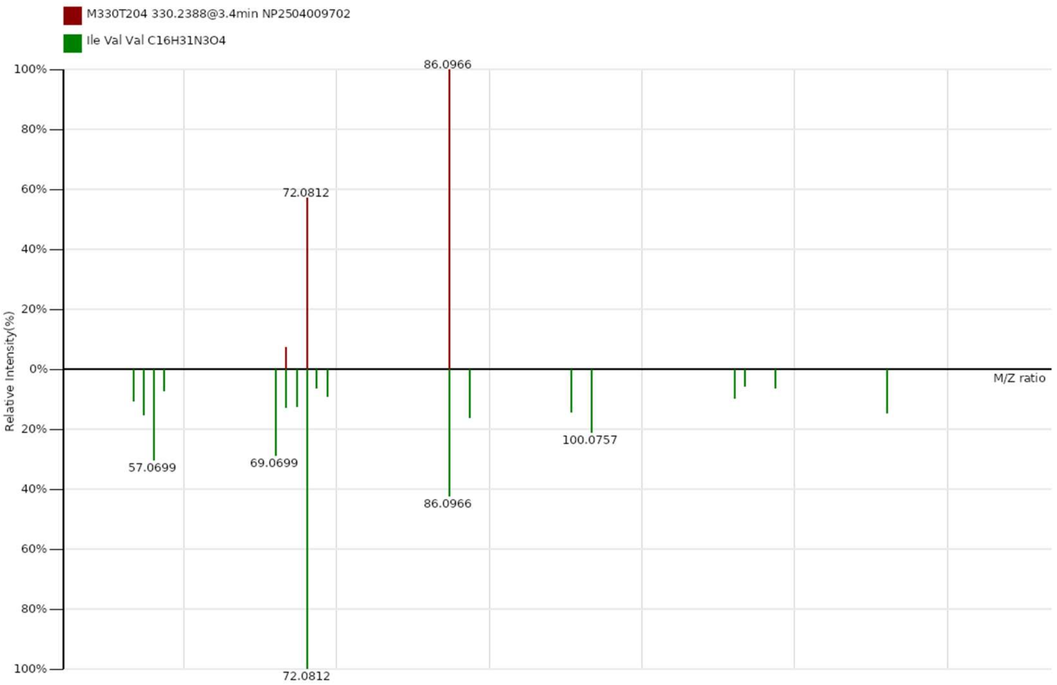

M330T204 330.2388@3.4min NP2504009702
Ile Val Val C16H31N3O4
100%
80%
60%
40%
20%
0%
20%
40%
60%
80%
100%
Relative Intensity(%)
M/Z ratio
86.0966
72.0812
57.0699
69.0699
86.0966
100.0757
72.0812

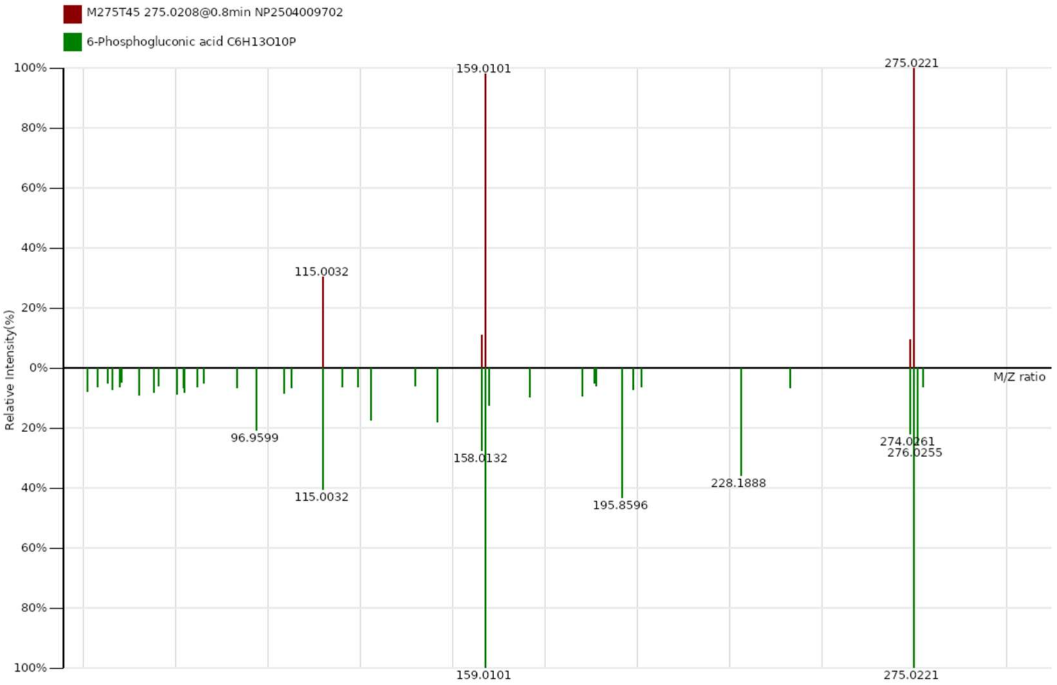

M275T45 275.0208@0.8min NP2504009702
6-Phosphogluconic acid C6H13O10P
100%
80%
60%
40%
20%
0%
20%
40%
60%
80%
100%
Relative Intensity(%)
M/Z ratio
159.0101
275.0221
115.0032
96.9599
115.0032
158.0132
195.8596
228.1888
274.0261
276.0255
159.0101
275.0221

Experimental fragments of the top 30 significant differential metabolites (DMs) from the comparison of BS48H vs. BS24H.

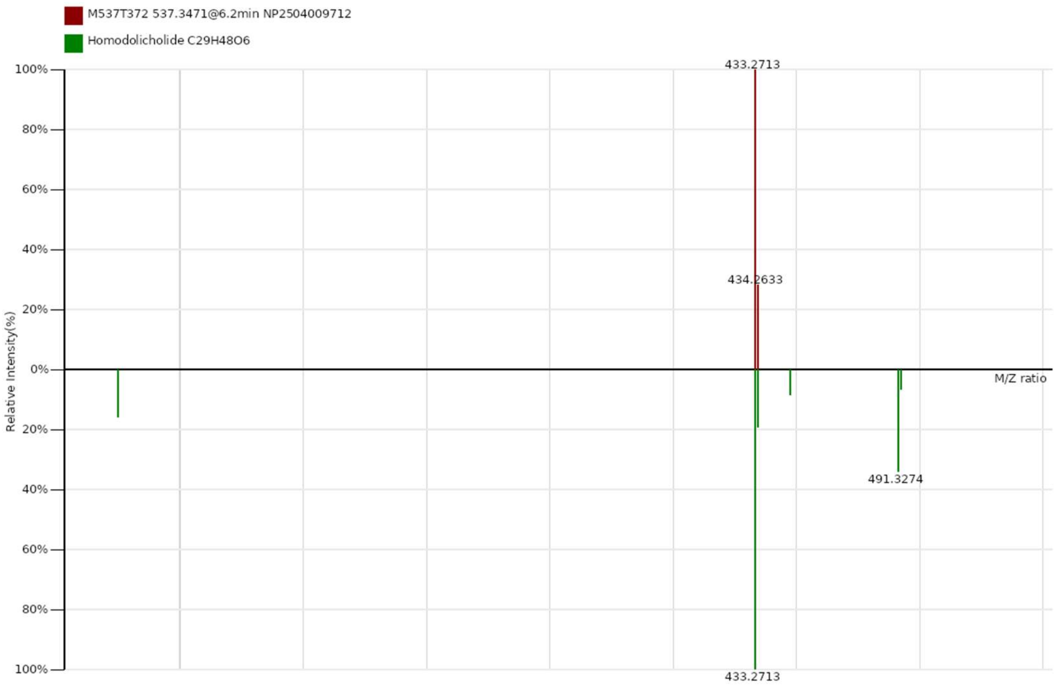

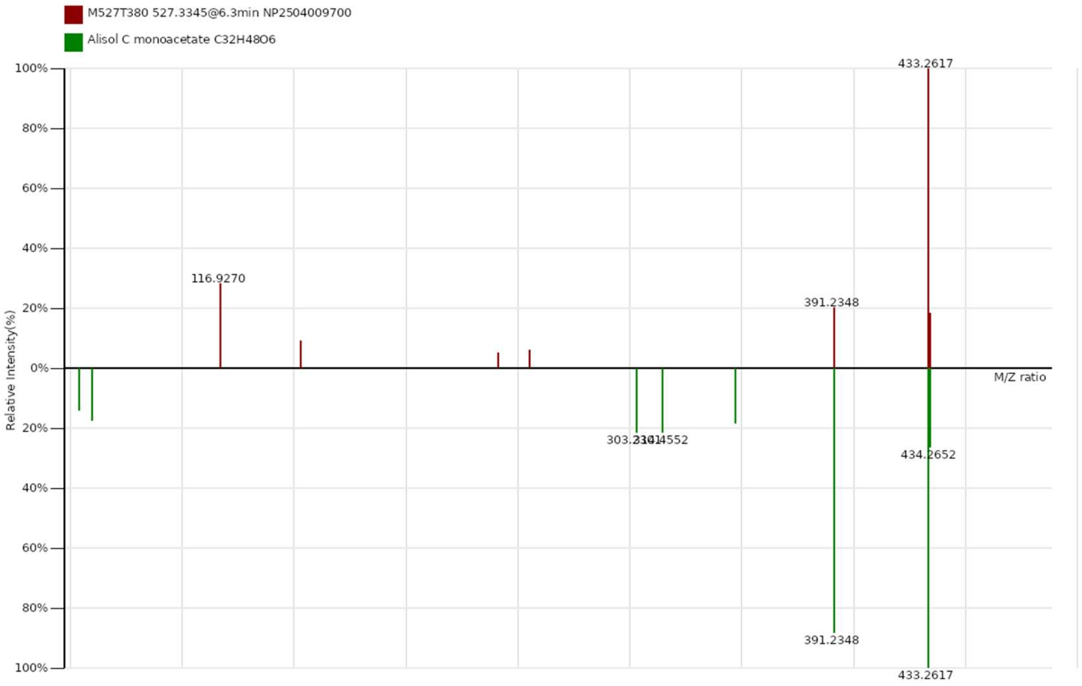

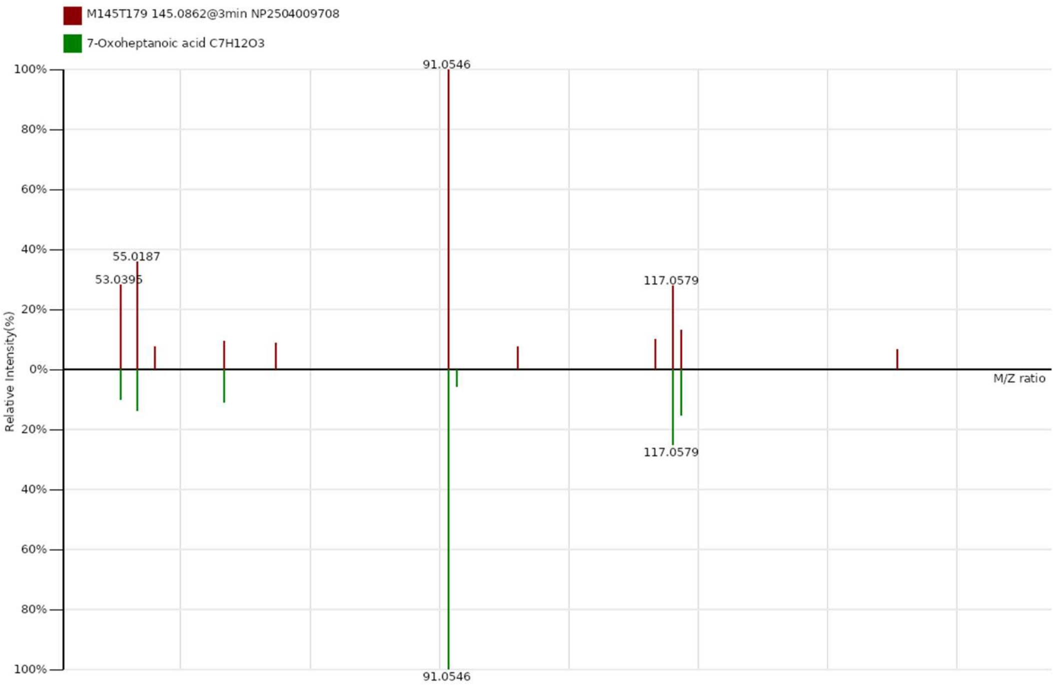

M145T179 145.0862@3min NP2504009708
7-Oxoheptanoic acid C7H12O3
91.0546
55.0187
53.0395
117.0579
117.0579
91.0546
Relative Intensity(%)
M/Z ratio

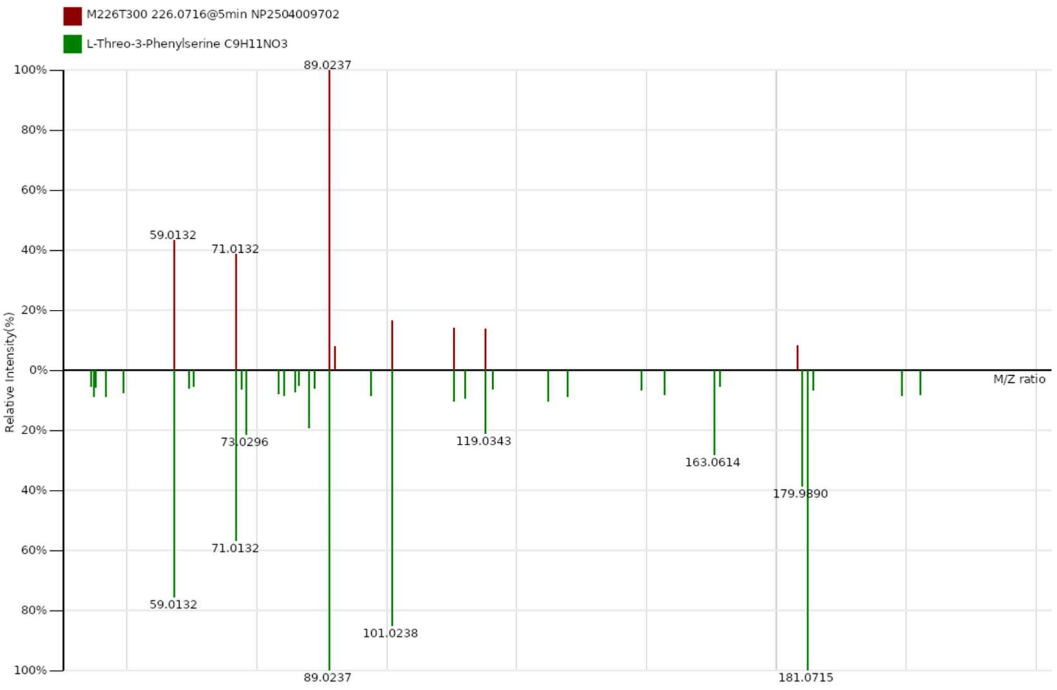

M226T300 226.0716@5min NP2504009702
L-Threo-3-Phenylserine C9H11NO3
89.0237
59.0132
71.0132
73.0296
119.0343
163.0614
179.9890
71.0132
59.0132
101.0238
89.0237
181.0715
Relative Intensity(%)
M/Z ratio

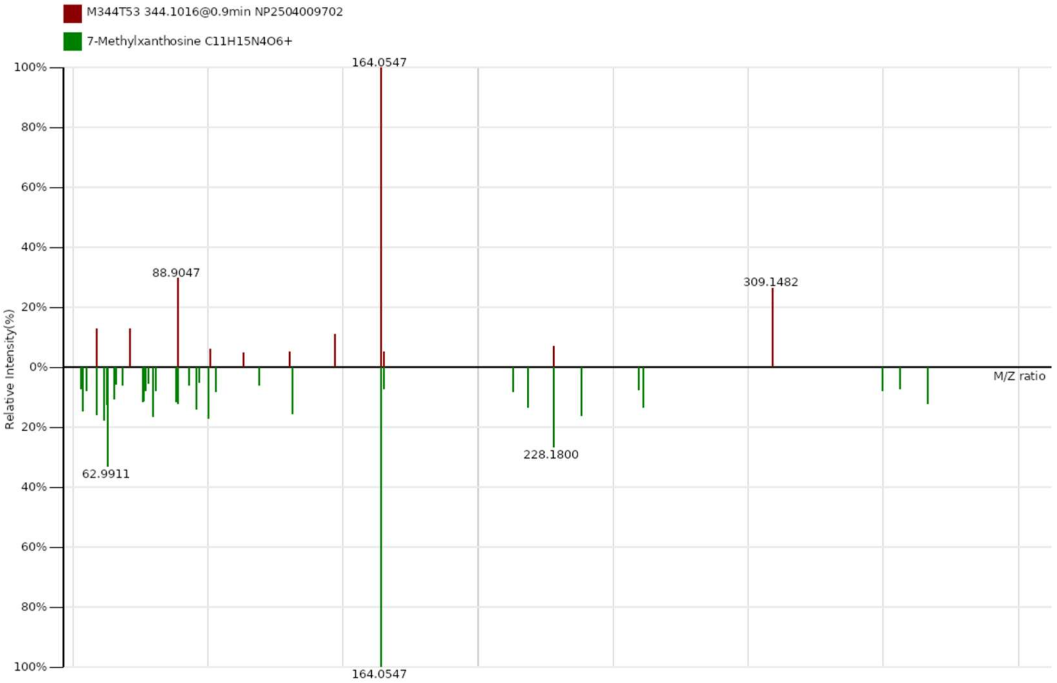
M344T53 344.1016@0.9min NP2504009702
7-Methylxanthosine C11H15N4O6+
Relative Intensity(%)
M/Z ratio
164.0547
88.9047
309.1482
62.9911
228.1800
164.0547

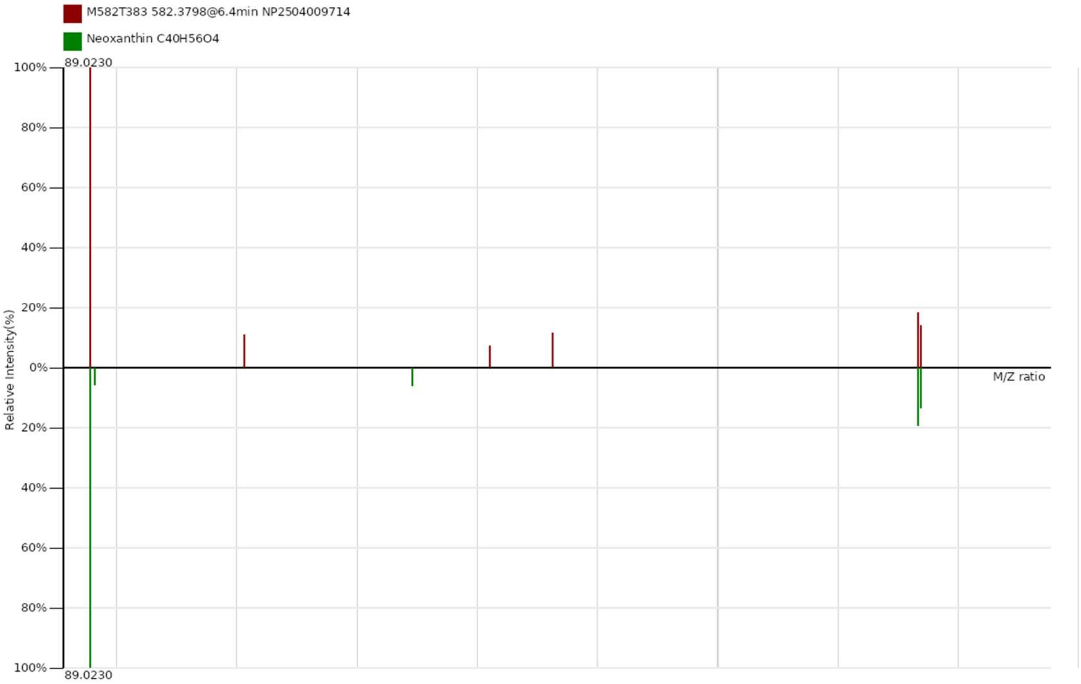
M582T383 582.3798@6.4min NP2504009714
Neoxanthin C40H56O4
Relative Intensity(%)
M/Z ratio
89.0230
89.0230

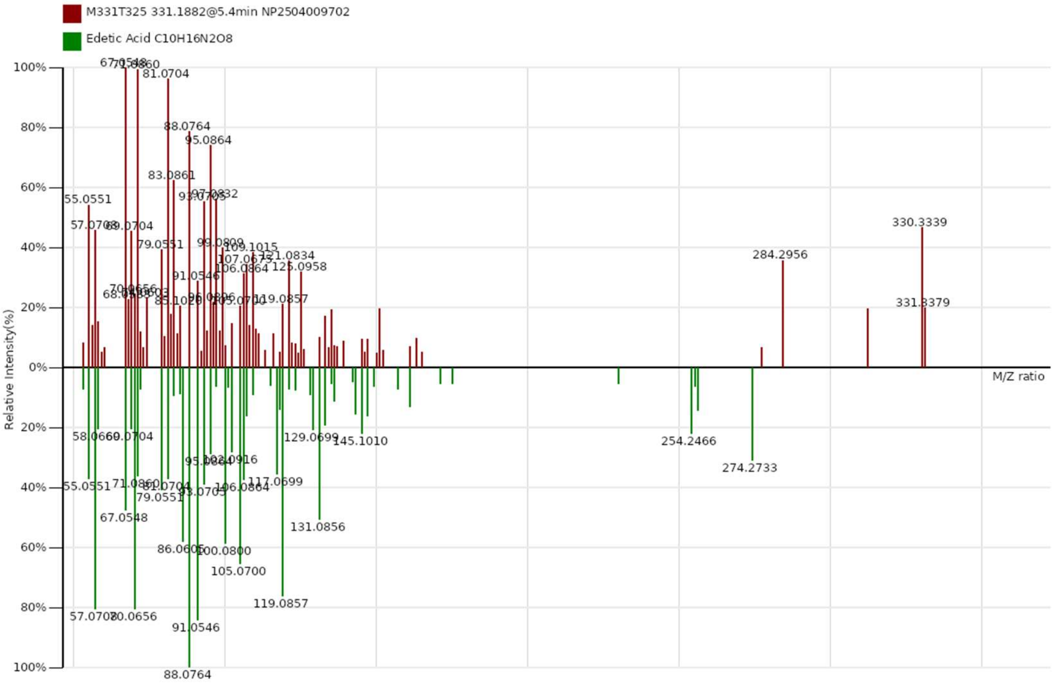

M331T325 331.1882@5.4min NP2504009702
Edetic Acid C10H16N2O8
Relative Intensity(%)
M/Z ratio
81.0704
88.0764
95.0864
83.0861
55.0551
109.1015
121.0834
125.0958
119.0857
330.3339
284.2956
331.3379
129.0699
145.1010
254.2466
274.2733
67.0548
131.0856
86.0605
100.0800
105.0700
119.0857
57.0708
80.0656
91.0546
88.0764

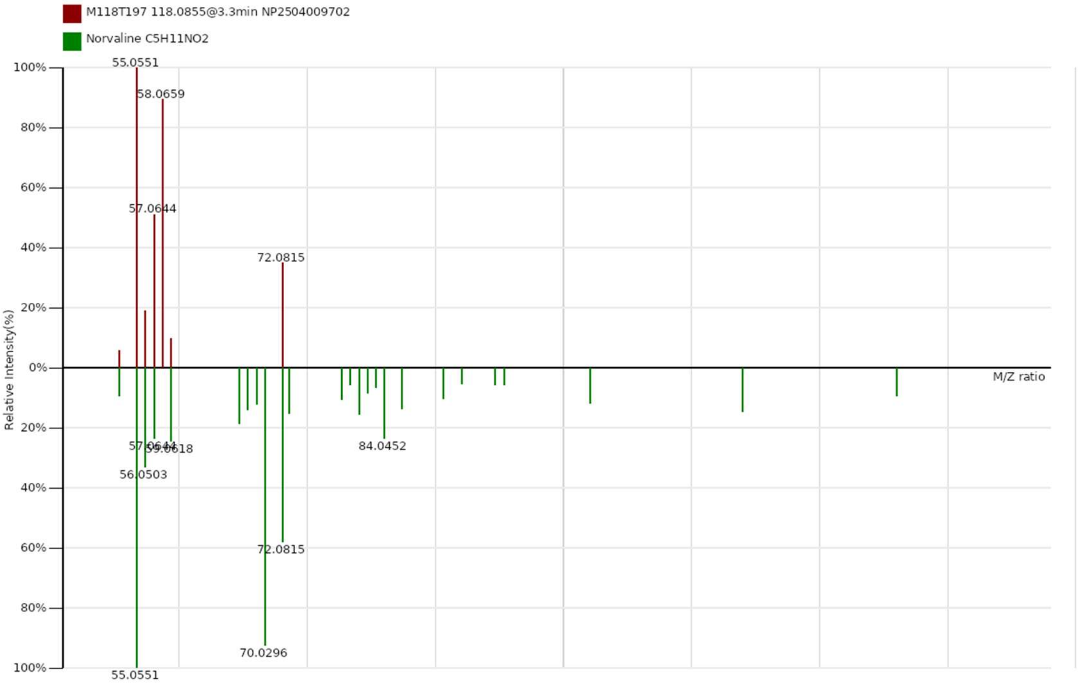

M118T197 118.0855@3.3min NP2504009702
Norvaline C5H11NO2
Relative Intensity(%)
M/Z ratio
55.0551
58.0659
57.0644
72.0815
84.0452
56.0503
72.0815
70.0296
55.0551

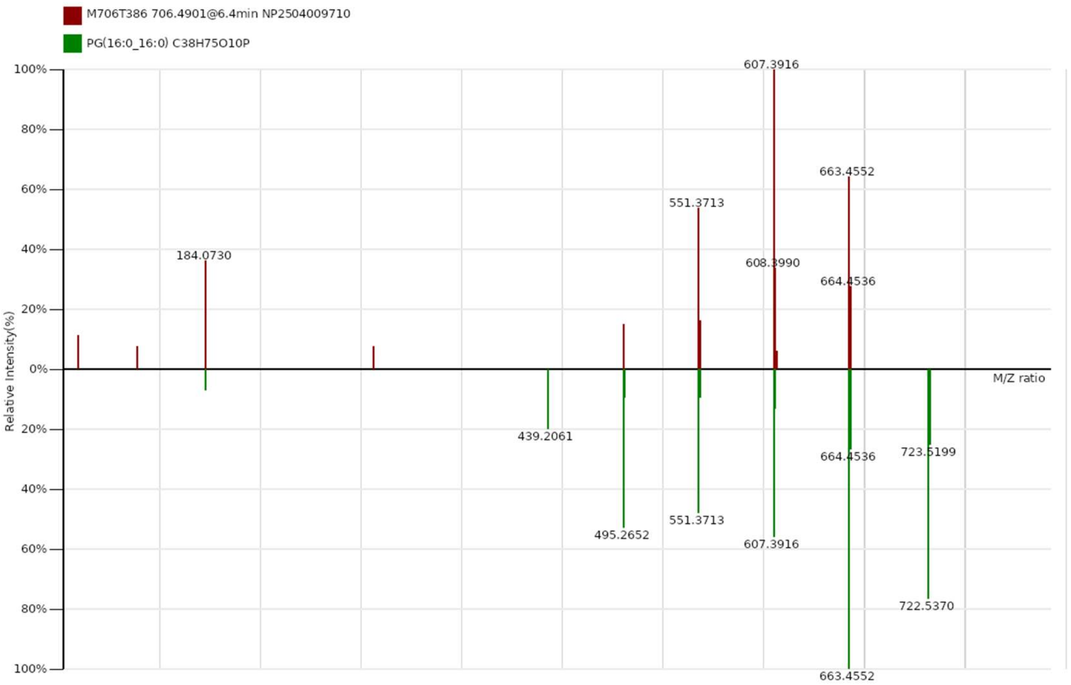
M706T386 706.4901@6.4min NP2504009710
PG(16:0_16:0) C38H75O10P
Relative Intensity(%)
M/Z ratio
100%
80%
60%
40%
20%
0%
607.3916
663.4552
551.3713
184.0730
608.3990
664.4536
439.2061
664.4536
723.5199
495.2652
551.3713
607.3916
722.5370
663.4552

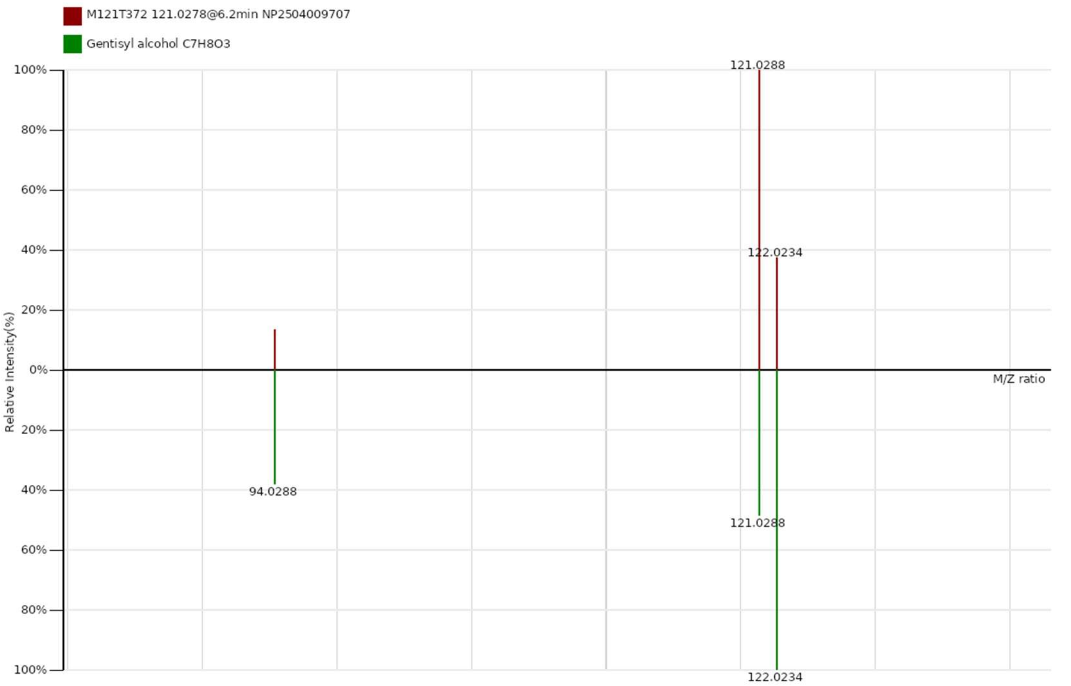
M121T372 121.0278@6.2min NP2504009707
Gentisyl alcohol C7H8O3
Relative Intensity(%)
M/Z ratio
100%
80%
60%
40%
20%
0%
121.0288
122.0234
94.0288
121.0288
122.0234

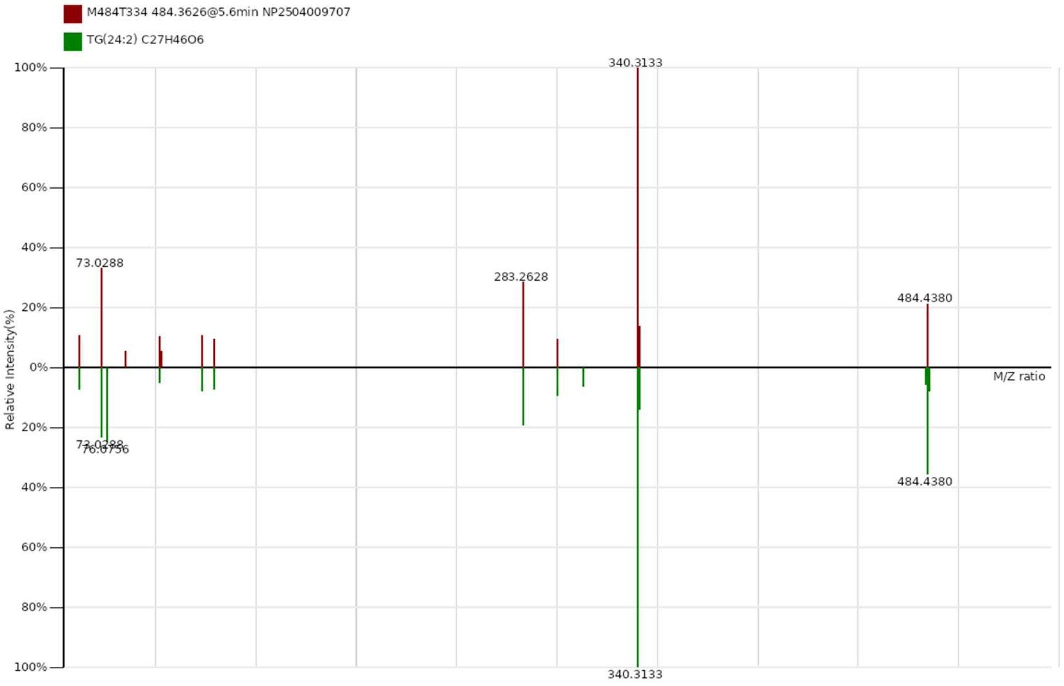

M484T334 484.3626@5.6min NP2504009707
TG(24:2) C27H46O6
100%
80%
60%
40%
20%
0%
20%
40%
60%
80%
100%
Relative Intensity(%)
M/Z ratio
73.0288
283.2628
340.3133
484.4380
73.0288
76.0756
484.4380
340.3133

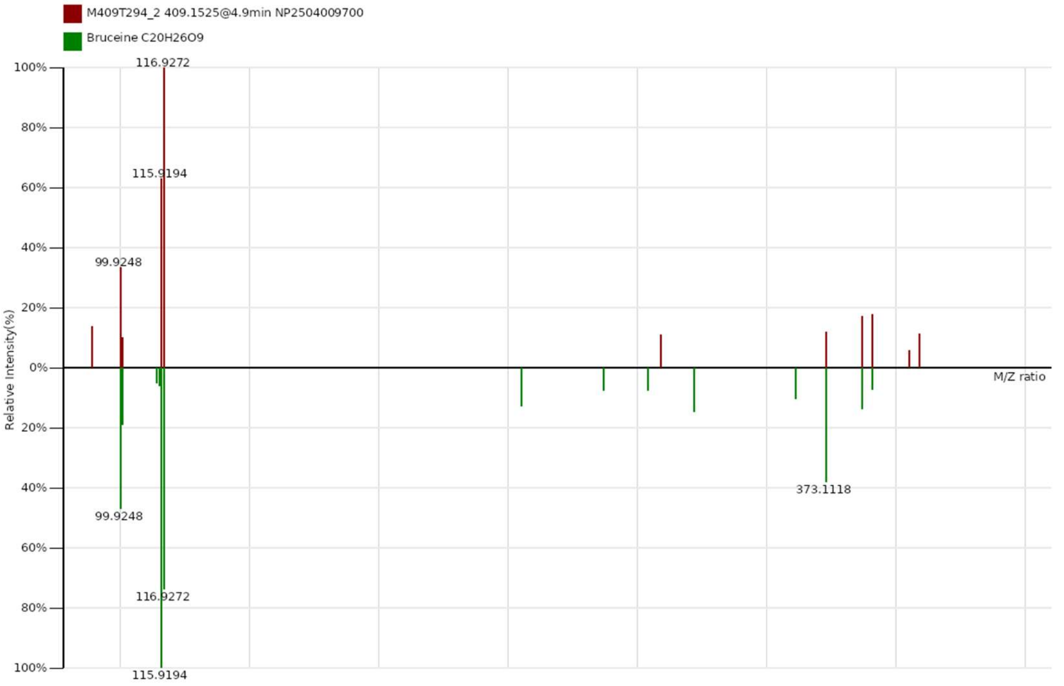

M409T294_2 409.1525@4.9min NP2504009700
Bruceine C20H26O9
100%
80%
60%
40%
20%
0%
20%
40%
60%
80%
100%
Relative Intensity(%)
M/Z ratio
116.9272
115.9194
99.9248
99.9248
373.1118
116.9272
115.9194

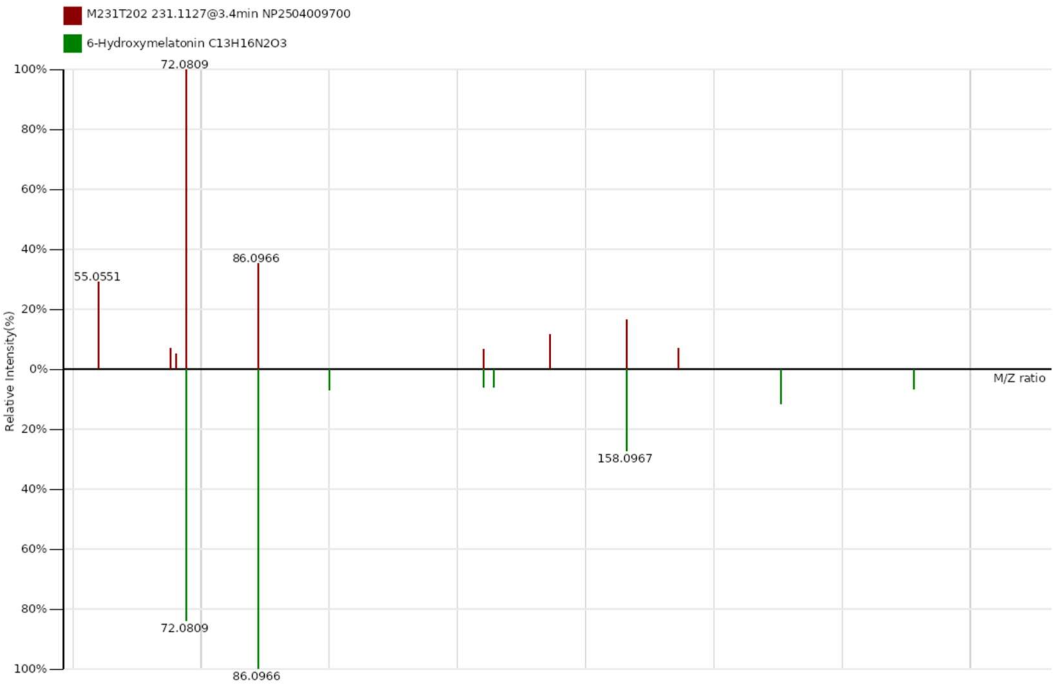
M231T202 231.1127@3.4min NP2504009700
6-Hydroxymelatonin C13H16N2O3
100%
80%
60%
40%
20%
0%
20%
40%
60%
80%
100%
Relative Intensity(%)
M/Z ratio
72.0809
55.0551
86.0966
158.0967
72.0809
86.0966

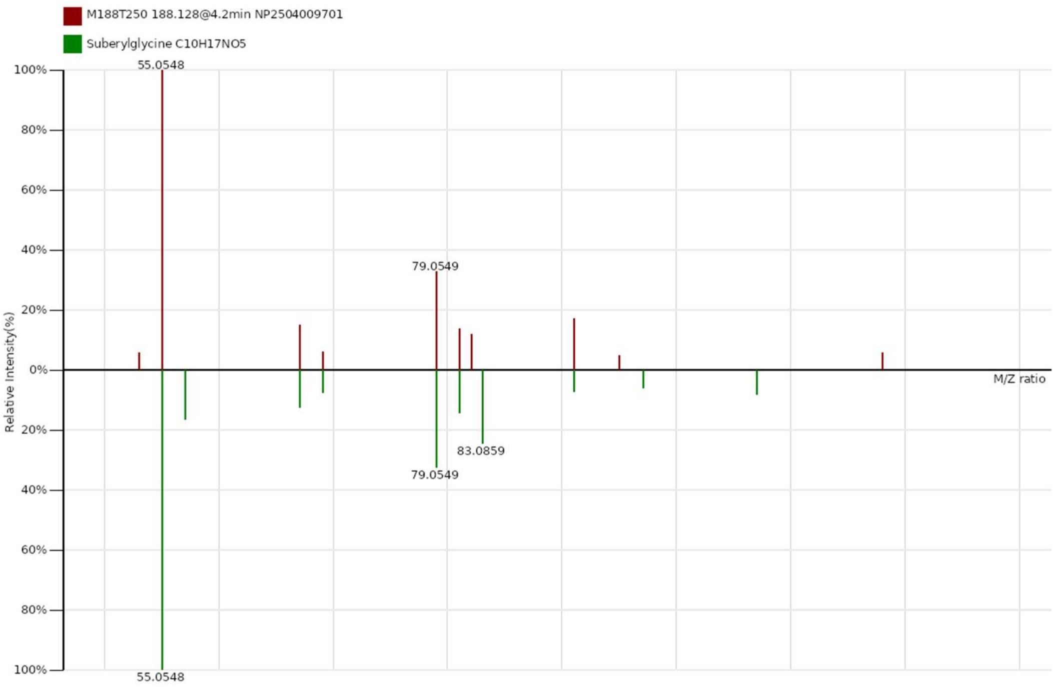
M188T250 188.128@4.2min NP2504009701
Suberylglycine C10H17NO5
100%
80%
60%
40%
20%
0%
20%
40%
60%
80%
100%
Relative Intensity(%)
M/Z ratio
55.0548
79.0549
83.0859
79.0549
55.0548

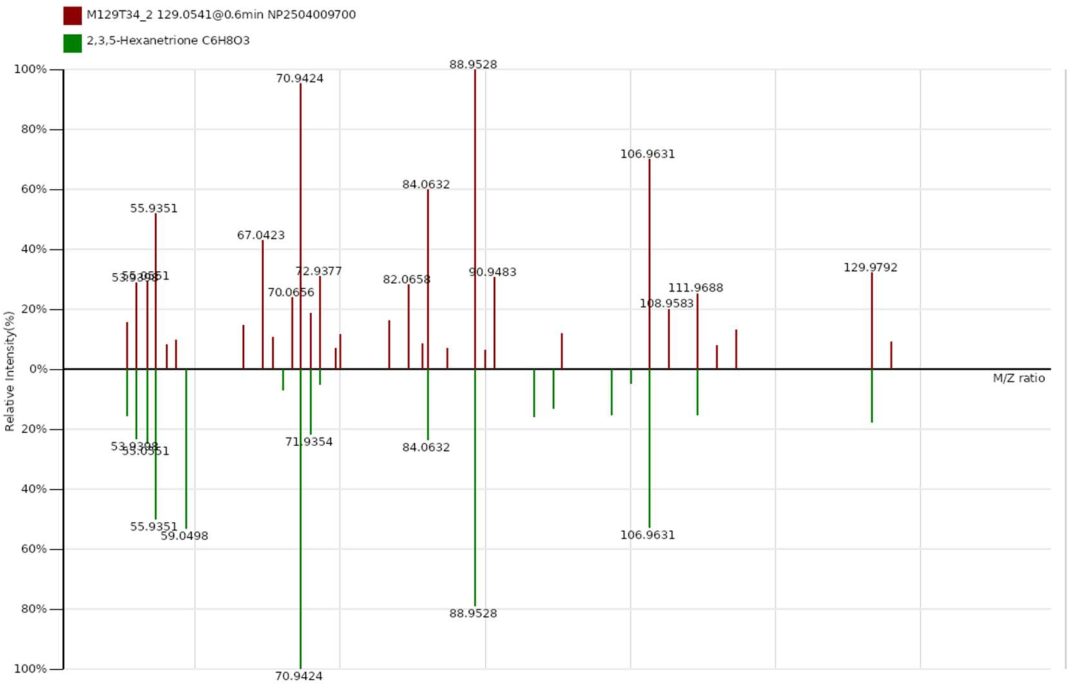
M129T34_2 129.0541@0.6min NP2504009700
2,3,5-Hexanetrione C6H8O3
Relative Intensity(%)
M/Z ratio
70.9424
88.9528
106.9631
84.0632
55.9351
67.0423
72.9377
82.0658
90.9483
129.9792
111.9688
70.0656
108.9583
71.9354
59.0498

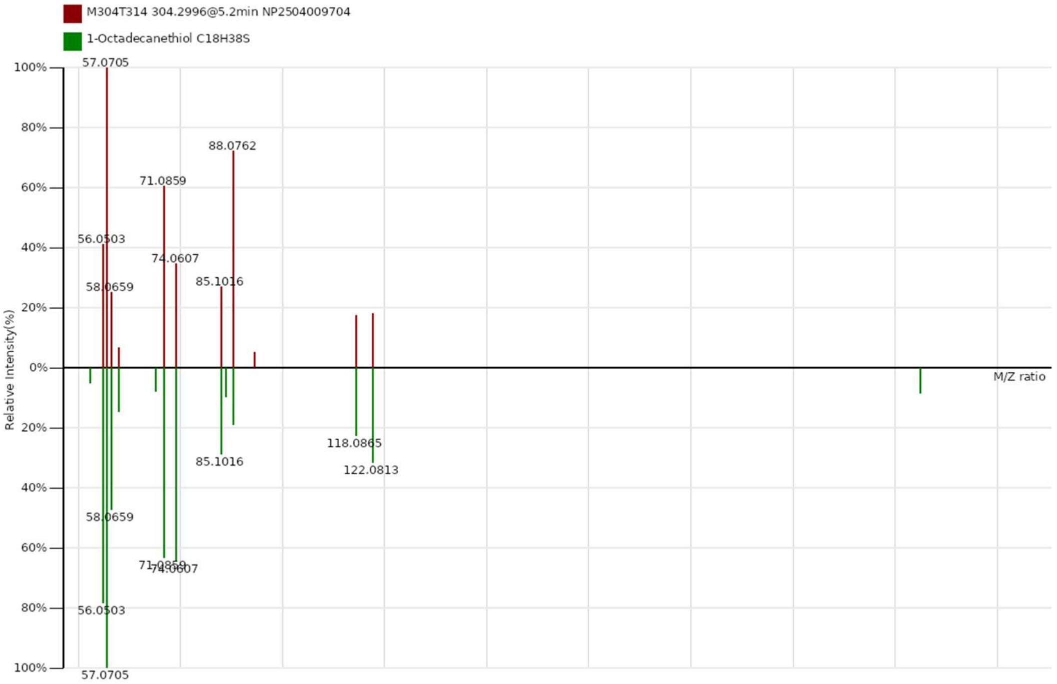
M304T314 304.2996@5.2min NP2504009704
1-Octadecanethiol C18H38S
Relative Intensity(%)
M/Z ratio
57.0705
88.0762
71.0859
56.0503
74.0607
85.1016
58.0659
118.0865
122.0813

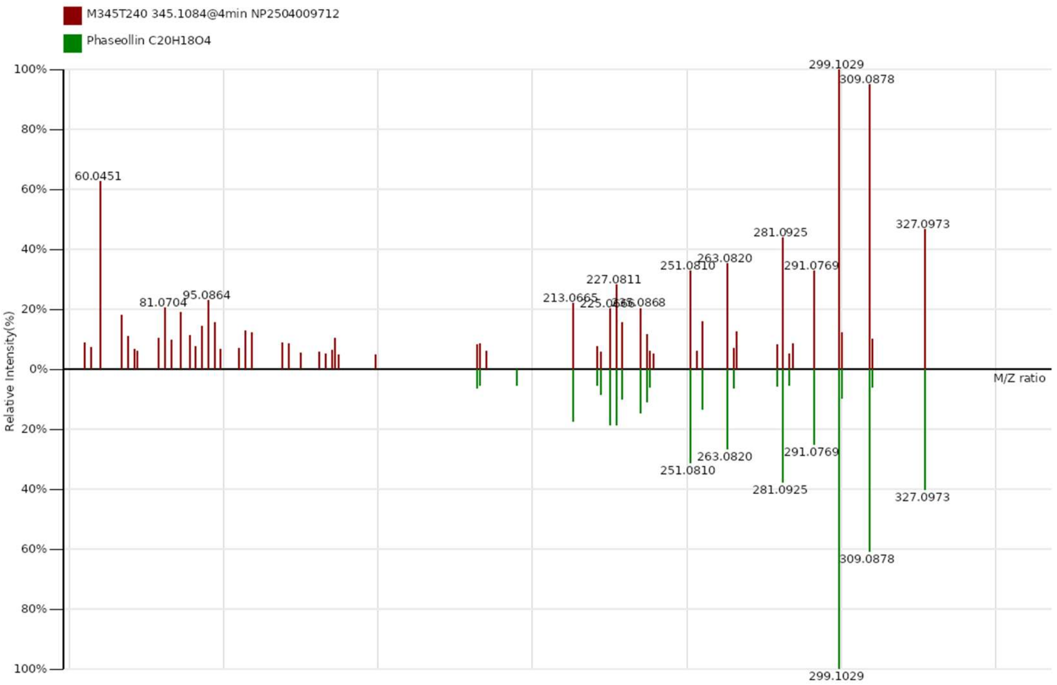

M345T240 345.1084@4min NP2504009712
Phaseollin C20H18O4
Relative Intensity(%)
M/Z ratio
60.0451
81.0704
95.0864
213.0665
225.0686
227.0811
235.0868
251.0810
263.0820
281.0925
291.0769
299.1029
309.0878
327.0973

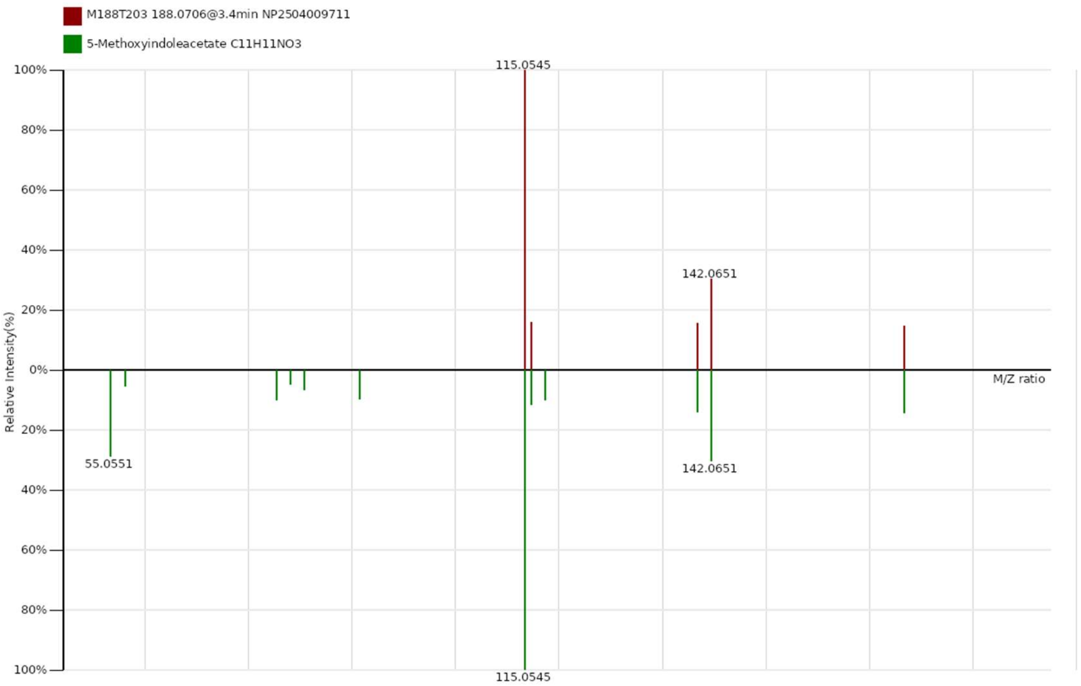

M188T203 188.0706@3.4min NP2504009711
5-Methoxyindoleacetate C11H11NO3
Relative Intensity(%)
M/Z ratio
115.0545
142.0651
55.0551

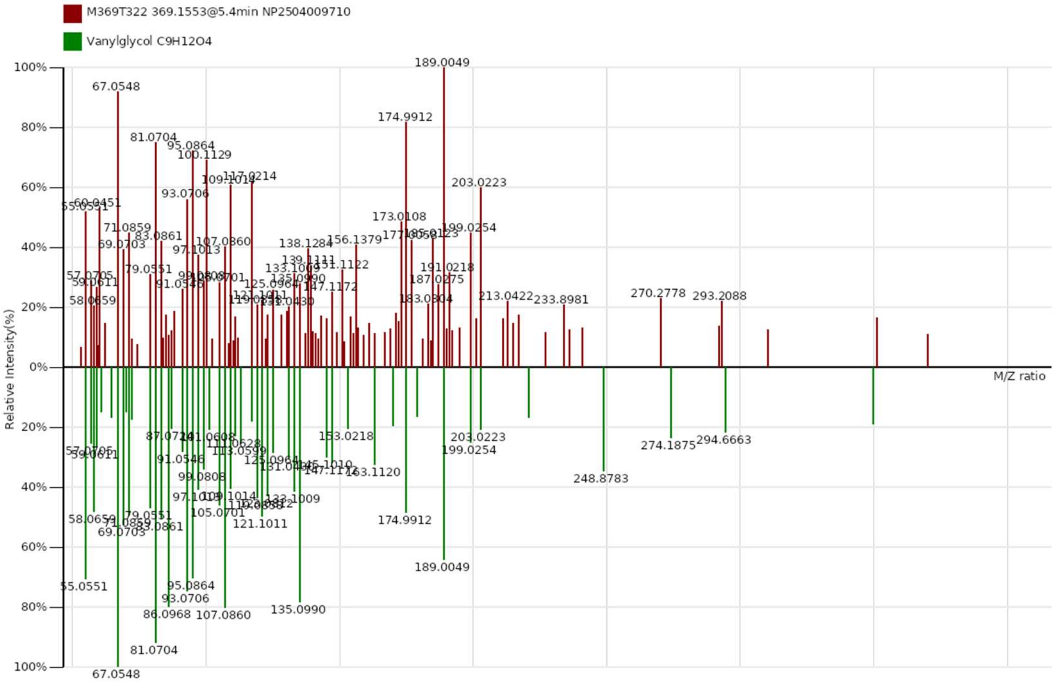
M369T322 369.1553@5.4min NP2504009710
Vanylglycol C9H12O4
Relative Intensity(%)
M/Z ratio
189.0049
67.0548
174.9912
81.0704
95.0864
203.0223
173.0108
199.0254
138.1284
156.1379
213.0422
233.8981
270.2778
293.2088
248.8783
274.1875
294.6663
135.0990
107.0860
86.0968
153.0218
163.1120

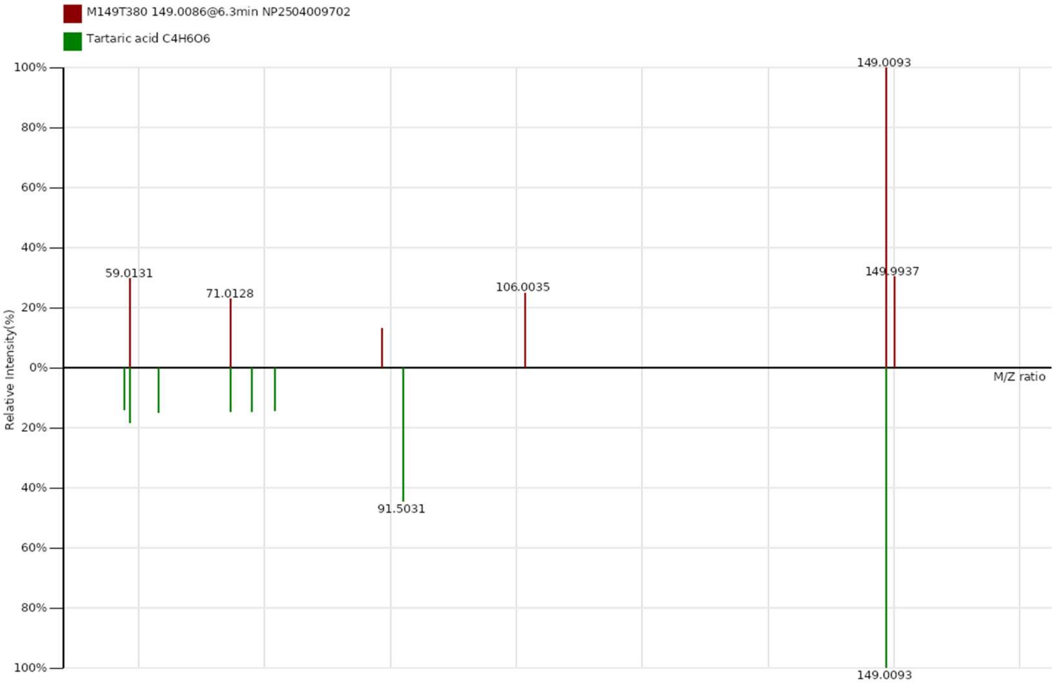
M149T380 149.0086@6.3min NP2504009702
Tartaric acid C4H6O6
Relative Intensity(%)
M/Z ratio
149.0093
59.0131
71.0128
106.0035
149.9937
91.5031

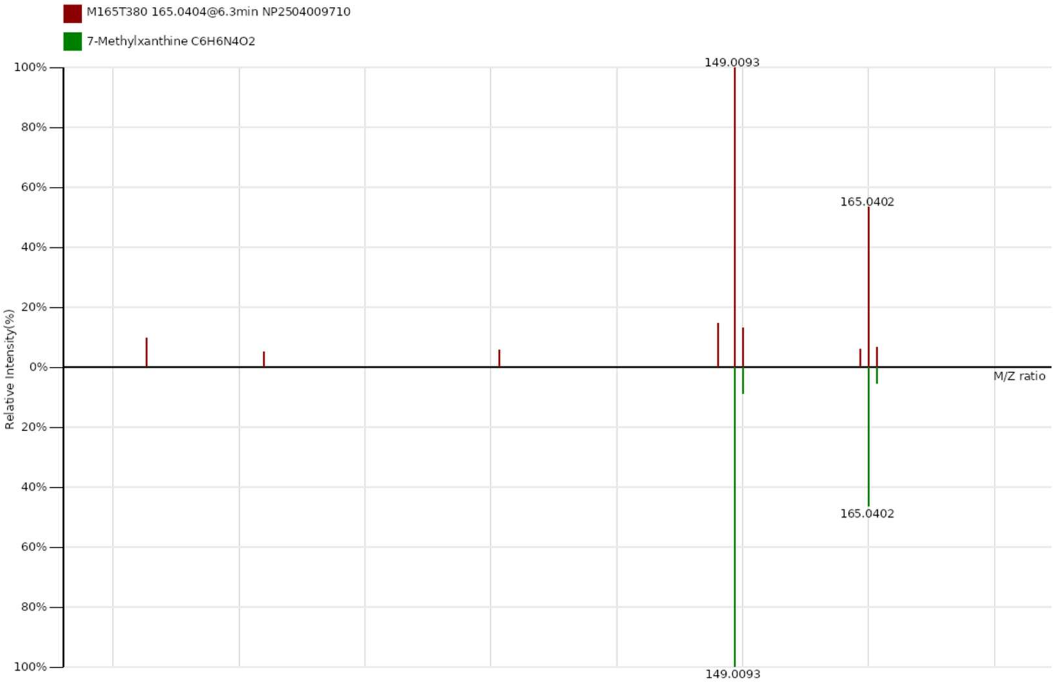

M165T380 165.0404@6.3min NP2504009710
7-Methylxanthine C6H6N4O2
100%
80%
60%
40%
20%
0%
20%
40%
60%
80%
100%
Relative Intensity(%)
M/Z ratio
149.0093
165.0402
165.0402
149.0093

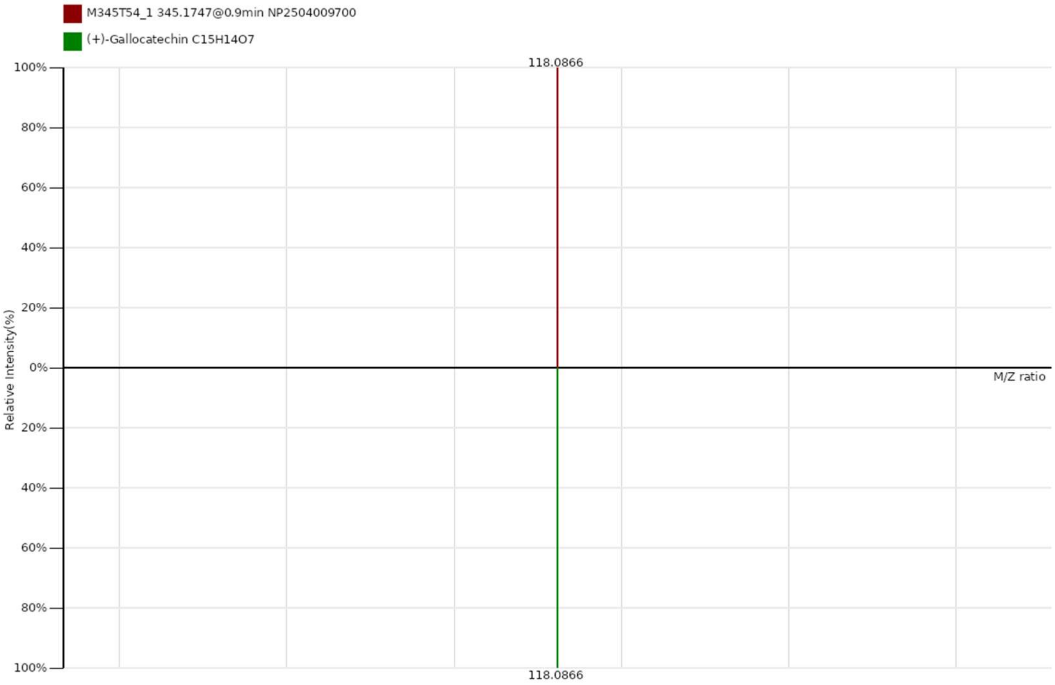

M345T54_1 345.1747@0.9min NP2504009700
(+)-Gallocatechin C15H14O7
100%
80%
60%
40%
20%
0%
20%
40%
60%
80%
100%
Relative Intensity(%)
M/Z ratio
118.0866
118.0866

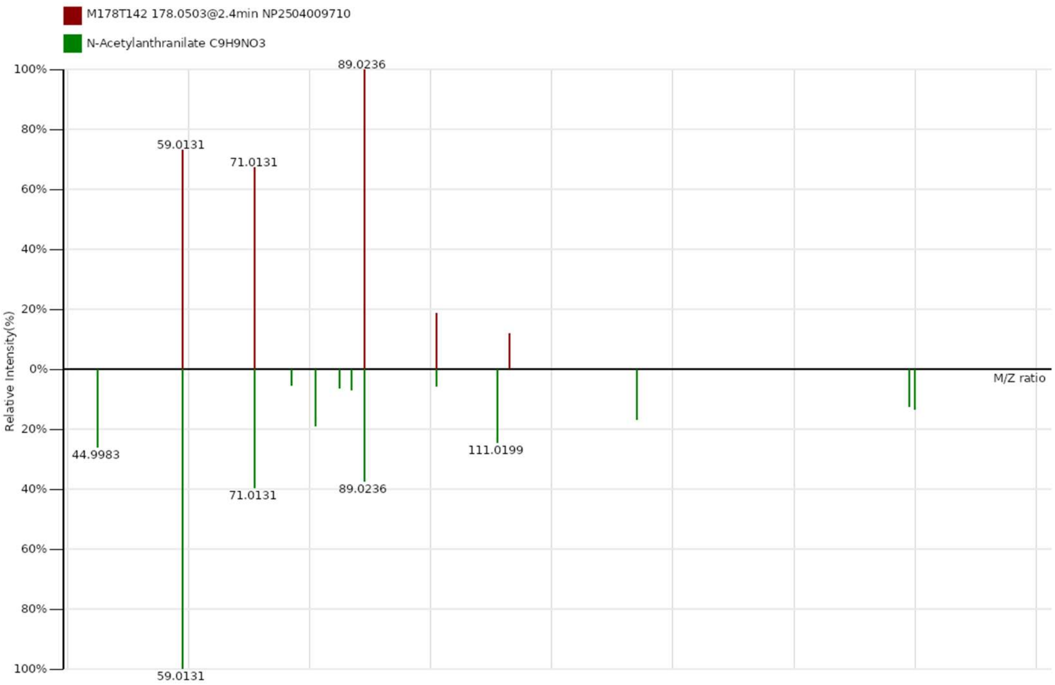

M178T142 178.0503@2.4min NP2504009710
N-Acetylanthranilate C9H9NO3
Relative Intensity(%)
M/Z ratio
100%
80%
60%
40%
20%
0%
89.0236
59.0131
71.0131
44.9983
111.0199

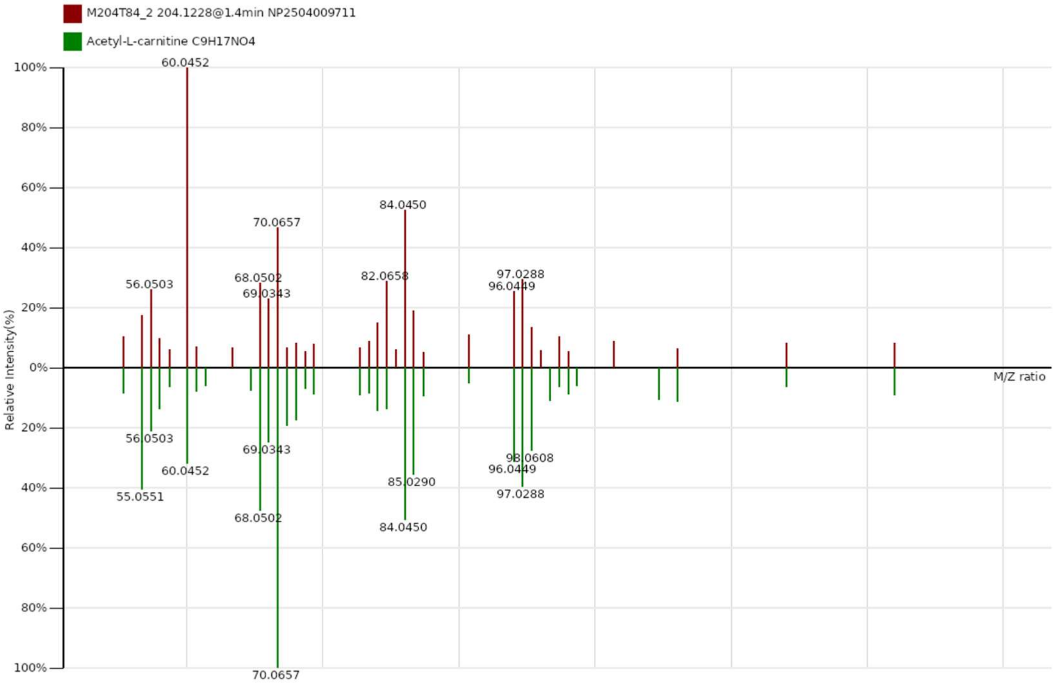

M204T84_2 204.1228@1.4min NP2504009711
Acetyl-L-carnitine C9H17NO4
Relative Intensity(%)
M/Z ratio
100%
80%
60%
40%
20%
0%
60.0452
70.0657
84.0450
56.0503
68.0502
69.0343
82.0658
97.0288
96.0449
55.0551
85.0290
98.0608

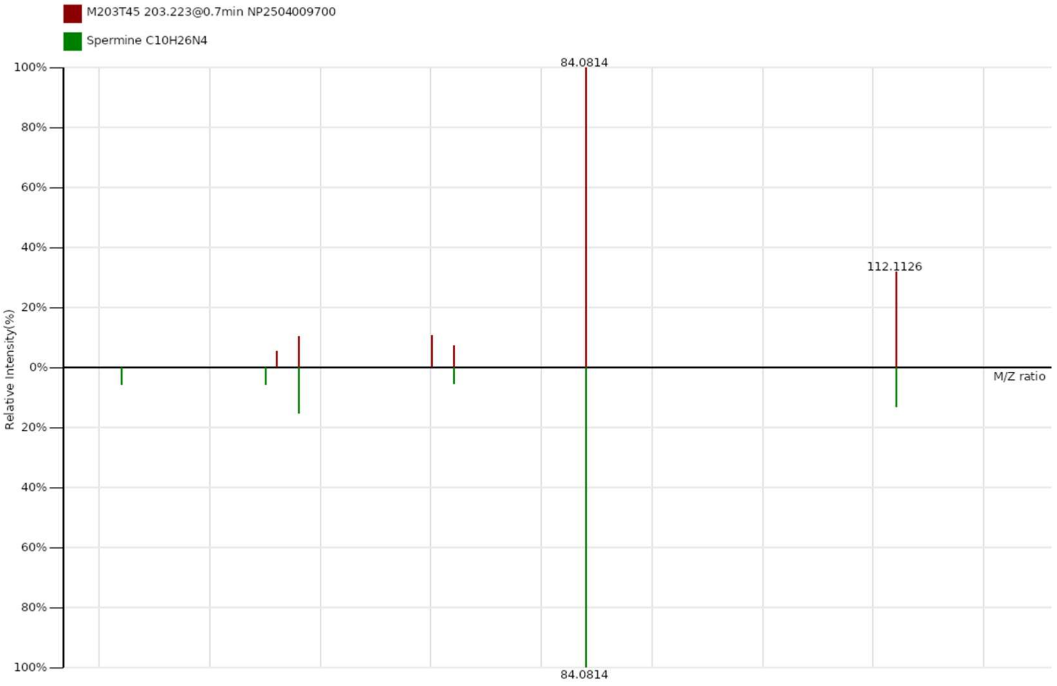
M203T45 203.223@0.7min NP2504009700
Spermine C10H26N4
100%
80%
60%
40%
20%
0%
20%
40%
60%
80%
100%
Relative Intensity(%)
84.0814
112.1126
M/Z ratio
84.0814

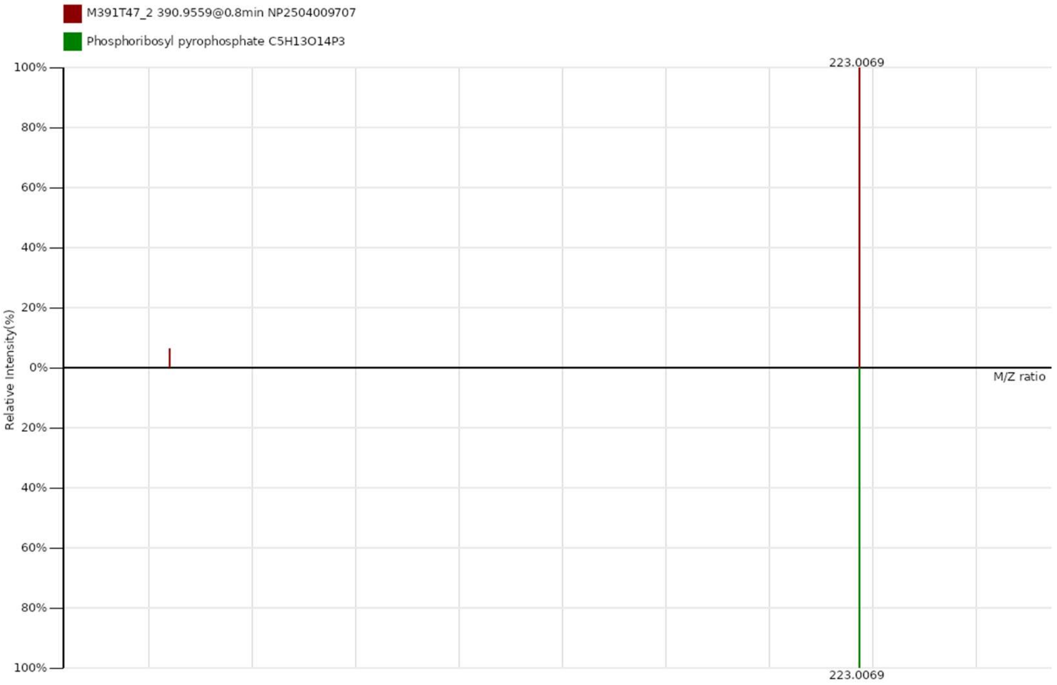
M391T47_2 390.9559@0.8min NP2504009707
Phosphoribosyl pyrophosphate C5H13O14P3
100%
80%
60%
40%
20%
0%
20%
40%
60%
80%
100%
Relative Intensity(%)
223.0069
M/Z ratio
223.0069

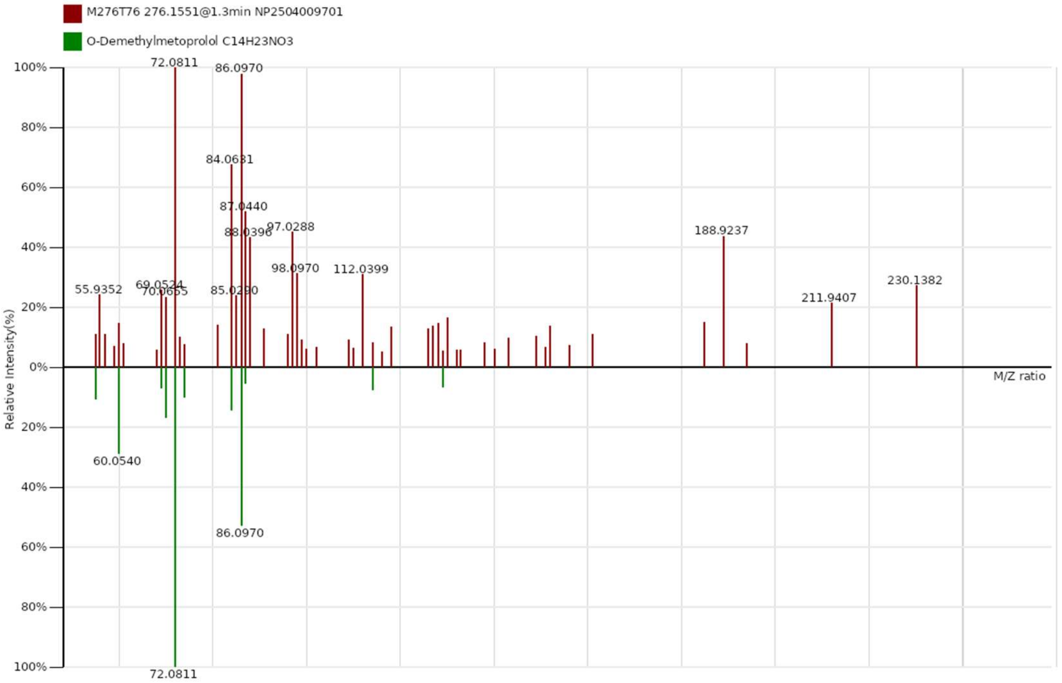

M276T76 276.1551@1.3min NP2504009701
O-Demethylmetoprolol C14H23NO3
72.0811
86.0970
84.0631
87.0440
88.0396
97.0288
188.9237
98.0970
112.0399
55.9352
69.0524
70.0655
85.0290
211.9407
230.1382
60.0540
86.0970
72.0811
Relative Intensity(%)
M/Z ratio

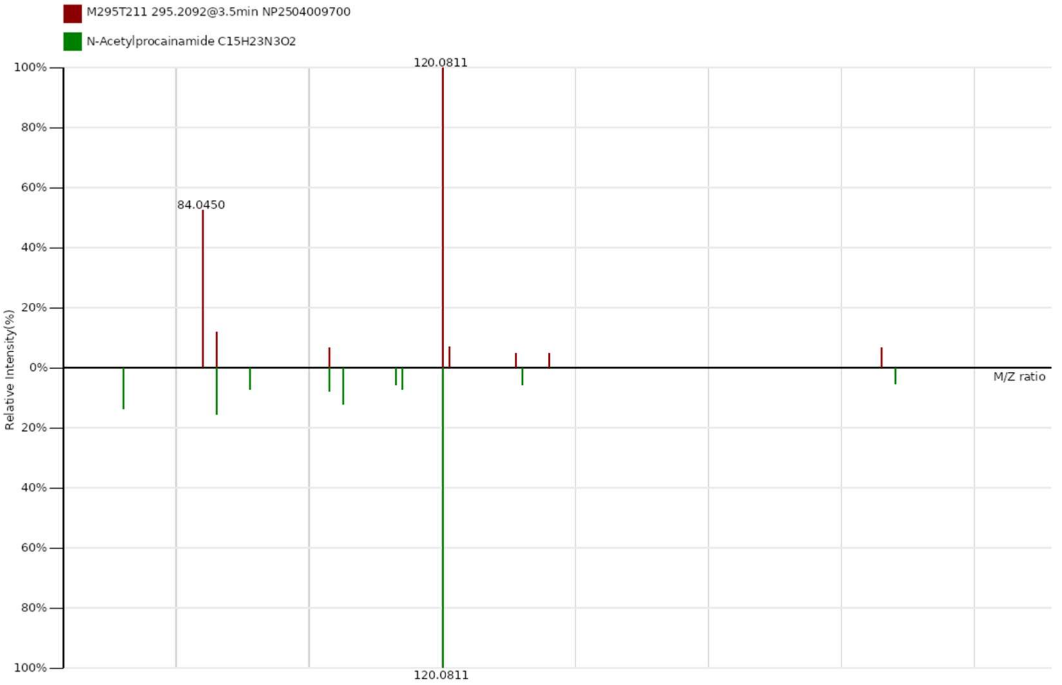

M295T211 295.2092@3.5min NP2504009700
N-Acetylprocainamide C15H23N3O2
120.0811
84.0450
120.0811
Relative Intensity(%)
M/Z ratio

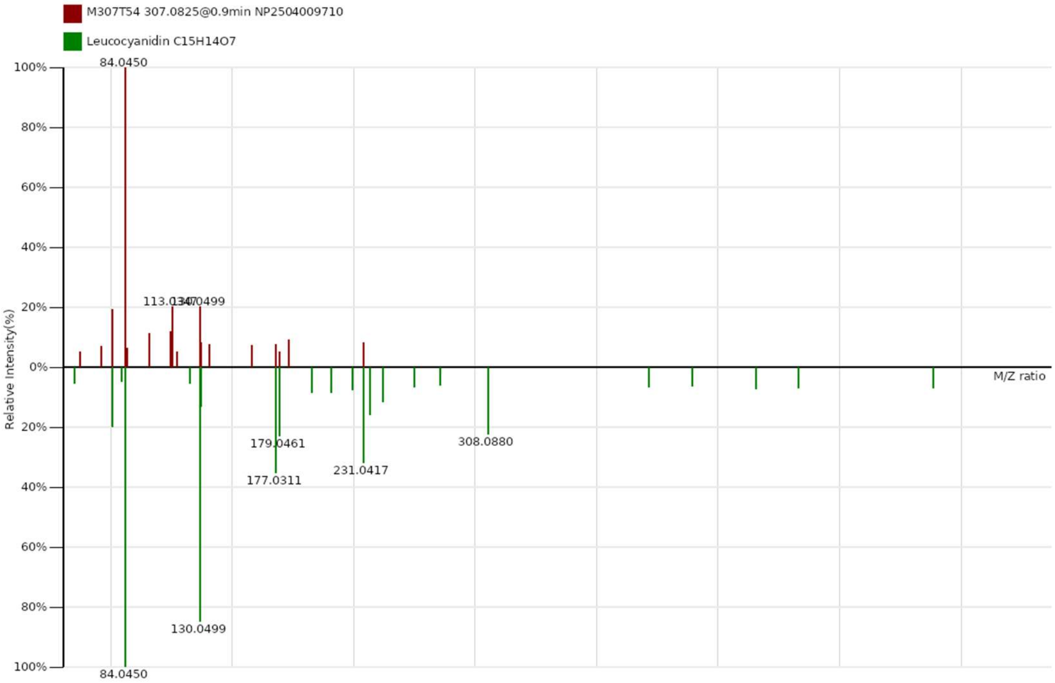
M307T54 307.0825@0.9min NP2504009710
Leucocyanidin C15H14O7
Relative Intensity(%)
M/Z ratio
84.0450
113.0347
130.0499
179.0461
177.0311
231.0417
308.0880
130.0499
84.0450

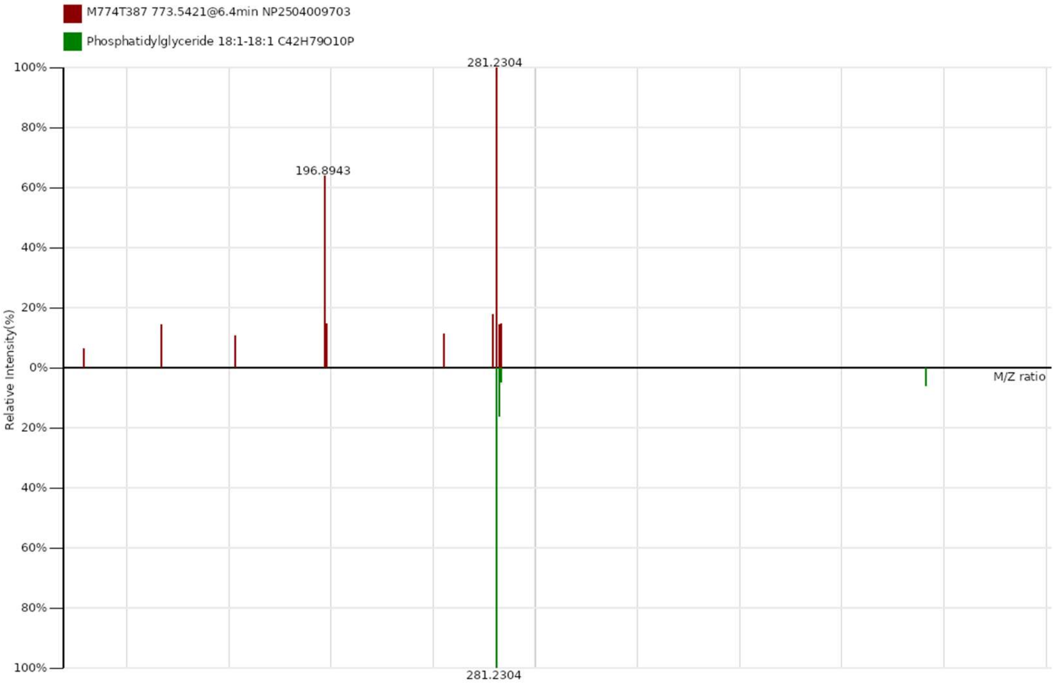
M774T387 773.5421@6.4min NP2504009703
Phosphatidylglyceride 18:1-18:1 C42H79O10P
Relative Intensity(%)
M/Z ratio
281.2304
196.8943
281.2304

Experimental fragments of the top 30 significant differential metabolites (DMs) from the comparison of B72H vs. BS48H.

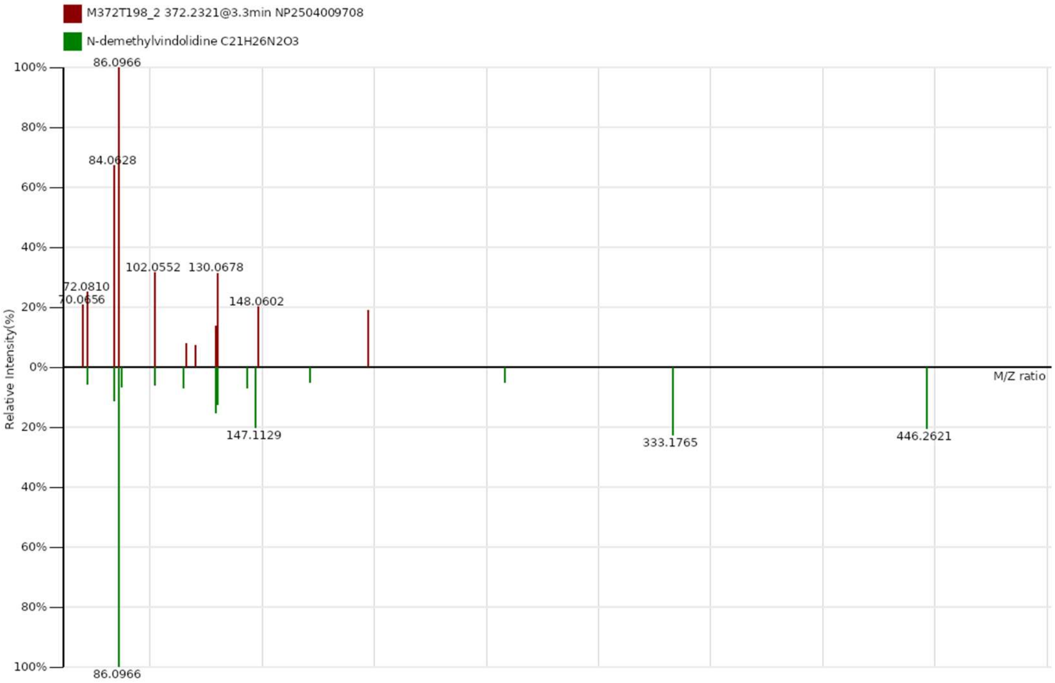

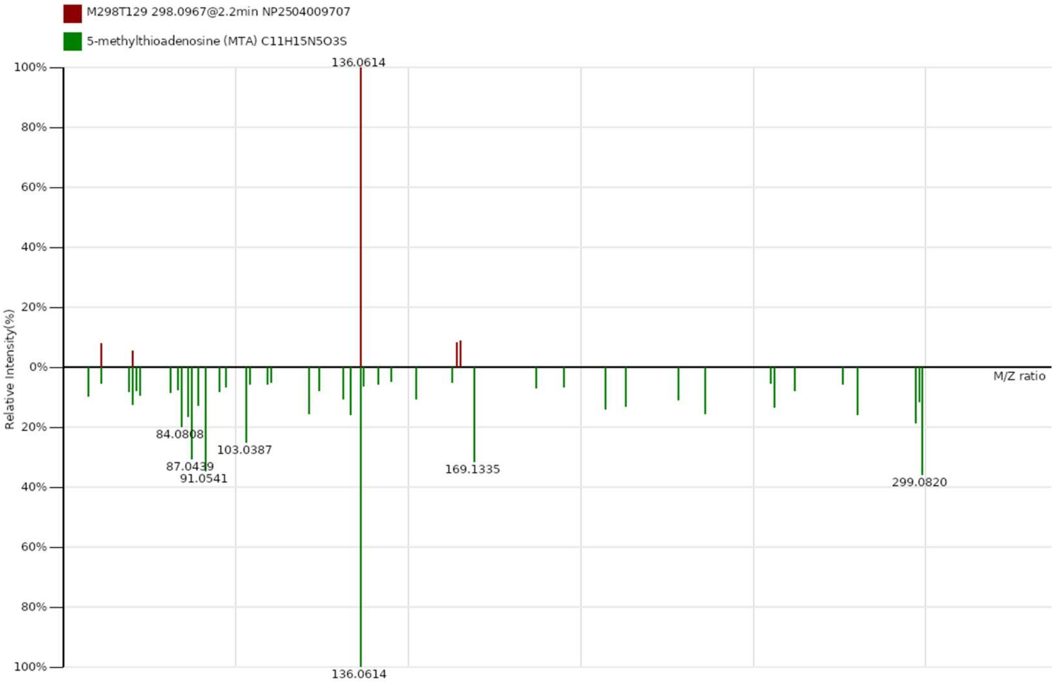

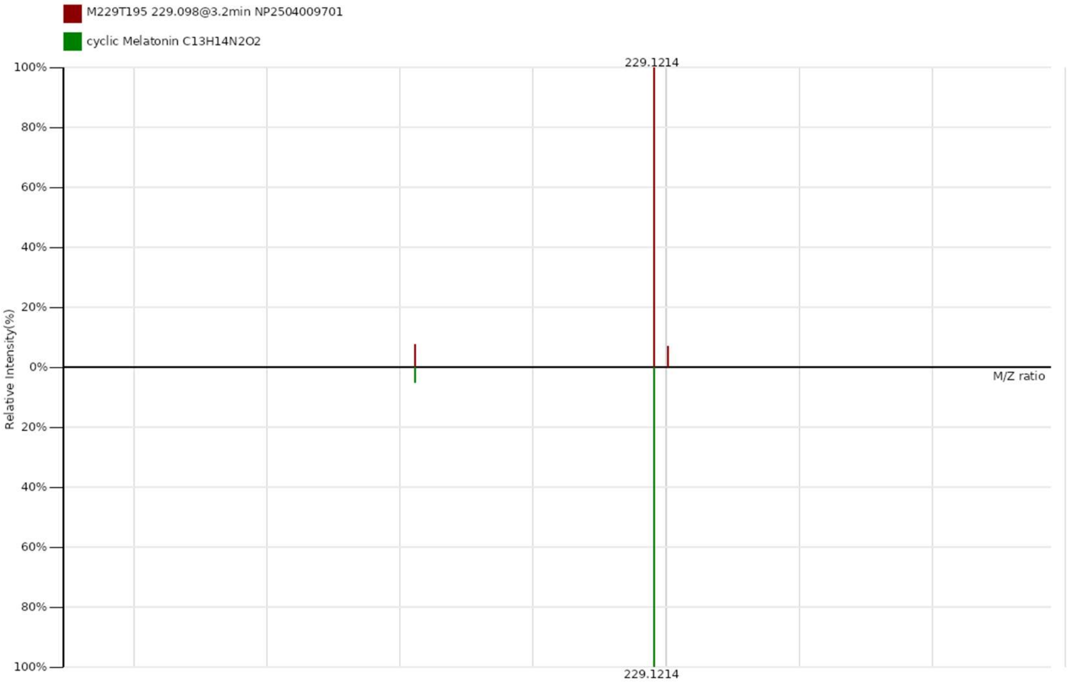

M229T195 229.098@3.2min NP2504009701
cyclic Melatonin C13H14N2O2
100%
80%
60%
40%
20%
0%
20%
40%
60%
80%
100%
Relative Intensity(%)
M/Z ratio
229.1214
229.1214

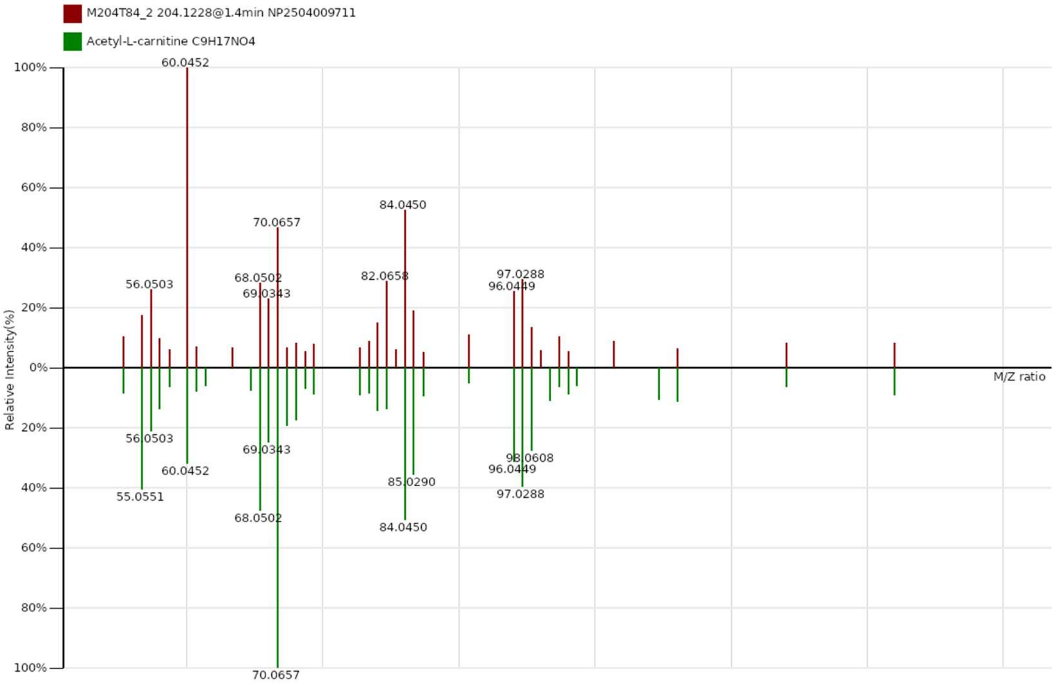

M204T84_2 204.1228@1.4min NP2504009711
Acetyl-L-carnitine C9H17NO4
100%
80%
60%
40%
20%
0%
20%
40%
60%
80%
100%
Relative Intensity(%)
M/Z ratio
60.0452
84.0450
70.0657
56.0503
68.0502
69.0343
82.0658
97.0288
96.0449
56.0503
60.0452
55.0551
69.0343
68.0502
85.0290
84.0450
98.0608
96.0449
97.0288
70.0657

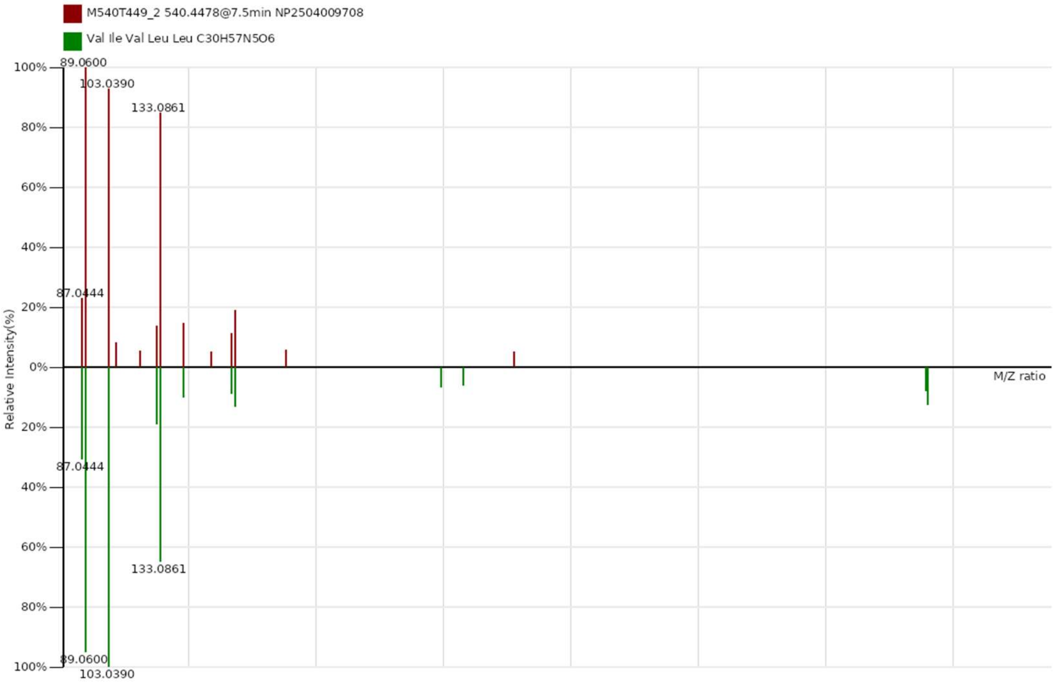
M540T449_2 540.4478@7.5min NP2504009708
Val Ile Val Leu Leu C30H57N5O6
Relative Intensity(%)
M/Z ratio
89.0600
103.0390
133.0861
87.0444
87.0444
133.0861
89.0600
103.0390

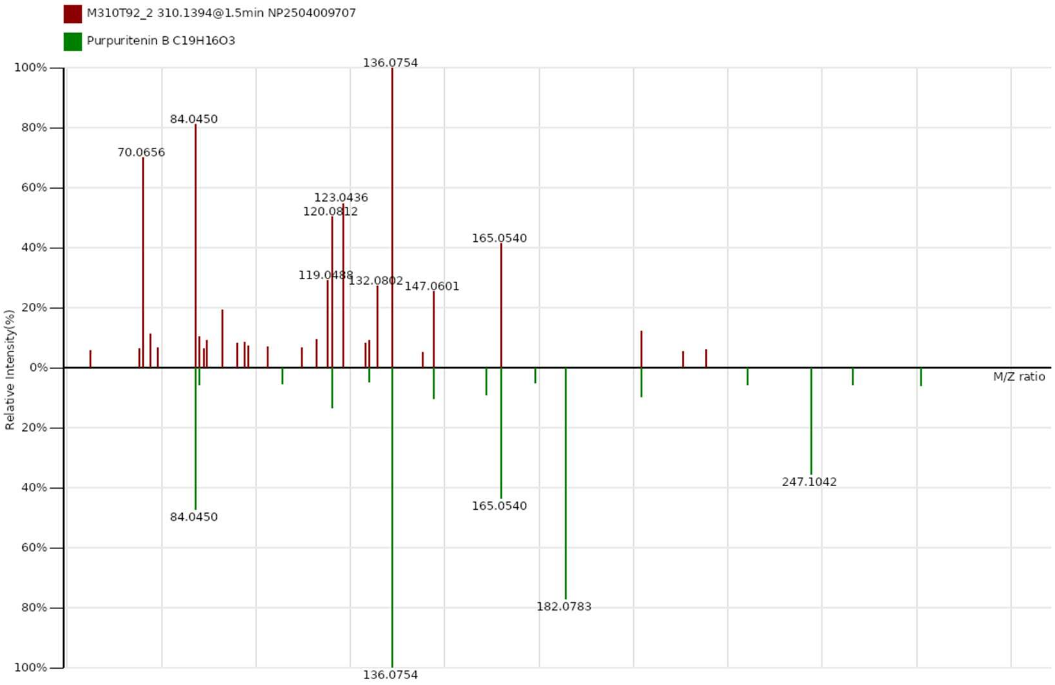
M310T92_2 310.1394@1.5min NP2504009707
Purpuritenin B C19H16O3
Relative Intensity(%)
M/Z ratio
136.0754
84.0450
70.0656
123.0436
120.0812
165.0540
119.0488
132.0802
147.0601
247.1042
84.0450
165.0540
182.0783
136.0754

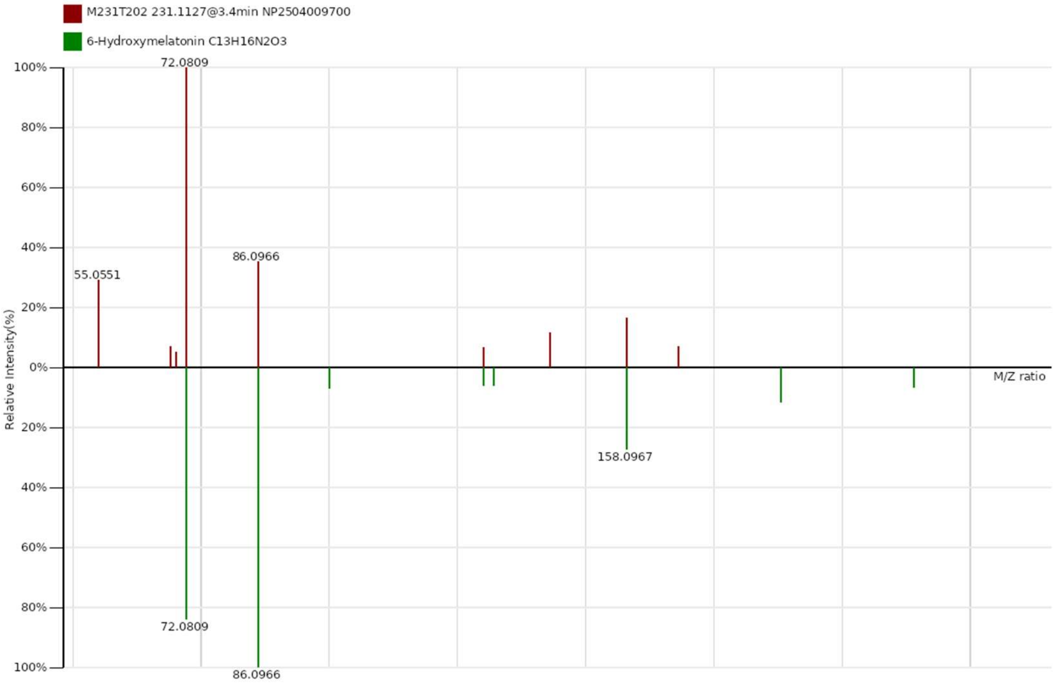
M231T202 231.1127@3.4min NP2504009700
6-Hydroxymelatonin C13H16N2O3
Relative Intensity(%)
M/Z ratio
100%
80%
60%
40%
20%
0%
72.0809
86.0966
55.0551
158.0967

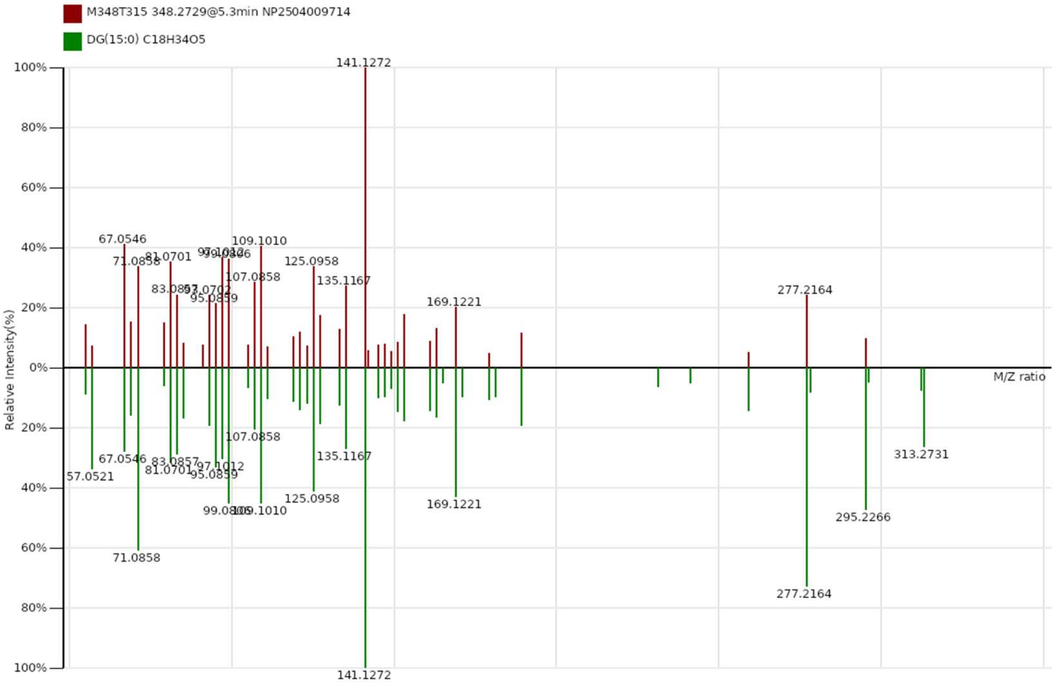
M348T315 348.2729@5.3min NP2504009714
DG(15:0) C18H34O5
Relative Intensity(%)
M/Z ratio
100%
80%
60%
40%
20%
0%
141.1272
67.0546
71.0858
81.0701
83.0857
95.0859
97.1012
99.0806
107.0858
109.1010
125.0958
135.1167
169.1221
277.2164
57.0521
295.2266
313.2731

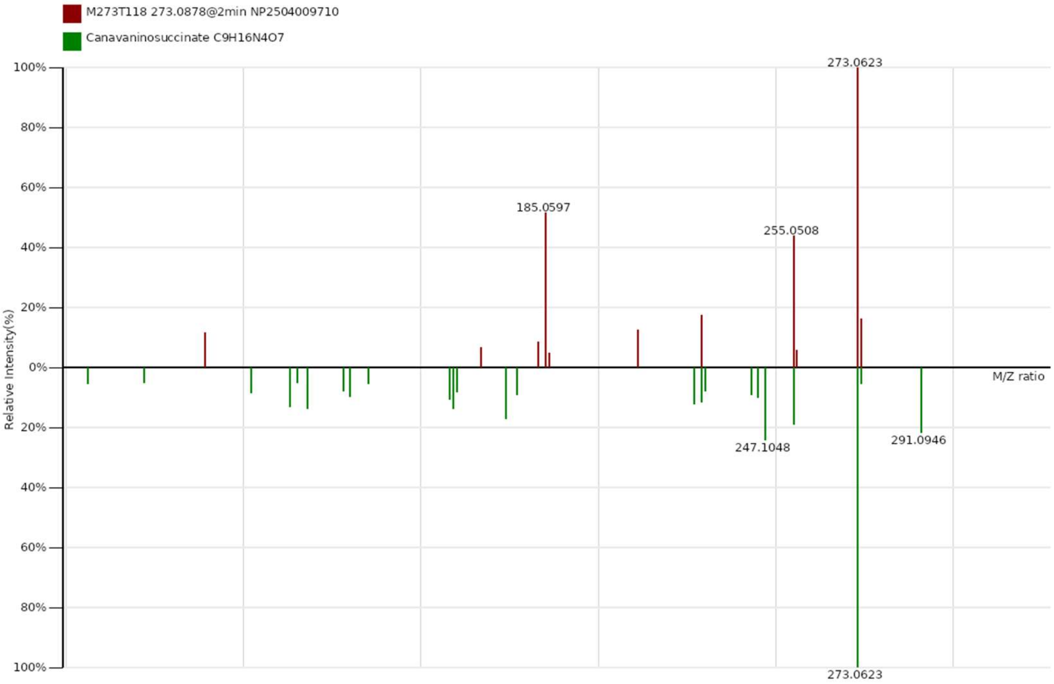

M273T118 273.0878@2min NP2504009710
Canavaninosuccinate C9H16N4O7
100%
80%
60%
40%
20%
0%
20%
40%
60%
80%
100%
Relative Intensity(%)
M/Z ratio
273.0623
185.0597
255.0508
247.1048
291.0946
273.0623

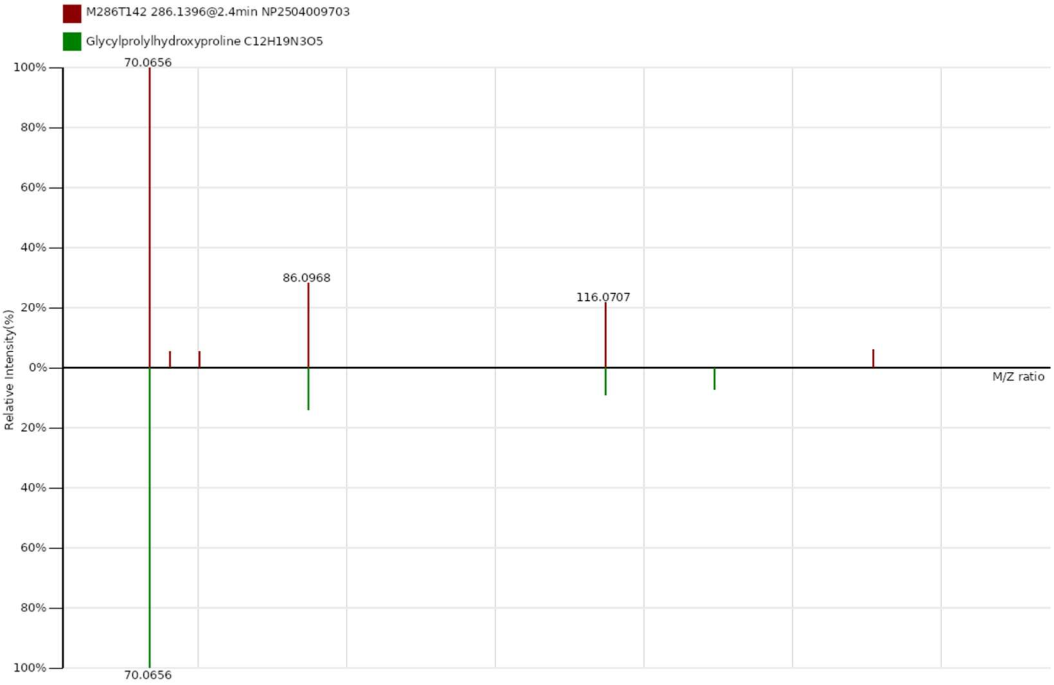

M286T142 286.1396@2.4min NP2504009703
Glycylprolylhydroxyproline C12H19N3O5
100%
80%
60%
40%
20%
0%
20%
40%
60%
80%
100%
Relative Intensity(%)
M/Z ratio
70.0656
86.0968
116.0707
70.0656

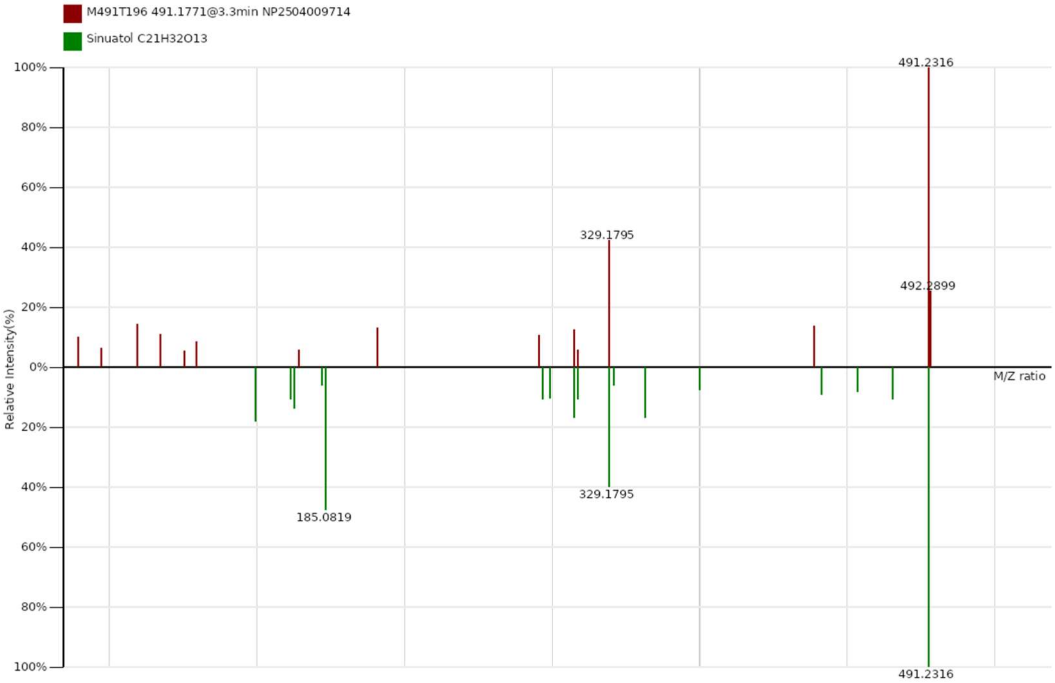
M491T196 491.1771@3.3min NP2504009714
Sinuatol C21H32O13
Relative Intensity(%)
M/Z ratio
100%
80%
60%
40%
20%
0%
491.2316
329.1795
492.2899
185.0819
329.1795
491.2316

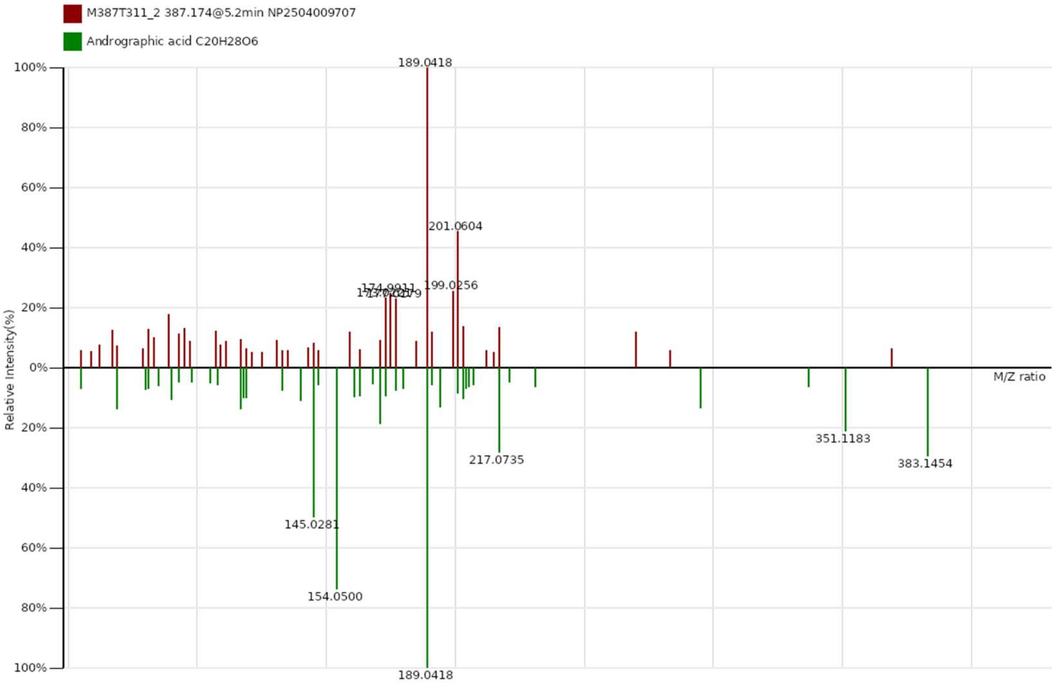
M387T311_2 387.174@5.2min NP2504009707
Andrographic acid C20H28O6
Relative Intensity(%)
M/Z ratio
100%
80%
60%
40%
20%
0%
189.0418
201.0604
174.0911
199.0256
145.0281
154.0500
217.0735
351.1183
383.1454
189.0418

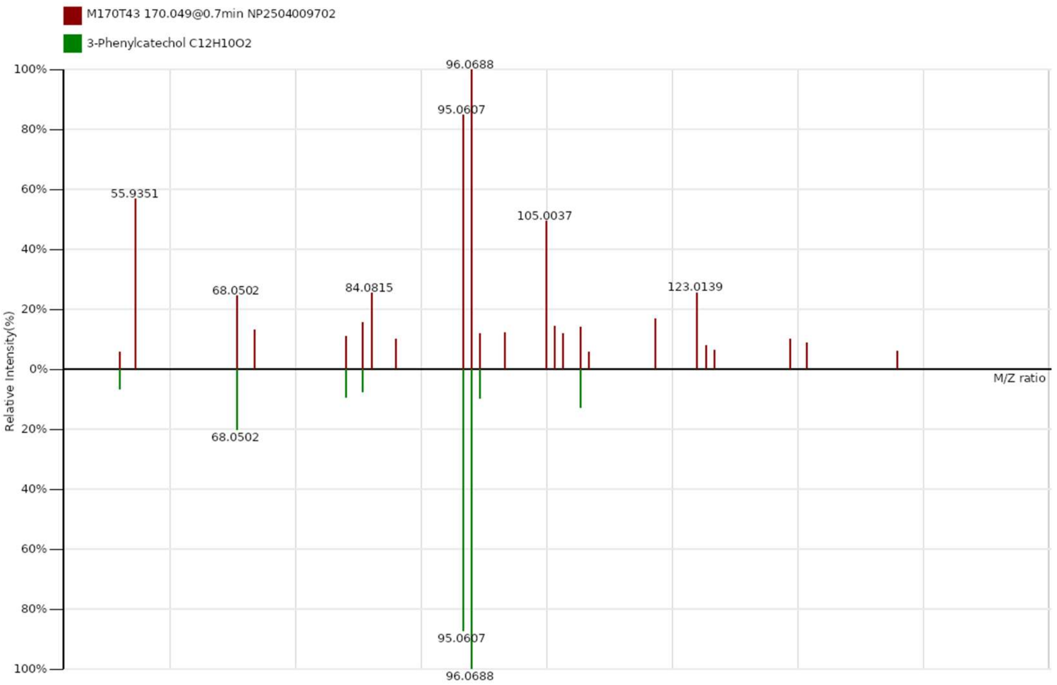

M170T43 170.049@0.7min NP2504009702
3-Phenylcatechol C12H10O2
100%
80%
60%
40%
20%
0%
20%
40%
60%
80%
100%
Relative Intensity(%)
M/Z ratio
96.0688
95.0607
55.9351
105.0037
68.0502
84.0815
123.0139
68.0502
95.0607
96.0688

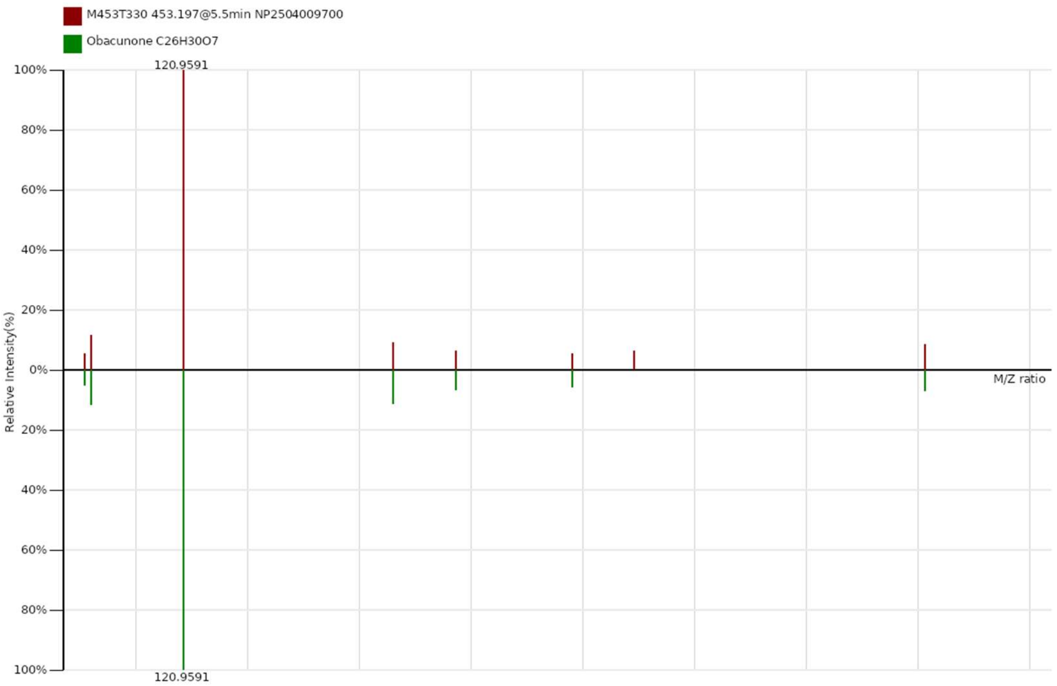

M453T330 453.197@5.5min NP2504009700
Obacunone C26H30O7
100%
80%
60%
40%
20%
0%
20%
40%
60%
80%
100%
Relative Intensity(%)
M/Z ratio
120.9591
120.9591

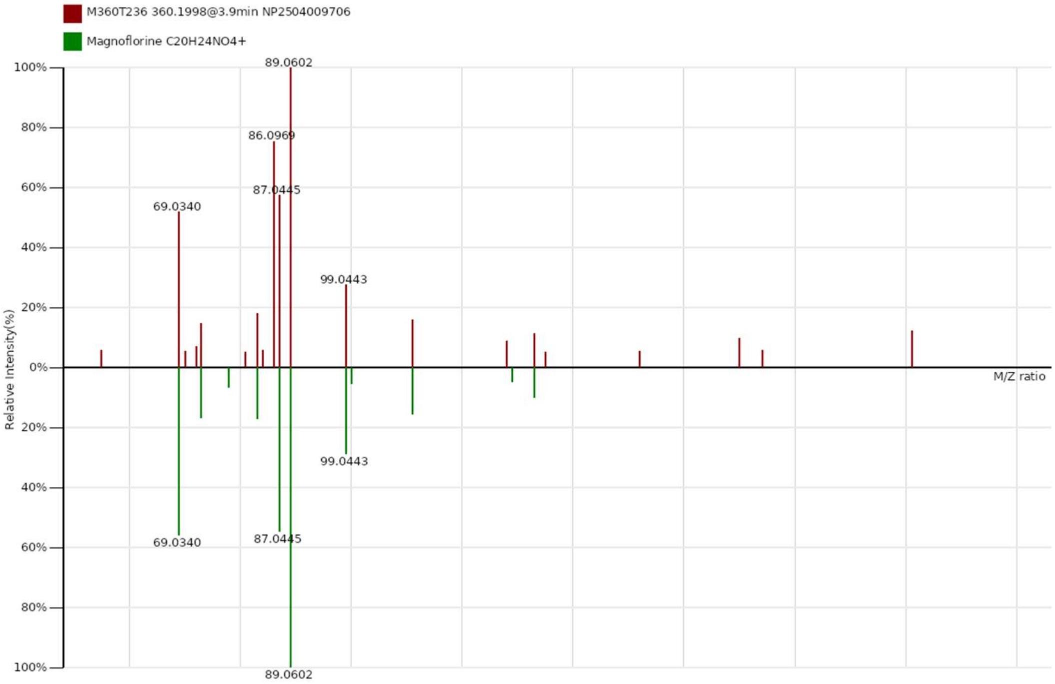
M360T236 360.1998@3.9min NP2504009706
Magnoflorine C20H24NO4+
89.0602
86.0969
87.0445
69.0340
99.0443
Relative Intensity(%)
M/Z ratio
100%
80%
60%
40%
20%
0%

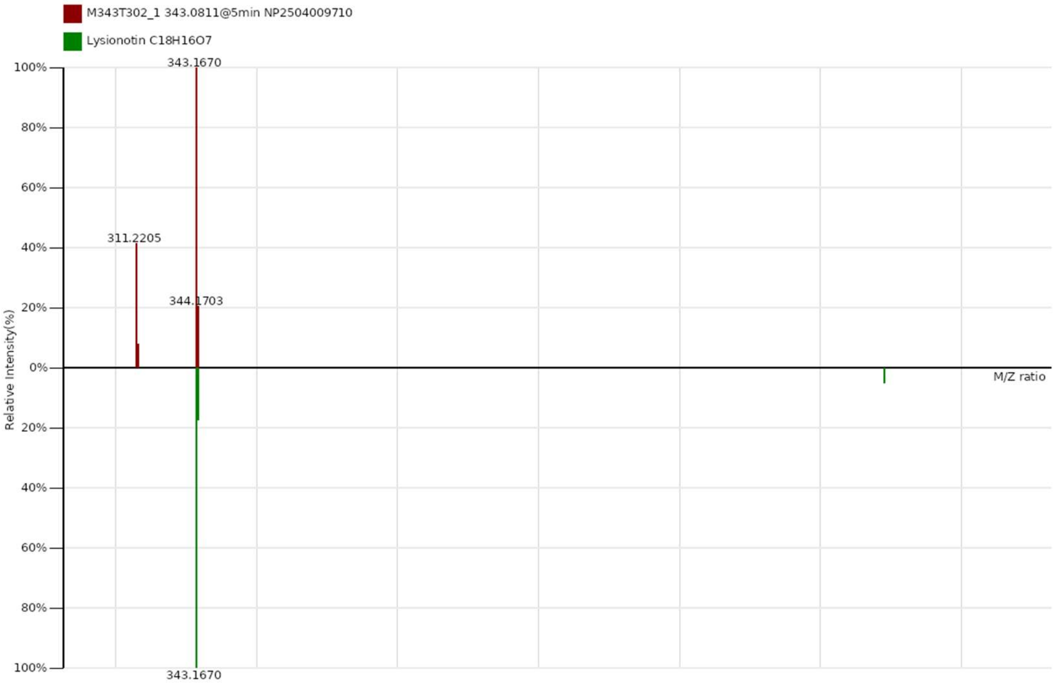
M343T302_1 343.0811@5min NP2504009710
Lysionotin C18H16O7
343.1670
311.2205
344.1703
Relative Intensity(%)
M/Z ratio
100%
80%
60%
40%
20%
0%

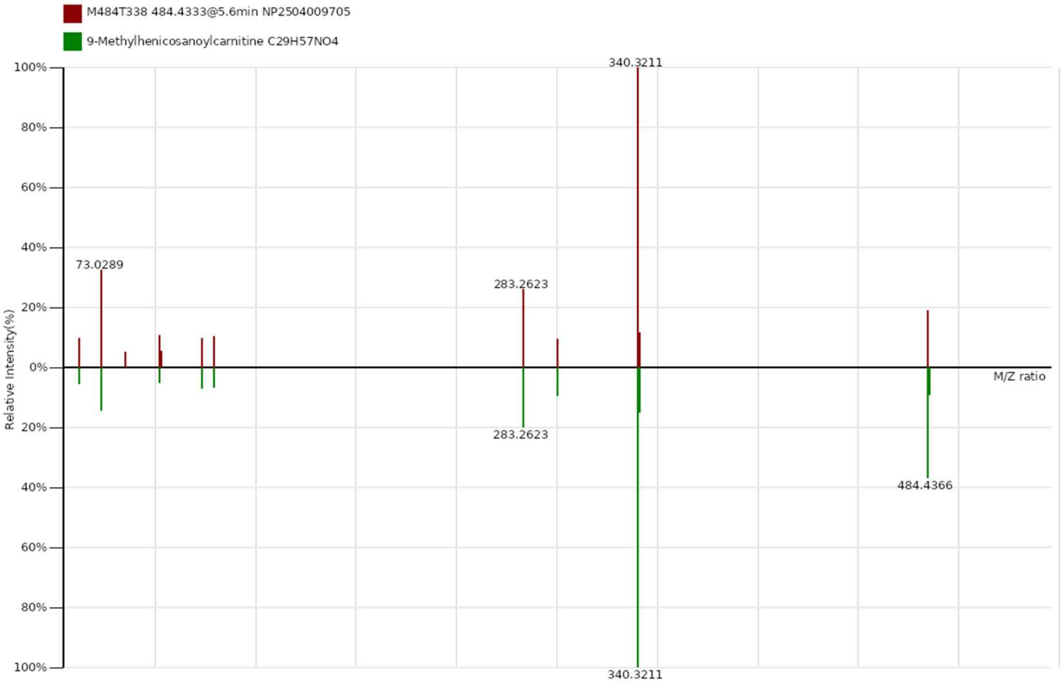

M484T338 484.4333@5.6min NP2504009705
9-Methylhenicosanoylcarnitine C29H57NO4
Relative Intensity(%)
M/Z ratio
100%
80%
60%
40%
20%
0%
73.0289
283.2623
340.3211
484.4366

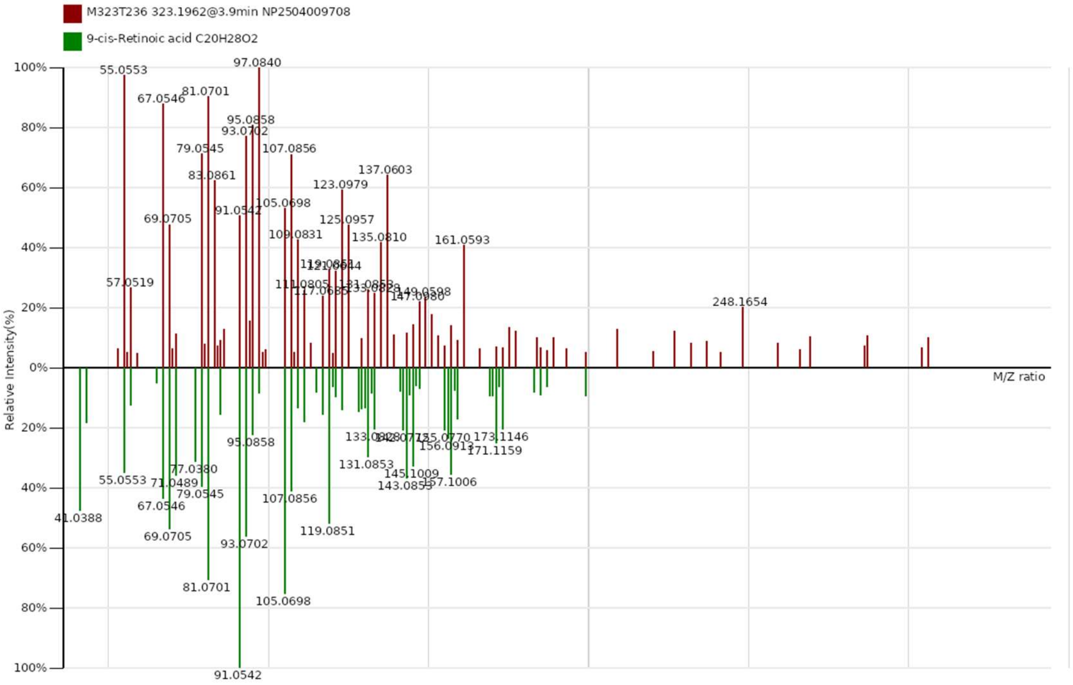

M323T236 323.1962@3.9min NP2504009708
9-cis-Retinoic acid C20H28O2
Relative Intensity(%)
M/Z ratio
100%
80%
60%
40%
20%
0%
55.0553
97.0840
67.0546
81.0701
95.0858
93.0702
79.0545
107.0856
83.0861
137.0603
123.0979
105.0698
69.0705
91.0542
125.0957
109.0831
135.0810
161.0593
57.0519
111.0805
117.0685
147.0980
248.1654
41.0388
77.0380
71.0489
119.0851
131.0853
145.1009
143.0855
157.1006
173.1146
171.1159
156.0913

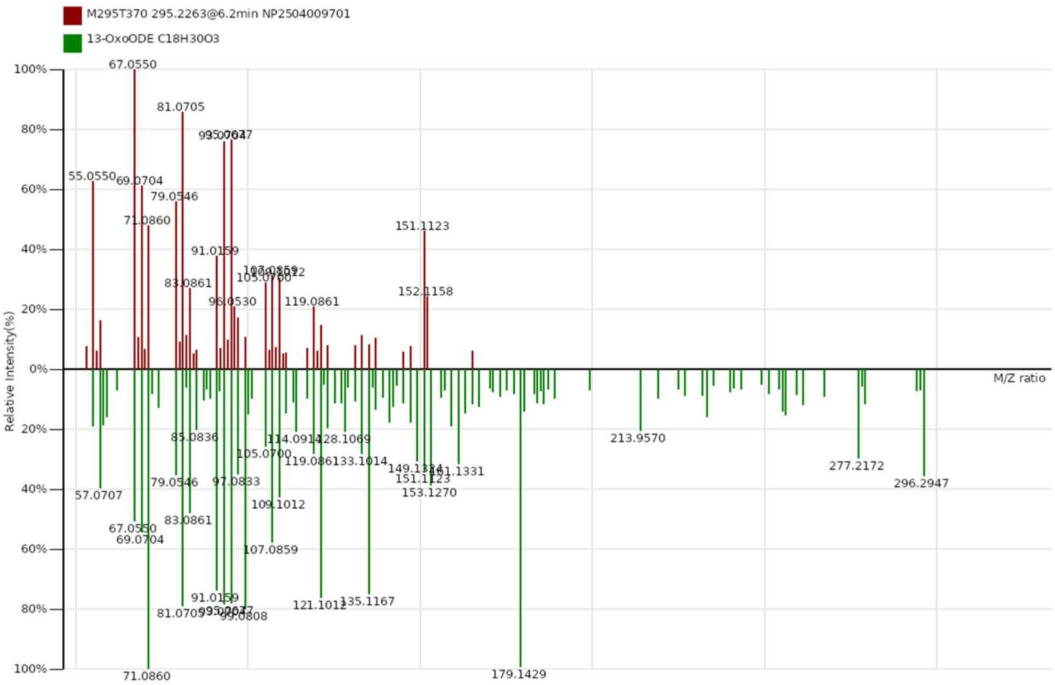
M295T370 295.2263@6.2min NP2504009701
13-OxoODE C18H30O3
Relative Intensity(%)
M/Z ratio
67.0550
81.0705
55.0550
69.0704
79.0546
71.0860
91.0159
83.0861
96.0530
119.0861
151.1123
152.1158
85.0836
114.0914
128.1069
105.0700
119.0861
133.1014
149.1324
151.1123
161.1331
153.1270
97.0833
109.1012
107.0859
57.0707
213.9570
277.2172
296.2947
121.1012
135.1167
99.0808
179.1429
71.0860

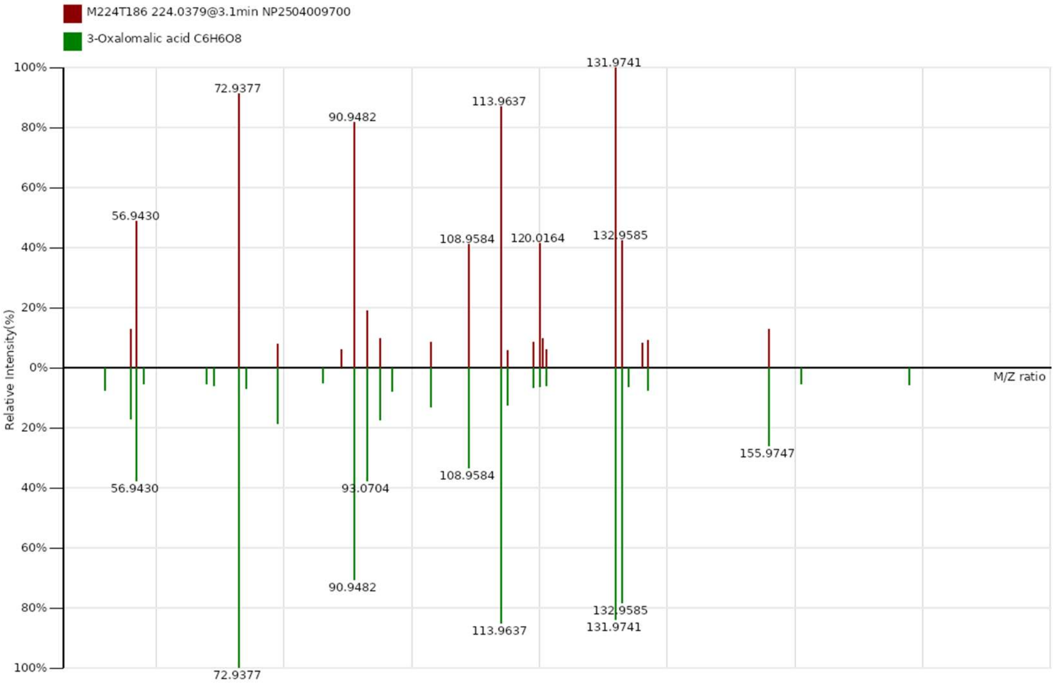
M224T186 224.0379@3.1min NP2504009700
3-Oxalomalic acid C6H6O8
Relative Intensity(%)
M/Z ratio
131.9741
72.9377
113.9637
90.9482
56.9430
108.9584
120.0164
132.9585
155.9747
56.9430
93.0704
108.9584
90.9482
132.9585
113.9637
131.9741
72.9377

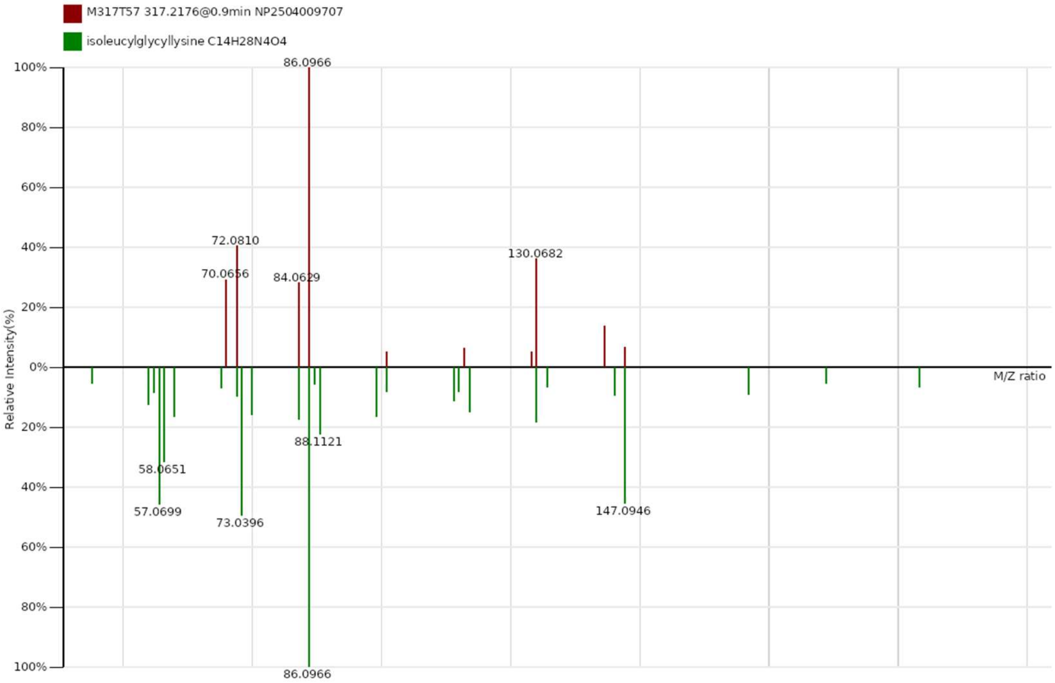
M317T57 317.2176@0.9min NP2504009707
isoleucylglycyllysine C14H28N4O4
Relative Intensity(%)
M/Z ratio
100%
80%
60%
40%
20%
0%
86.0966
72.0810
70.0656
84.0629
130.0682
88.1121
58.0651
57.0699
73.0396
147.0946
86.0966

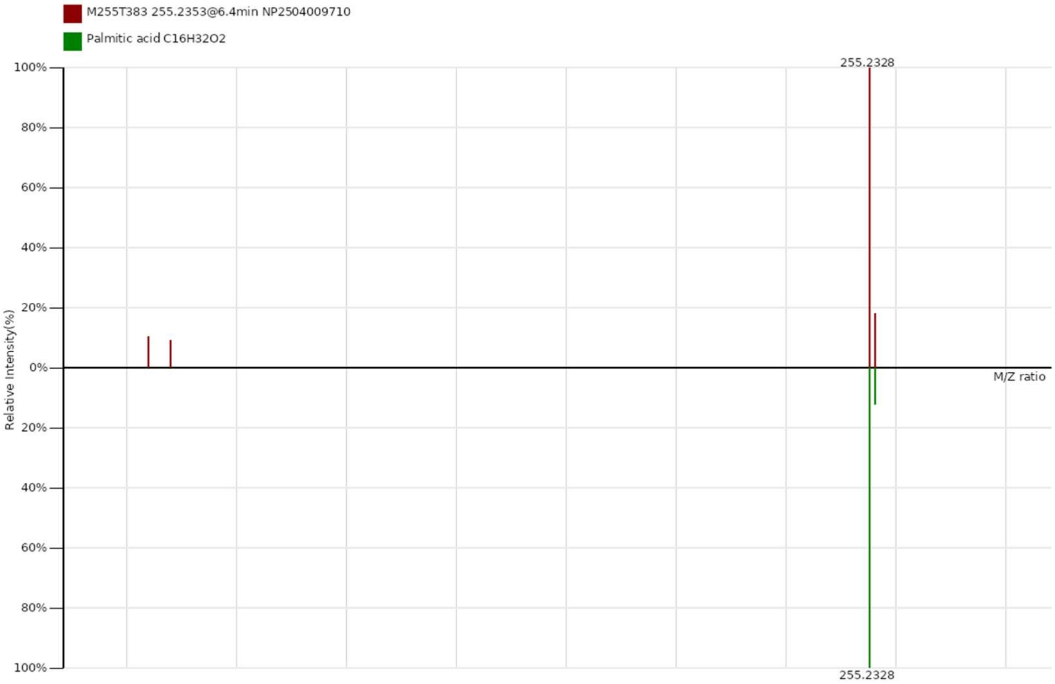
M255T383 255.2353@6.4min NP2504009710
Palmitic acid C16H32O2
Relative Intensity(%)
M/Z ratio
100%
80%
60%
40%
20%
0%
255.2328
255.2328

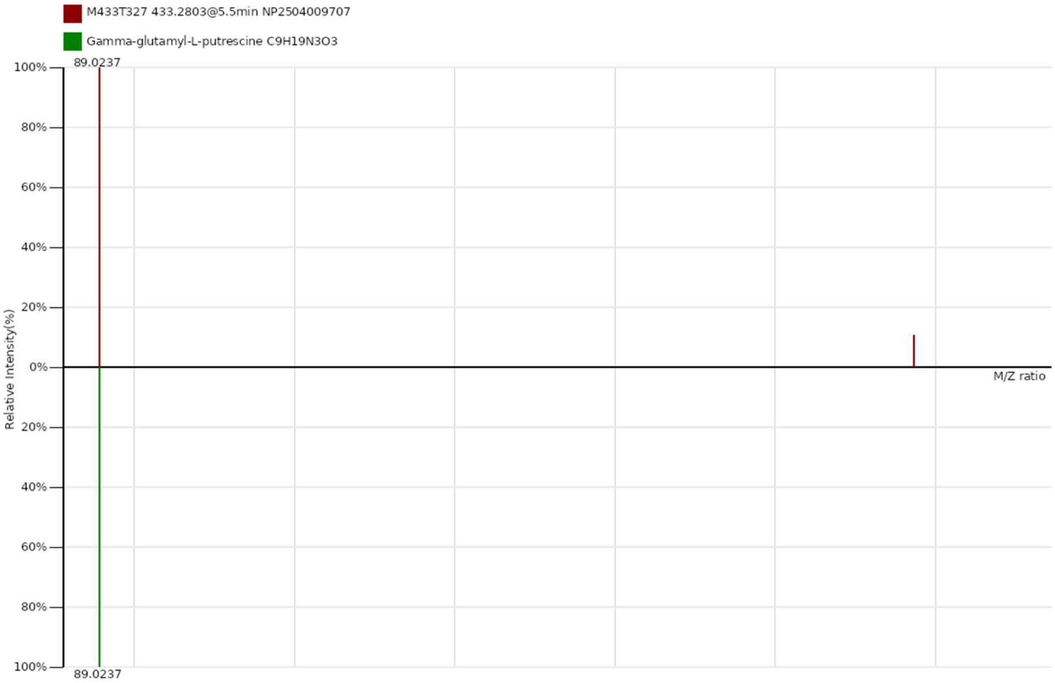
M433T327 433.2803@5.5min NP2504009707
Gamma-glutamyl-L-putrescine C9H19N3O3
89.0237
Relative Intensity(%)
M/Z ratio
89.0237

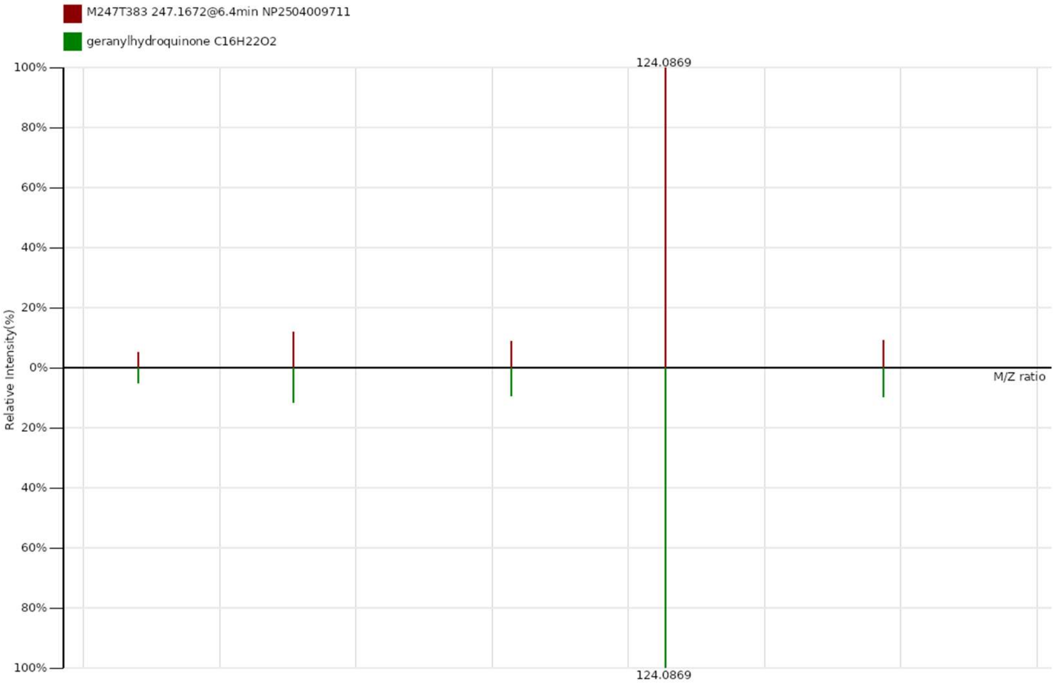
M247T383 247.1672@6.4min NP2504009711
geranylhydroquinone C16H22O2
124.0869
Relative Intensity(%)
M/Z ratio
124.0869

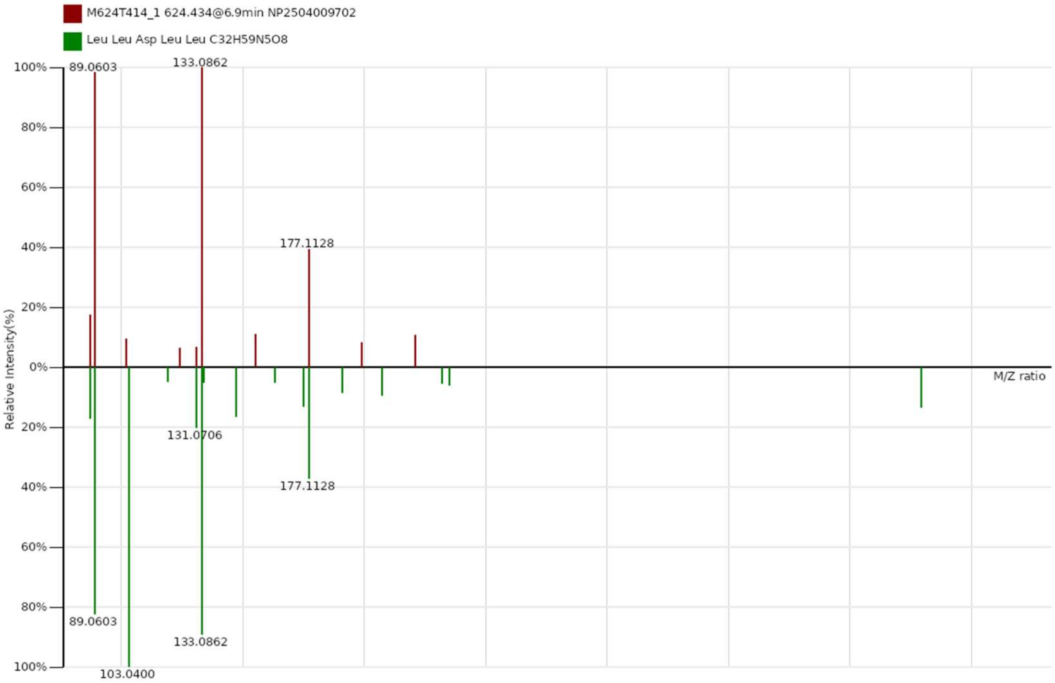

M624T414_1 624.434@6.9min NP2504009702
Leu Leu Asp Leu Leu C32H59N5O8
100%
80%
60%
40%
20%
0%
20%
40%
60%
80%
100%
Relative Intensity(%)
M/Z ratio
89.0603
133.0862
177.1128
131.0706
177.1128
89.0603
133.0862
103.0400

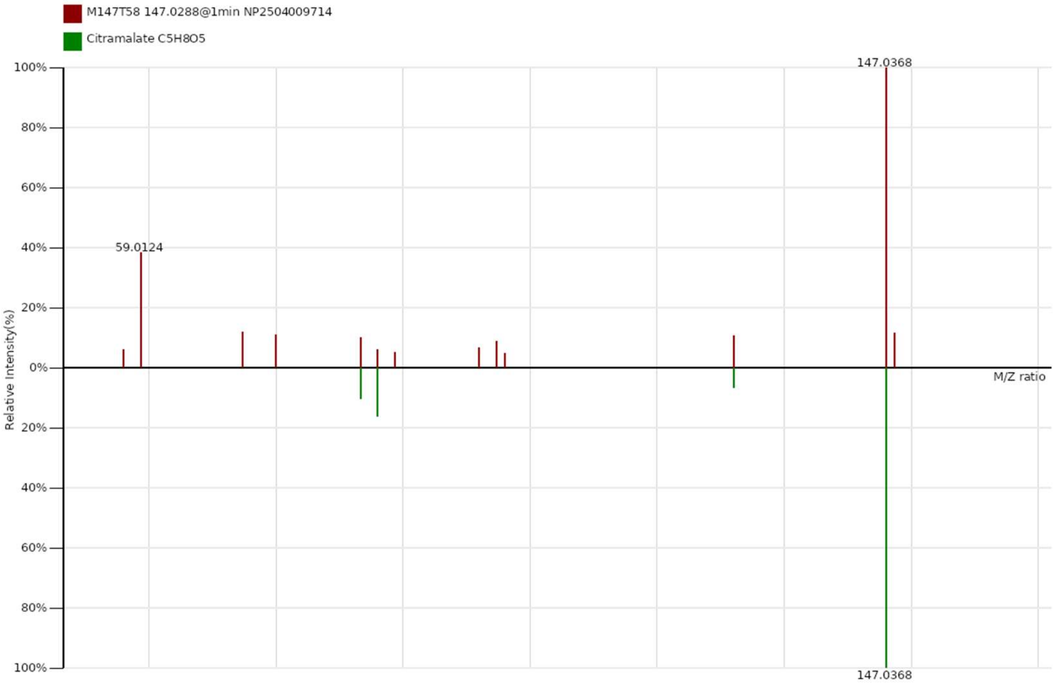

M147T58 147.0288@1min NP2504009714
Citramalate C5H8O5
100%
80%
60%
40%
20%
0%
20%
40%
60%
80%
100%
Relative Intensity(%)
M/Z ratio
147.0368
59.0124
147.0368

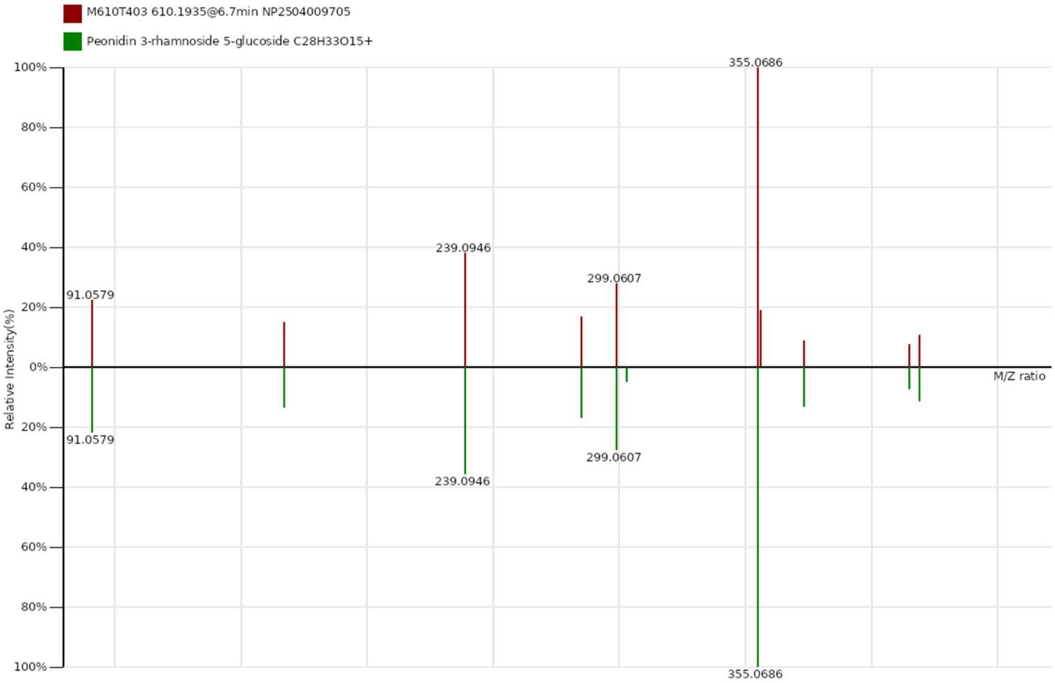
M610T403 610.1935@6.7min NP2504009705
Peonidin 3-rhamnoside 5-glucoside C28H33O15+
Relative Intensity(%)
M/Z ratio
91.0579
239.0946
299.0607
355.0686

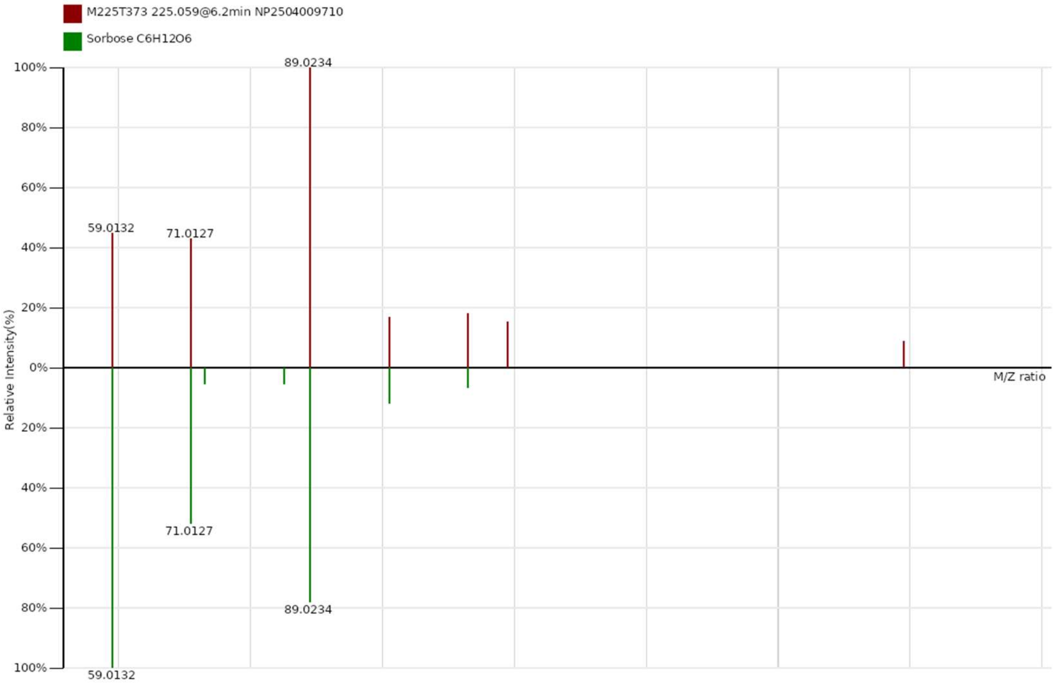
M225T373 225.059@6.2min NP2504009710
Sorbose C6H12O6
Relative Intensity(%)
M/Z ratio
59.0132
71.0127
89.0234

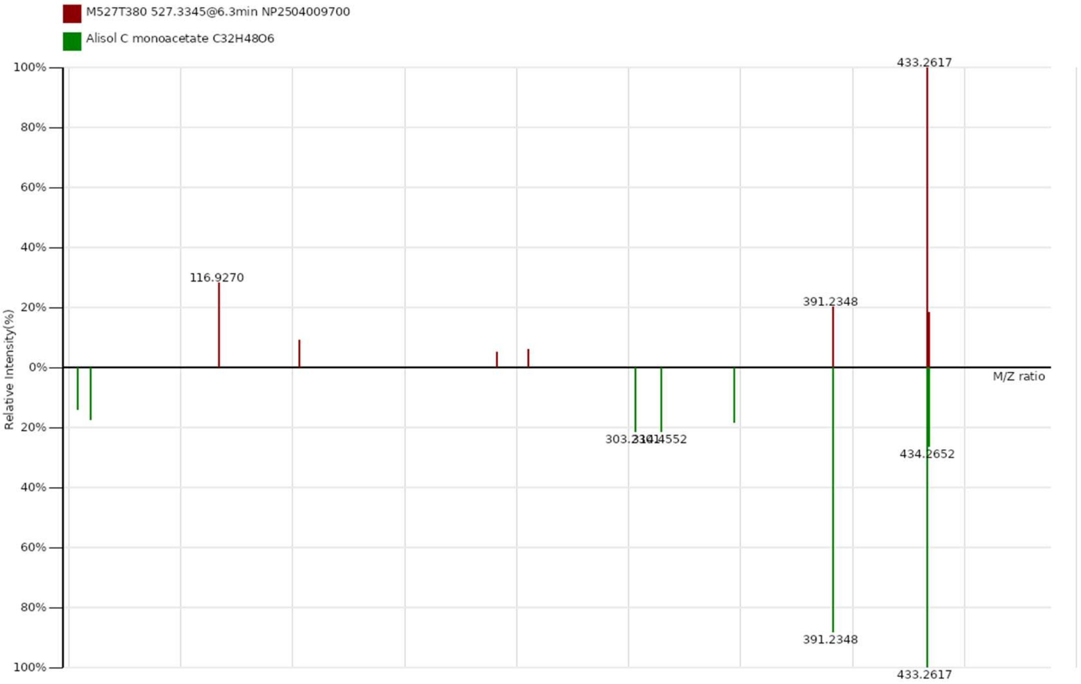

M527T380 527.3345@6.3min NP2504009700
Alisol C monoacetate C32H48O6
100%
80%
60%
40%
20%
0%
20%
40%
60%
80%
100%
Relative Intensity(%)
M/Z ratio
433.2617
116.9270
391.2348
434.2652
391.2348
433.2617

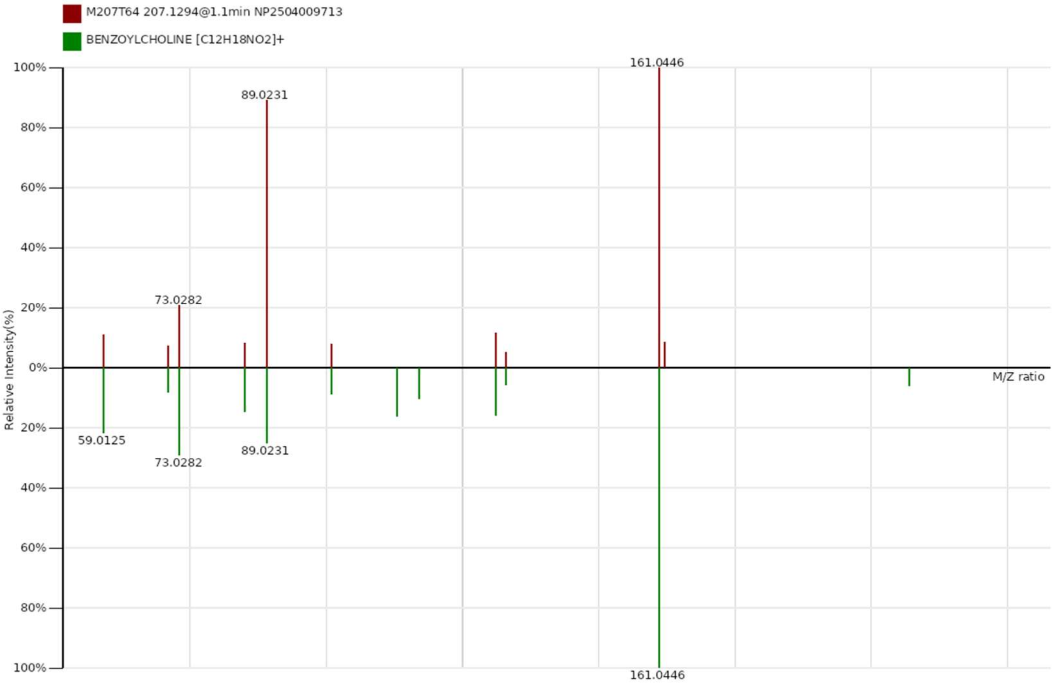

M207T64 207.1294@1.1min NP2504009713
BENZOYLCHOLINE [C12H18NO2]+
100%
80%
60%
40%
20%
0%
20%
40%
60%
80%
100%
Relative Intensity(%)
M/Z ratio
161.0446
89.0231
73.0282
59.0125
73.0282
89.0231
161.0446

Experimental fragments of the top 30 significant differential metabolites (DMs) from the comparison of B96H vs. BS72H.

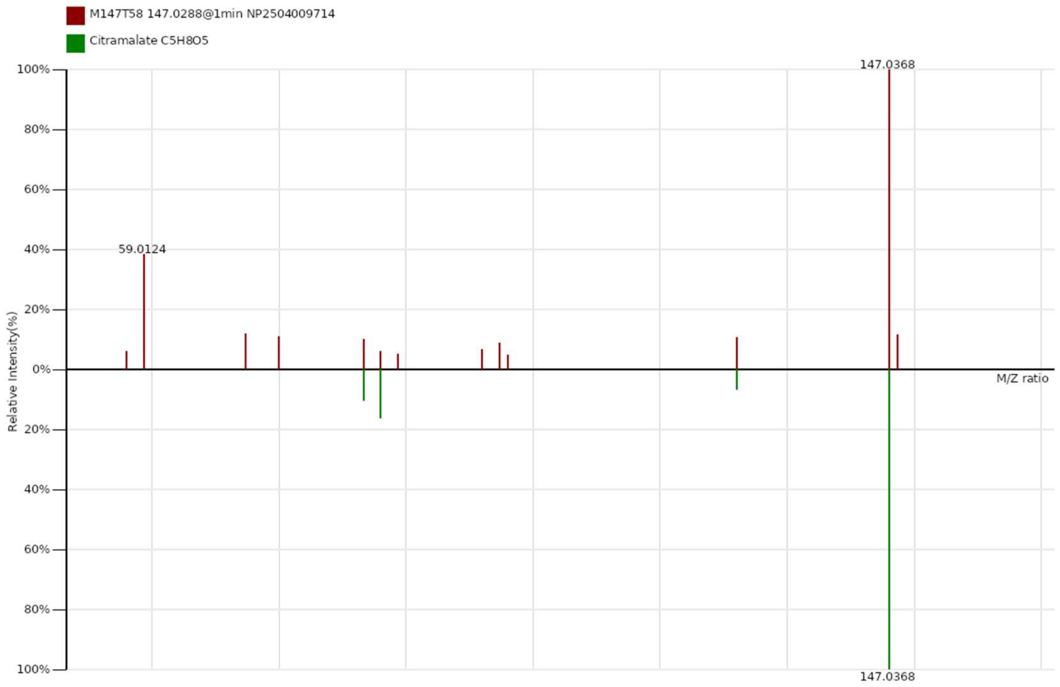

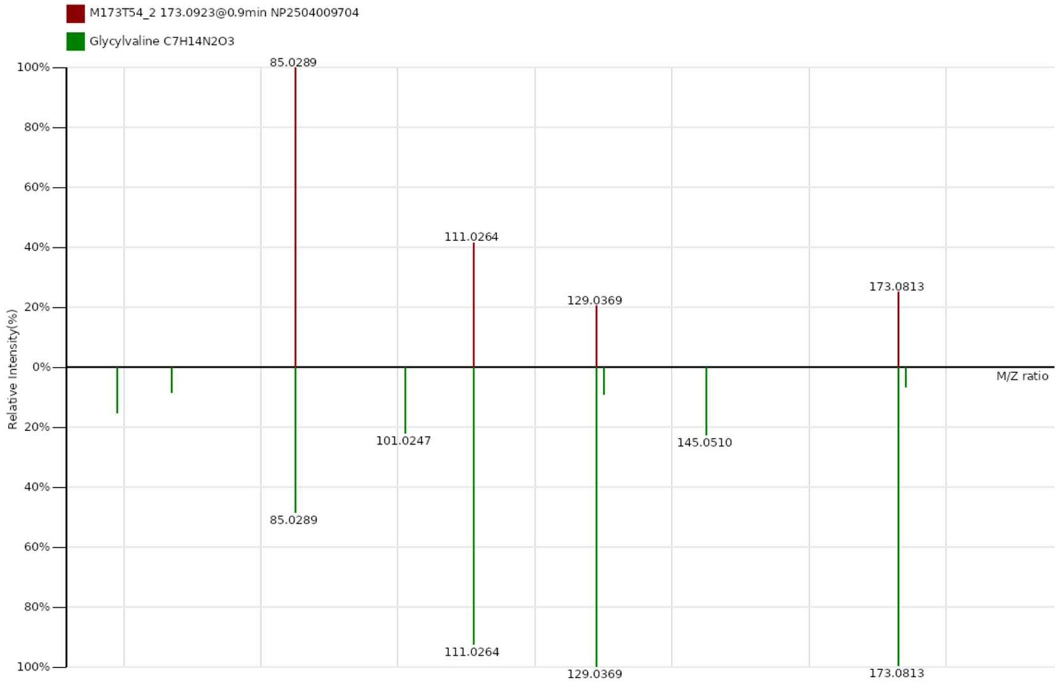

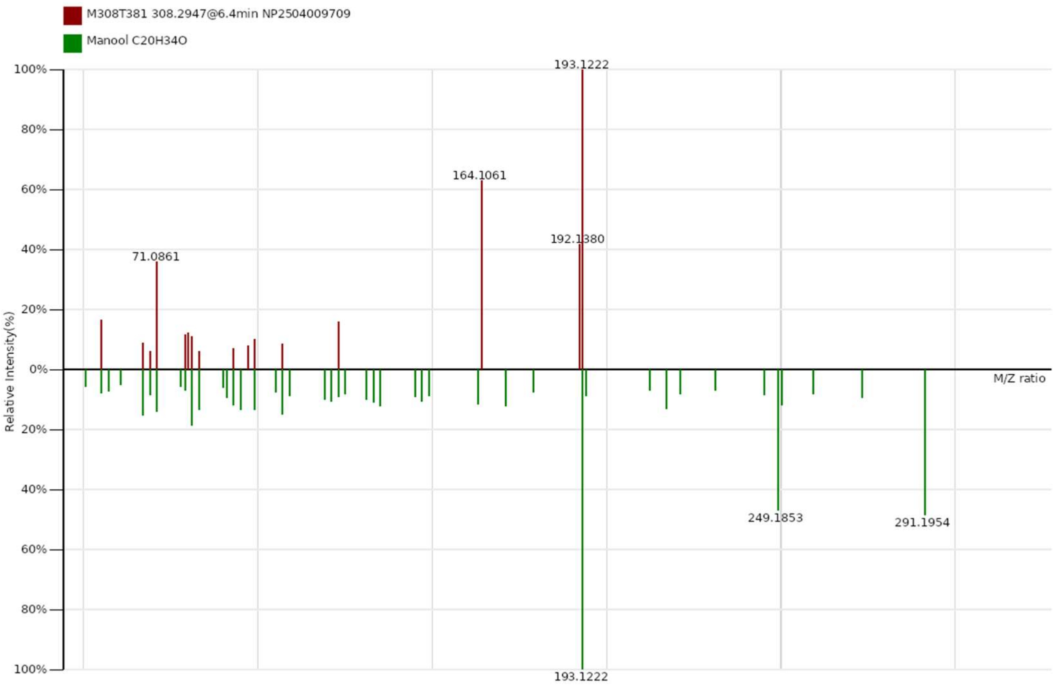
M308T381 308.2947@6.4min NP2504009709
Manool C20H34O
100%
80%
60%
40%
20%
0%
20%
40%
60%
80%
100%
Relative Intensity(%)
M/Z ratio
193.1222
164.1061
192.1380
71.0861
249.1853
291.1954
193.1222

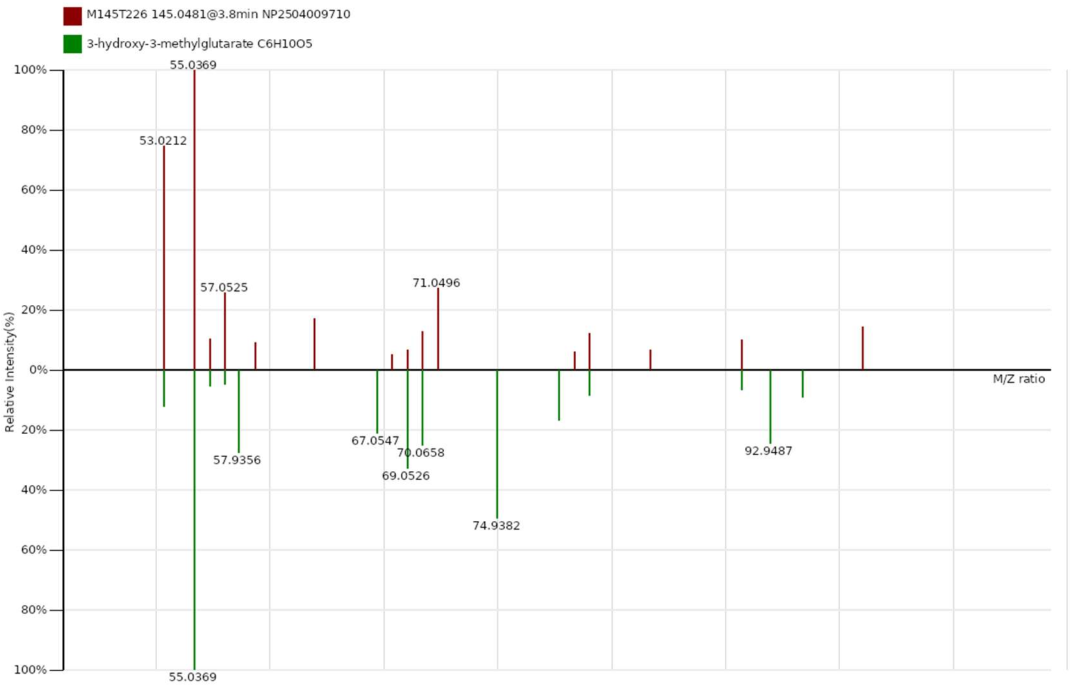
M145T226 145.0481@3.8min NP2504009710
3-hydroxy-3-methylglutarate C6H10O5
100%
80%
60%
40%
20%
0%
20%
40%
60%
80%
100%
Relative Intensity(%)
M/Z ratio
55.0369
53.0212
57.0525
71.0496
57.9356
67.0547
70.0658
69.0526
74.9382
92.9487
55.0369

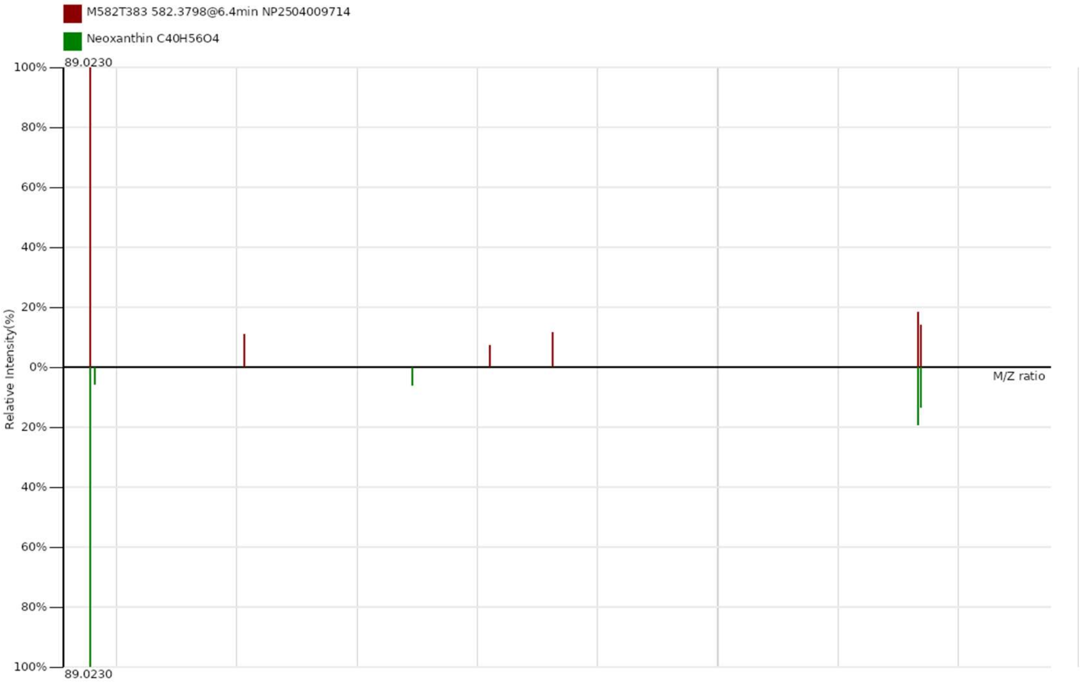

M582T383 582.3798@6.4min NP2504009714
Neoxanthin C40H56O4
89.0230
100%
80%
60%
40%
20%
0%
Relative Intensity(%)
M/Z ratio
89.0230

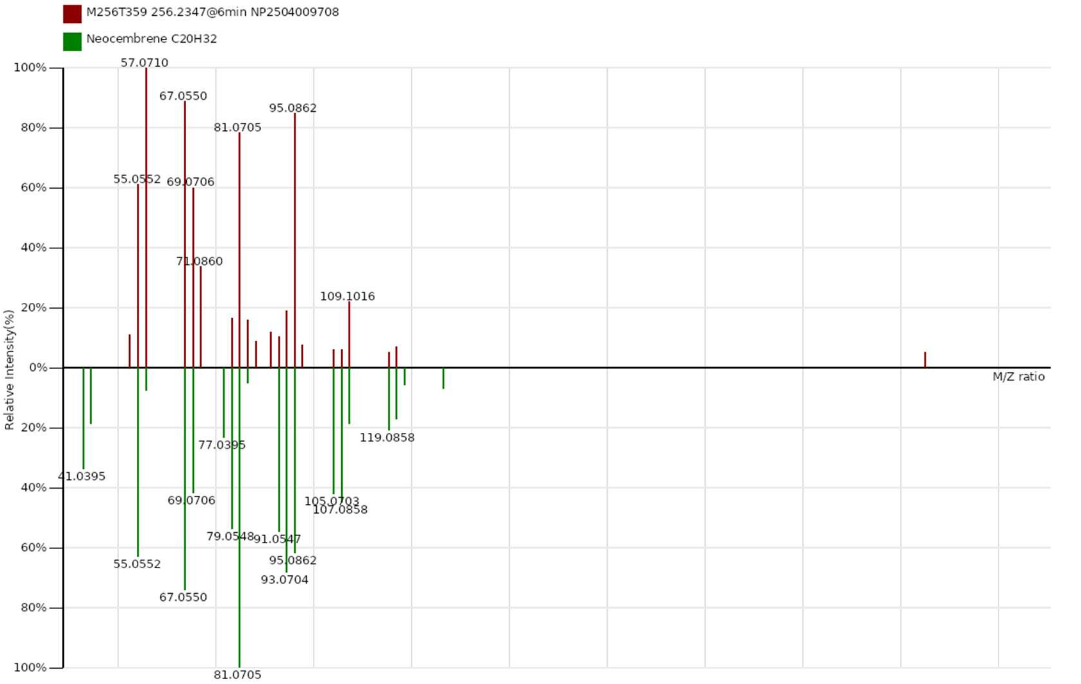

M256T359 256.2347@6min NP2504009708
Neocembrene C20H32
57.0710
67.0550
95.0862
81.0705
55.0552
69.0706
71.0860
109.1016
Relative Intensity(%)
M/Z ratio
77.0395
119.0858
41.0395
69.0706
105.0703
107.0858
79.0548
91.0547
55.0552
95.0862
93.0704
67.0550
81.0705

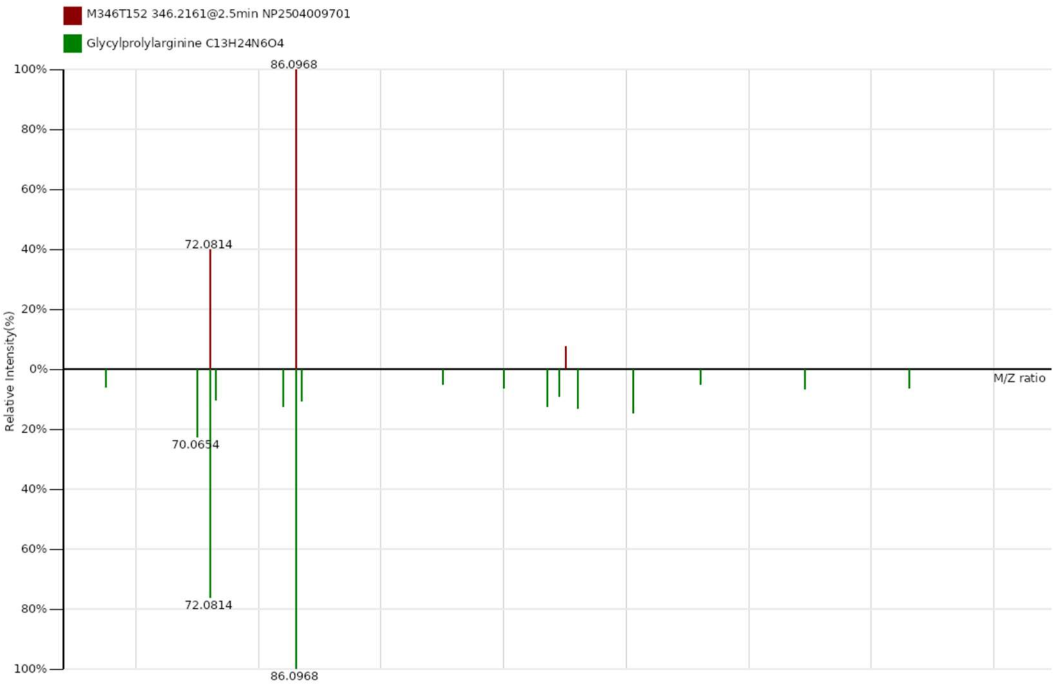

M346T152 346.2161@2.5min NP2504009701
Glycylprolylarginine C13H24N6O4
Relative Intensity(%)
M/Z ratio
86.0968
72.0814
70.0654
72.0814
86.0968

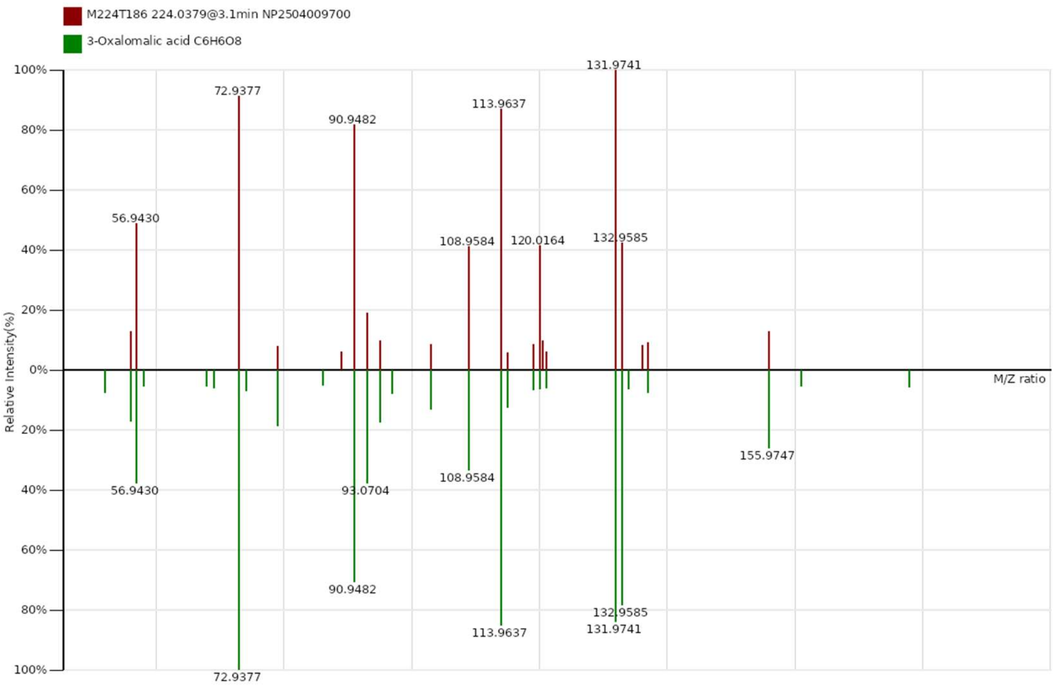

M224T186 224.0379@3.1min NP2504009700
3-Oxalomalic acid C6H6O8
Relative Intensity(%)
M/Z ratio
131.9741
72.9377
90.9482
113.9637
56.9430
108.9584
120.0164
132.9585
56.9430
93.0704
108.9584
155.9747
90.9482
113.9637
132.9585
131.9741
72.9377

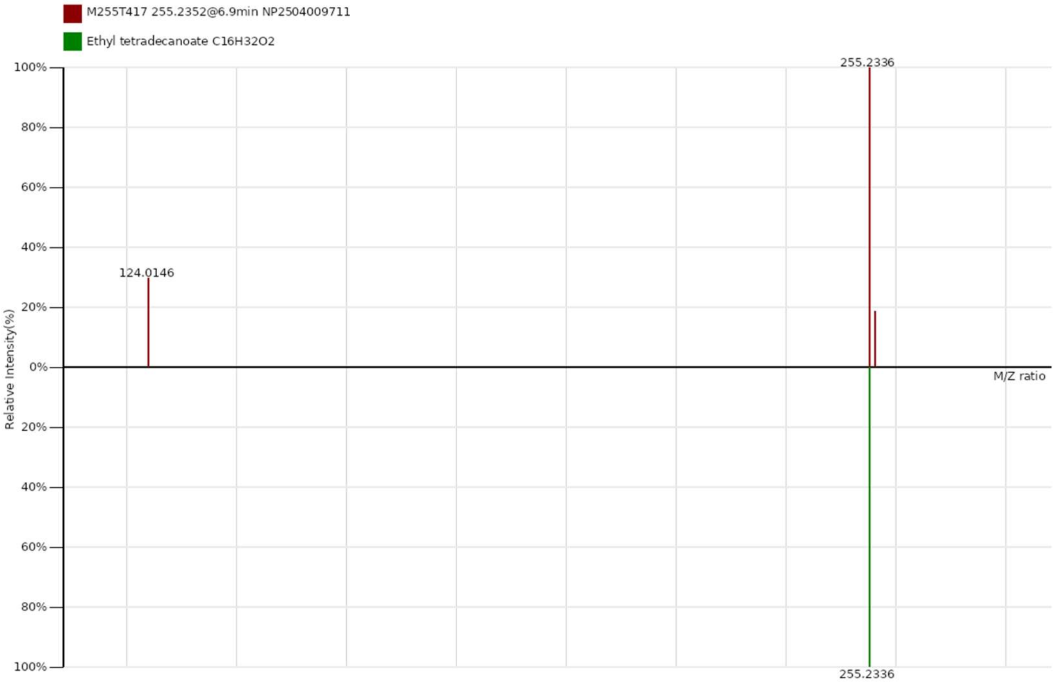
M255T417 255.2352@6.9min NP2504009711
Ethyl tetradecanoate C16H32O2
100%
80%
60%
40%
20%
0%
20%
40%
60%
80%
100%
Relative Intensity(%)
M/Z ratio
124.0146
255.2336
255.2336

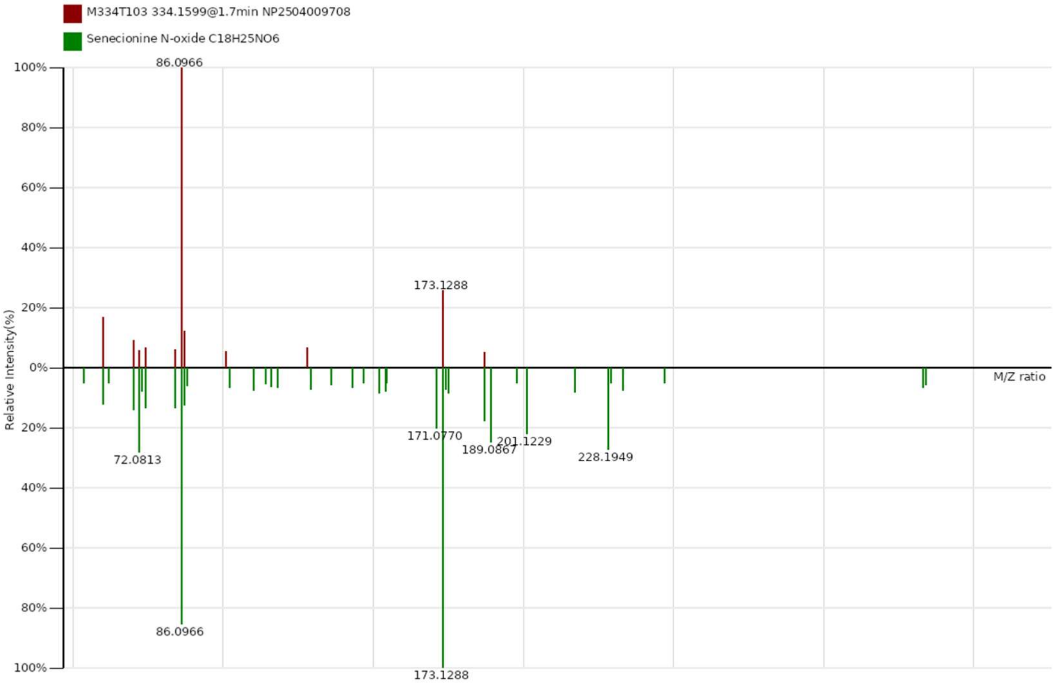
M334T103 334.1599@1.7min NP2504009708
Senecionine N-oxide C18H25NO6
100%
80%
60%
40%
20%
0%
20%
40%
60%
80%
100%
Relative Intensity(%)
M/Z ratio
86.0966
173.1288
72.0813
171.0770
189.0867
201.1229
228.1949
86.0966
173.1288

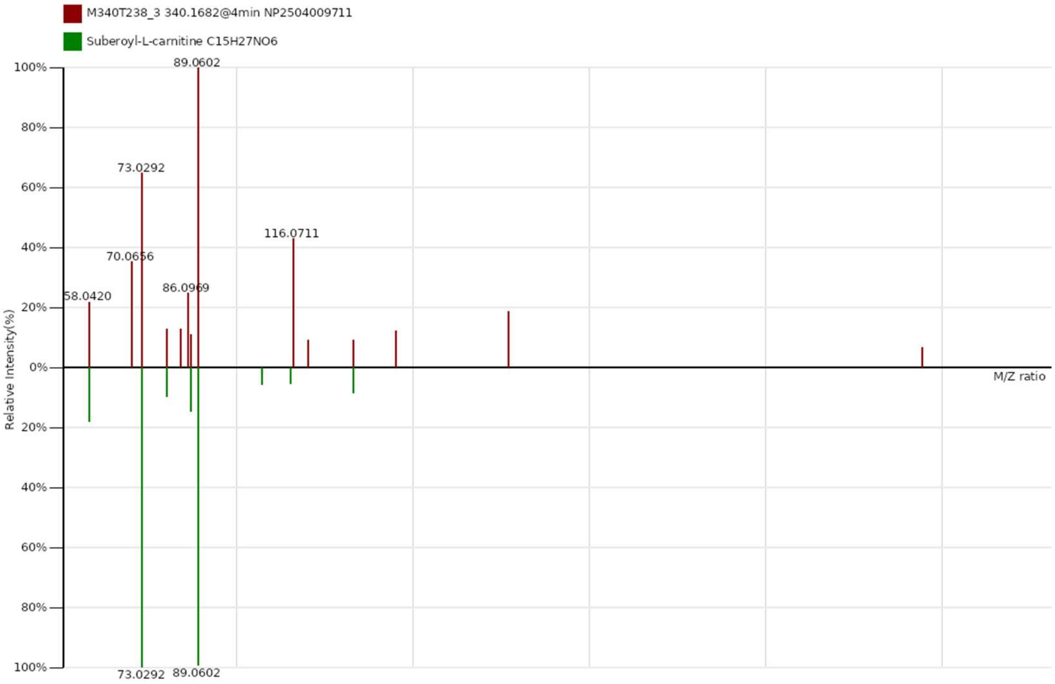
M340T238_3 340.1682@4min NP2504009711
Suberoyl-L-carnitine C15H27NO6
100%
80%
60%
40%
20%
0%
20%
40%
60%
80%
100%
Relative Intensity(%)
M/Z ratio
89.0602
73.0292
116.0711
70.0656
86.0969
58.0420
73.0292
89.0602

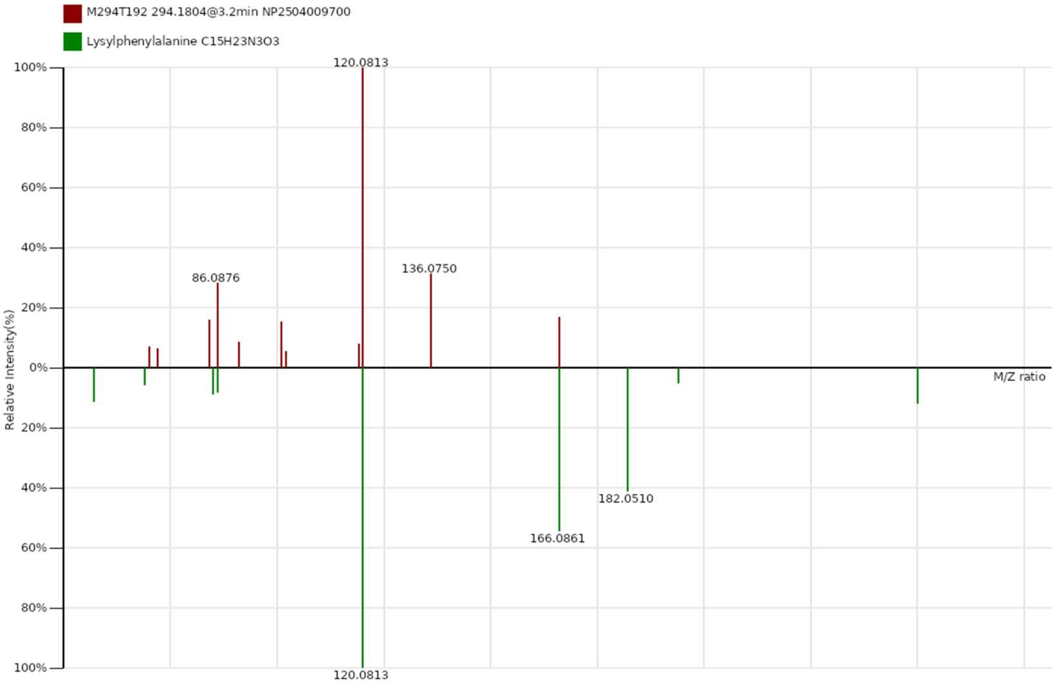
M294T192 294.1804@3.2min NP2504009700
Lysylphenylalanine C15H23N3O3
100%
80%
60%
40%
20%
0%
20%
40%
60%
80%
100%
Relative Intensity(%)
M/Z ratio
120.0813
136.0750
86.0876
182.0510
166.0861
120.0813

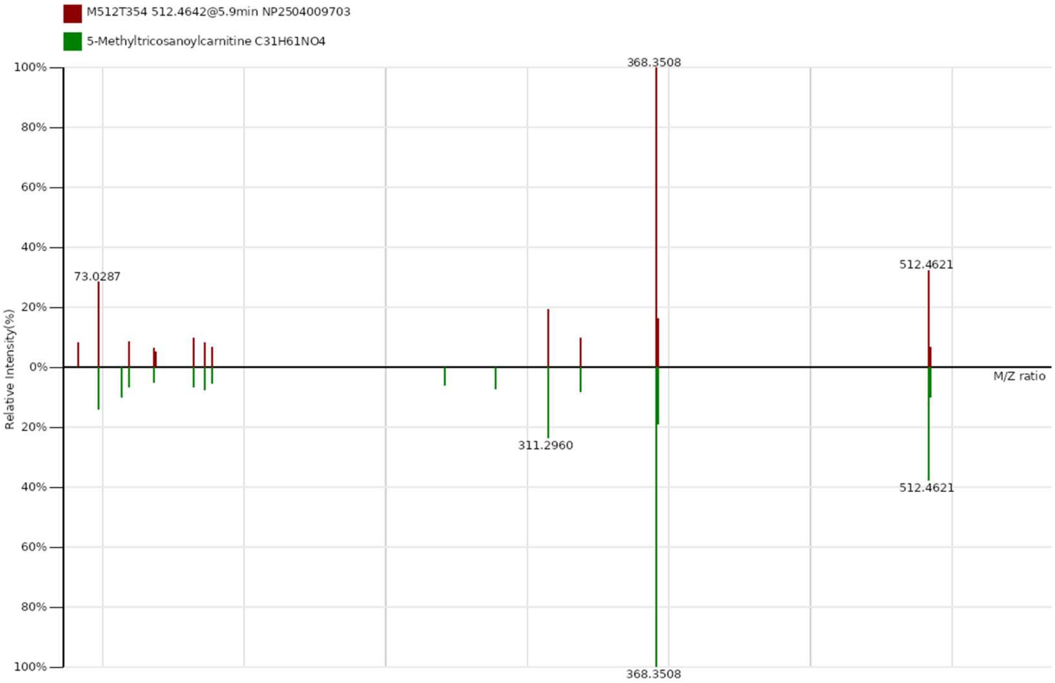

M512T354 512.4642@5.9min NP2504009703
5-Methyltricosanoylcarnitine C31H61NO4
100%
80%
60%
40%
20%
0%
20%
40%
60%
80%
100%
Relative Intensity(%)
M/Z ratio
368.3508
73.0287
512.4621
311.2960
512.4621
368.3508

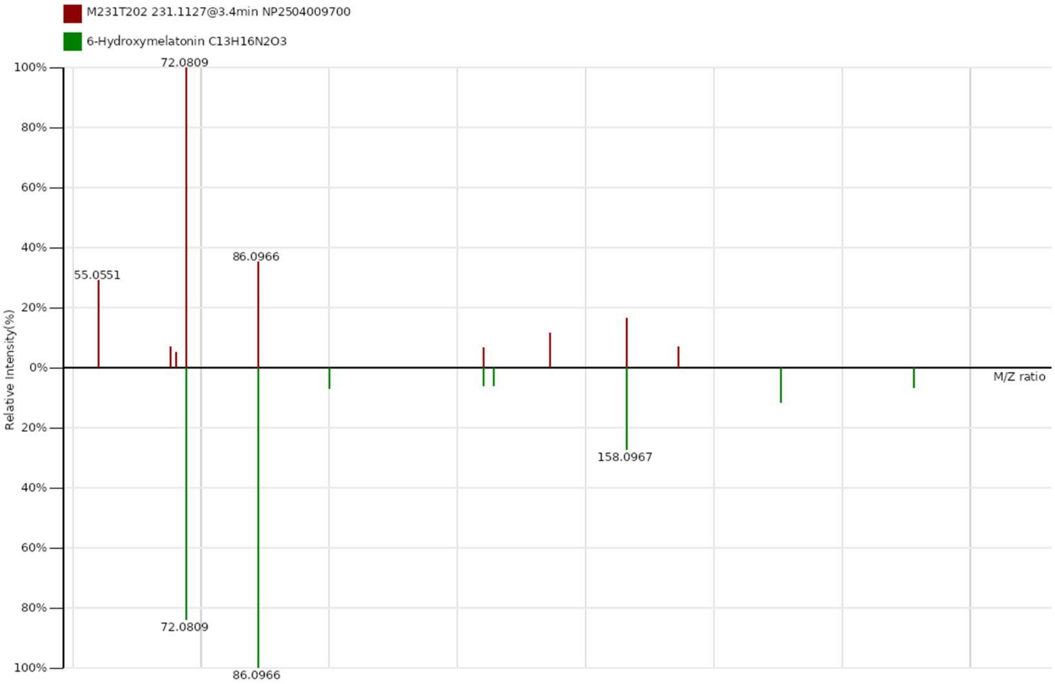

M231T202 231.1127@3.4min NP2504009700
6-Hydroxymelatonin C13H16N2O3
100%
80%
60%
40%
20%
0%
20%
40%
60%
80%
100%
Relative Intensity(%)
M/Z ratio
72.0809
86.0966
55.0551
158.0967
72.0809
86.0966

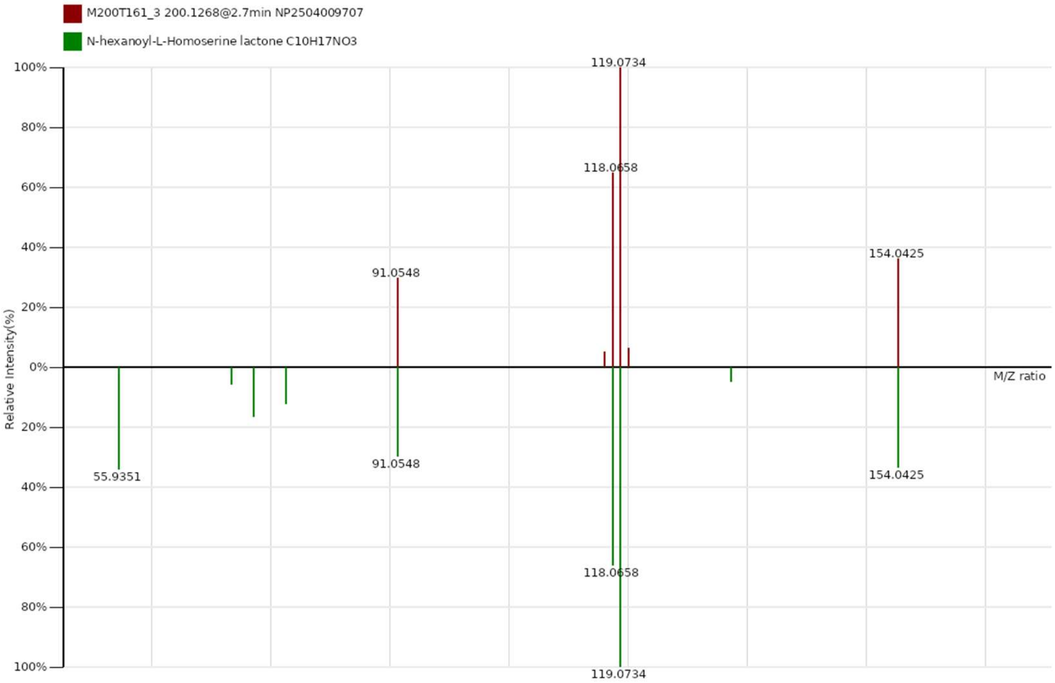

M200T161_3 200.1268@2.7min NP2504009707
N-hexanoyl-L-Homoserine lactone C10H17NO3
100%
80%
60%
40%
20%
0%
20%
40%
60%
80%
100%
Relative Intensity(%)
M/Z ratio
119.0734
118.0658
91.0548
154.0425
55.9351
91.0548
154.0425
118.0658
119.0734

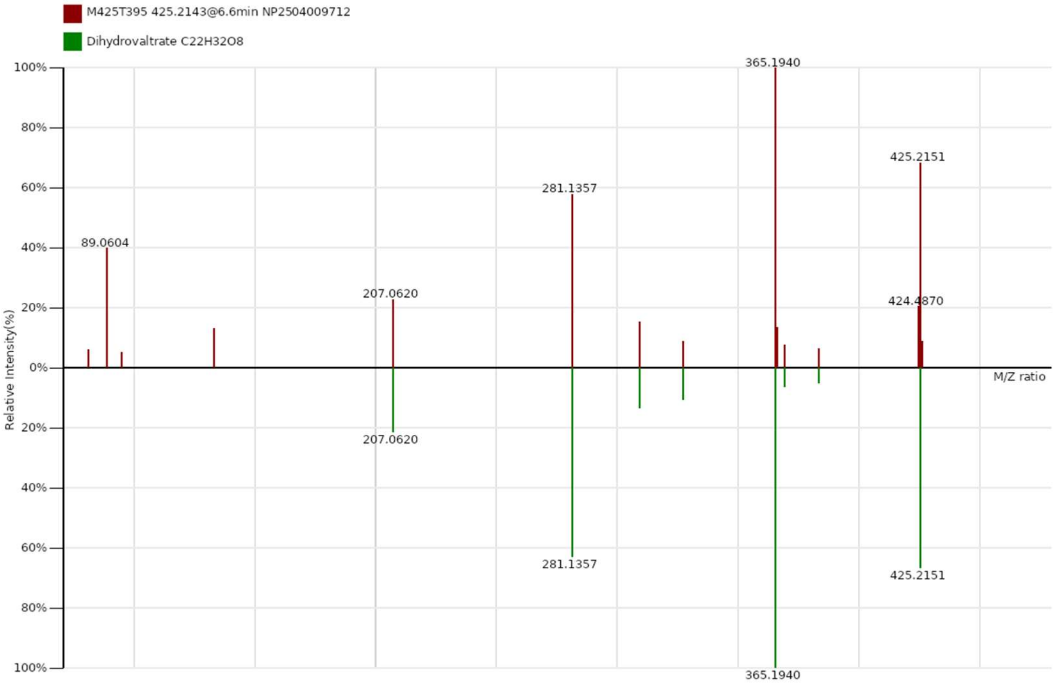

M425T395 425.2143@6.6min NP2504009712
Dihydrovaltrate C22H32O8
100%
80%
60%
40%
20%
0%
20%
40%
60%
80%
100%
Relative Intensity(%)
M/Z ratio
365.1940
425.2151
281.1357
89.0604
207.0620
424.4870
207.0620
281.1357
425.2151
365.1940

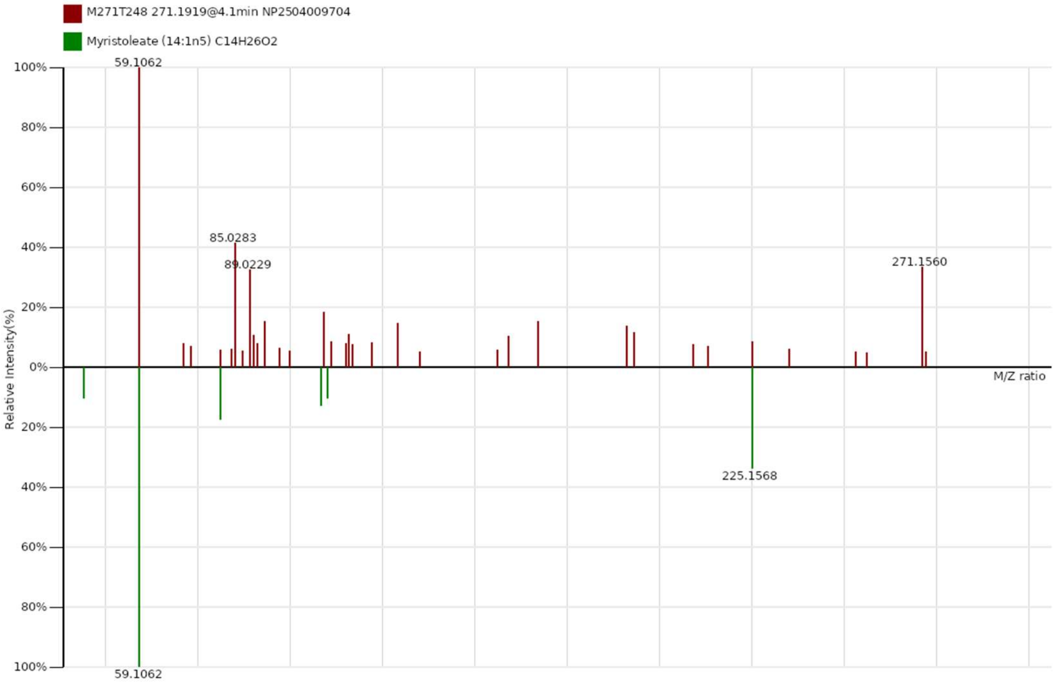
M271T248 271.1919@4.1min NP2504009704
Myristoleate (14:1n5) C14H26O2
Relative Intensity(%)
M/Z ratio
59.1062
85.0283
89.0229
271.1560
225.1568
59.1062

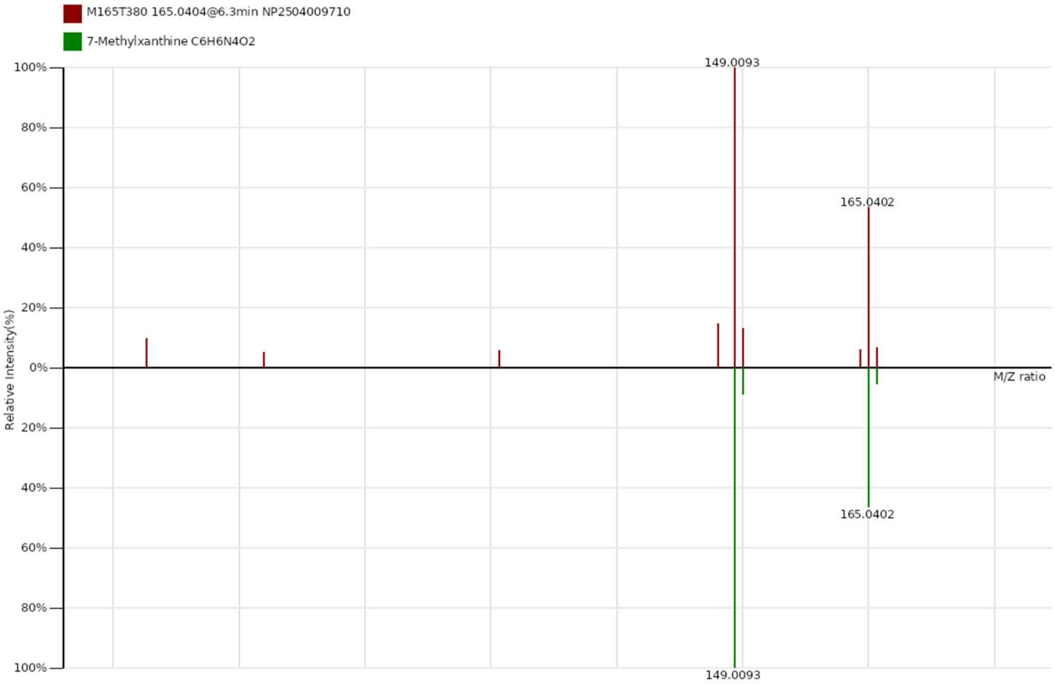
M165T380 165.0404@6.3min NP2504009710
7-Methylxanthine C6H6N4O2
Relative Intensity(%)
M/Z ratio
149.0093
165.0402
165.0402
149.0093

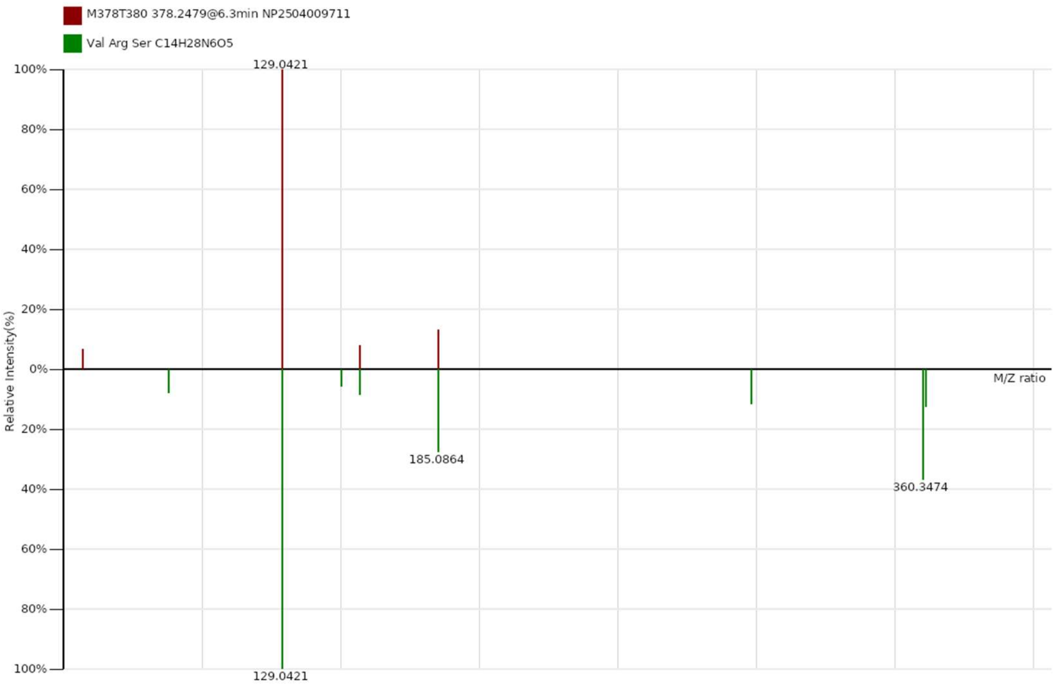
M378T380 378.2479@6.3min NP2504009711
Val Arg Ser C14H28N6O5
100%
80%
60%
40%
20%
0%
Relative Intensity(%)
M/Z ratio
129.0421
185.0864
360.3474
129.0421

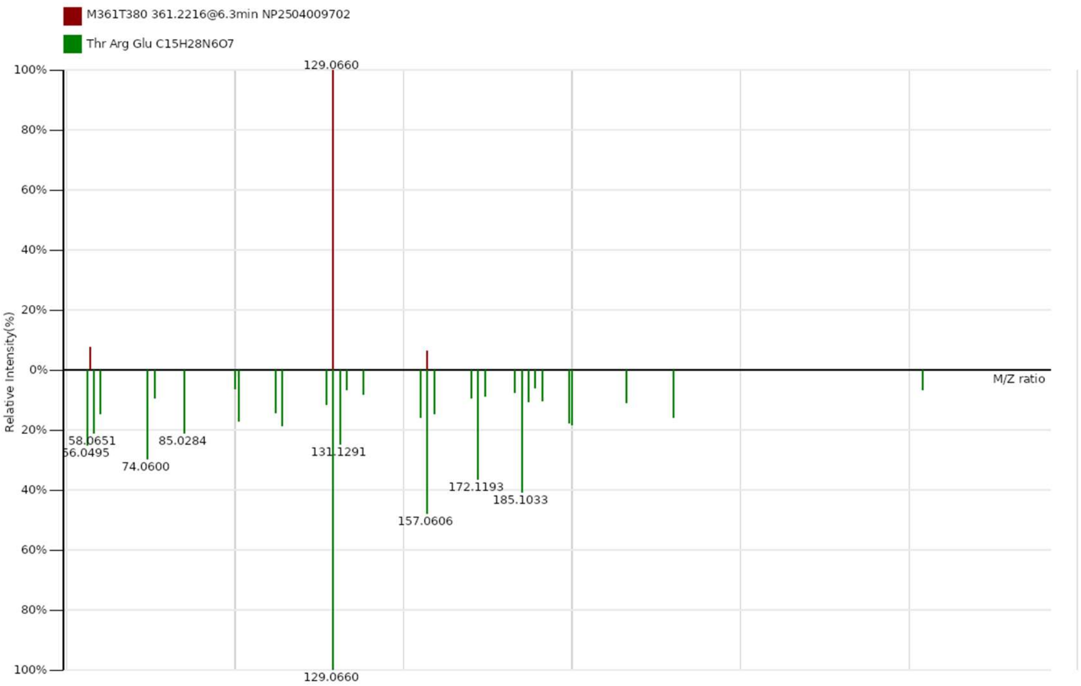
M361T380 361.2216@6.3min NP2504009702
Thr Arg Glu C15H28N6O7
100%
80%
60%
40%
20%
0%
Relative Intensity(%)
M/Z ratio
129.0660
58.0651
56.0495
74.0600
85.0284
131.1291
157.0606
172.1193
185.1033
129.0660

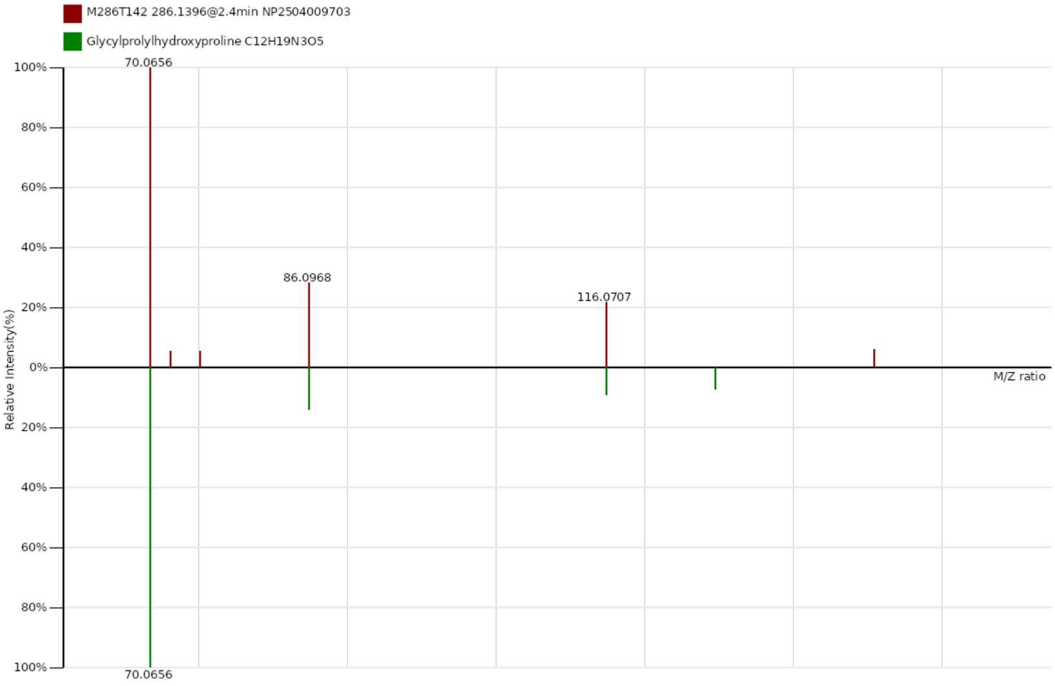
M286T142 286.1396@2.4min NP2504009703
Glycylprolylhydroxyproline C12H19N3O5
70.0656
86.0968
116.0707
70.0656
Relative Intensity(%)
M/Z ratio
100%
80%
60%
40%
20%
0%
20%
40%
60%
80%
100%

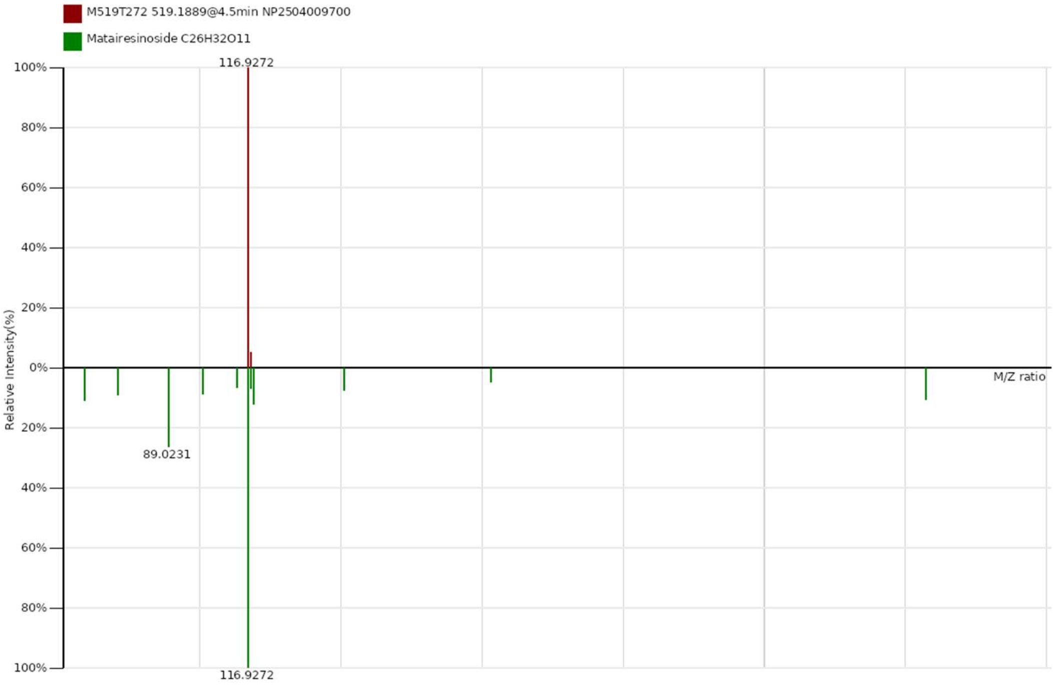
M519T272 519.1889@4.5min NP2504009700
Matairesinoside C26H32O11
116.9272
89.0231
116.9272
Relative Intensity(%)
M/Z ratio
100%
80%
60%
40%
20%
0%
20%
40%
60%
80%
100%

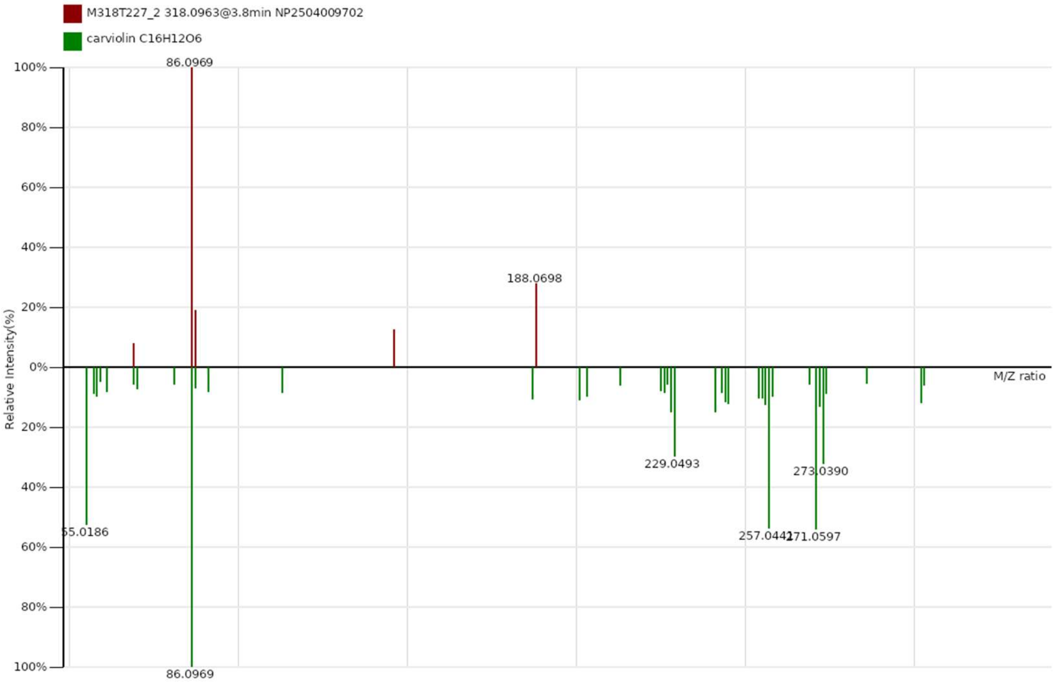

M318T227_2 318.0963@3.8min NP2504009702
carviolin C16H12O6
Relative Intensity(%)
M/Z ratio
86.0969
188.0698
55.0186
229.0493
273.0390
257.0441
271.0597
86.0969

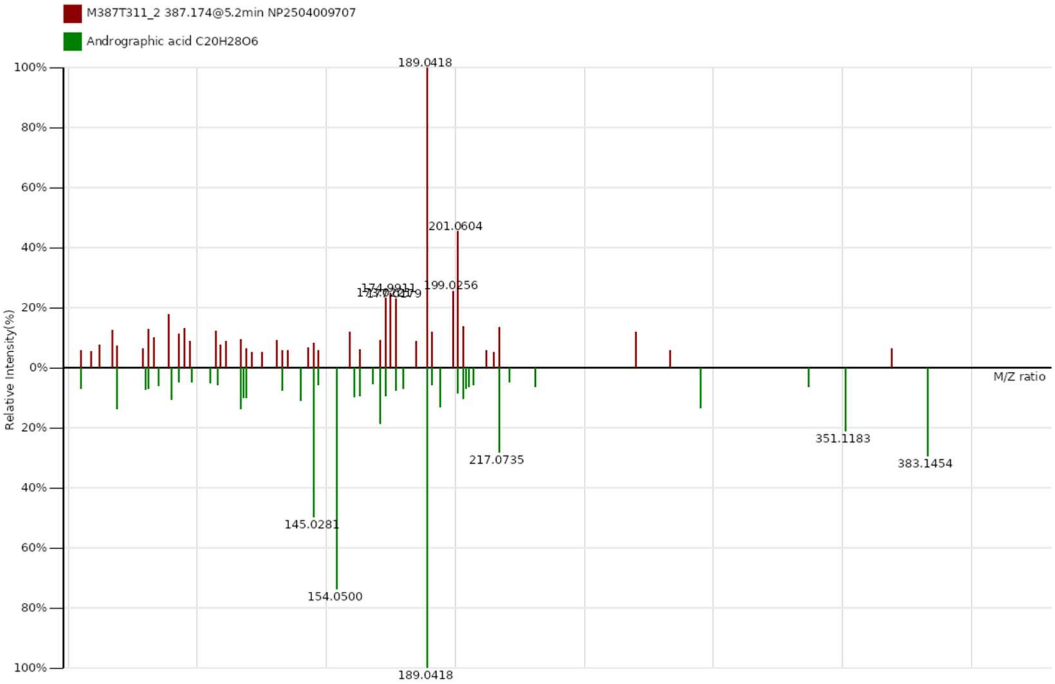

M387T311_2 387.174@5.2min NP2504009707
Andrographic acid C20H28O6
Relative Intensity(%)
M/Z ratio
189.0418
201.0604
174.0911
199.0256
217.0735
351.1183
383.1454
145.0281
154.0500
189.0418

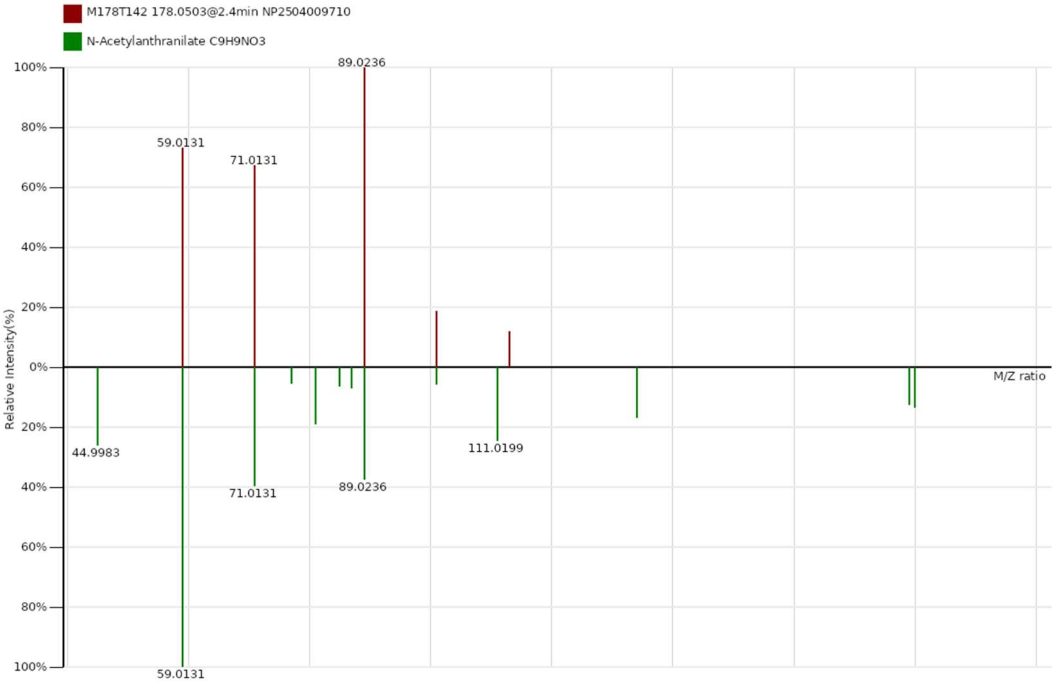
M178T142 178.0503@2.4min NP2504009710
N-Acetylanthranilate C9H9NO3
Relative Intensity(%)
M/Z ratio
100%
80%
60%
40%
20%
0%
89.0236
59.0131
71.0131
44.9983
111.0199
71.0131
89.0236
59.0131

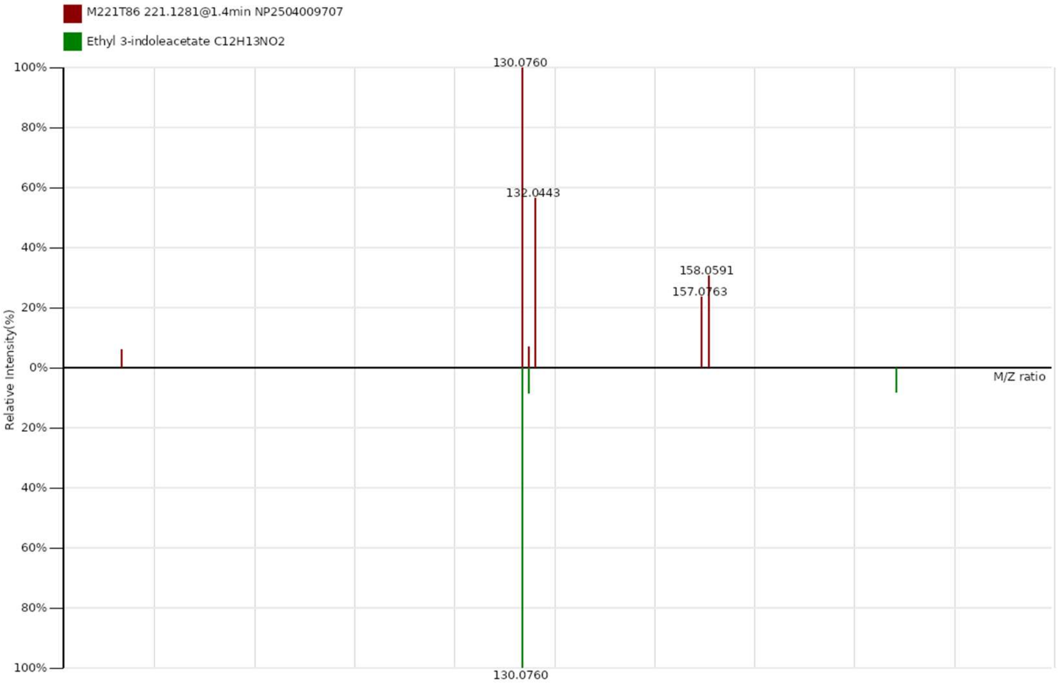
M221T86 221.1281@1.4min NP2504009707
Ethyl 3-indoleacetate C12H13NO2
Relative Intensity(%)
M/Z ratio
100%
80%
60%
40%
20%
0%
130.0760
132.0443
158.0591
157.0763
130.0760

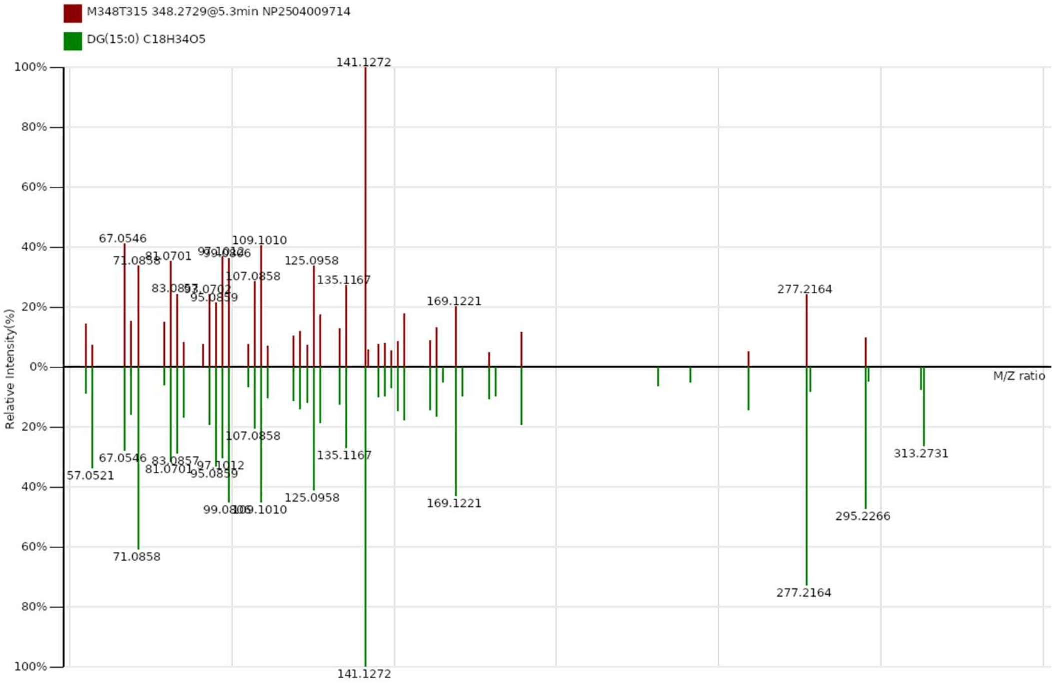
M348T315 348.2729@5.3min NP2504009714
DG(15:0) C18H34O5
Relative Intensity(%)
M/Z ratio
141.1272
67.0546
71.0858
81.0701
83.0857
97.1012
99.0806
95.0859
109.1010
107.0858
125.0958
135.1167
169.1221
277.2164
57.0521
295.2266
313.2731

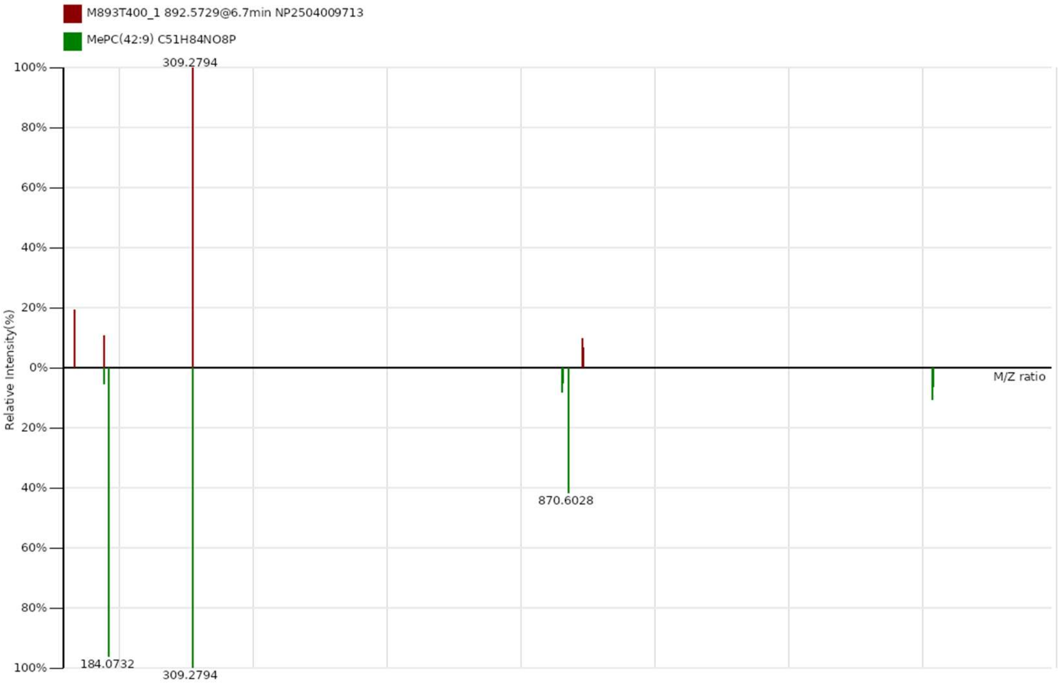
M893T400_1 892.5729@6.7min NP2504009713
MePC(42:9) C51H84NO8P
Relative Intensity(%)
M/Z ratio
309.2794
870.6028
184.0732

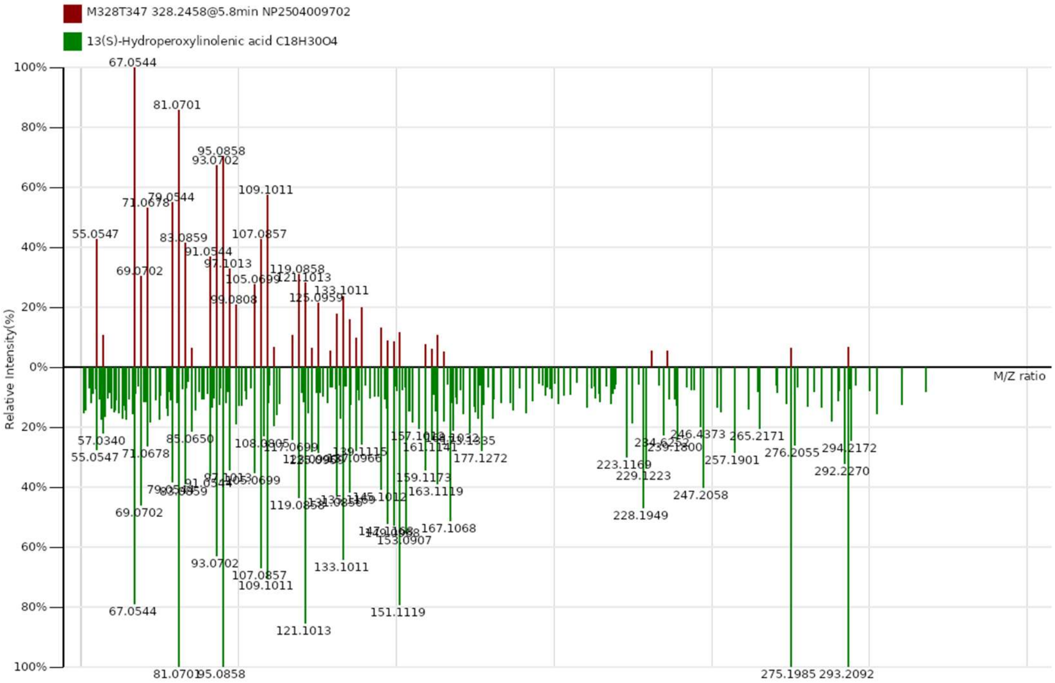
M328T347 328.2458@5.8min NP2504009702
13(S)-Hydroperoxylinolenic acid C18H30O4
Relative Intensity(%)
M/Z ratio
67.0544
81.0701
95.0858
93.0702
109.1011
107.0857
55.0547
69.0702
275.1985
293.2092
151.1119
121.1013
133.1011

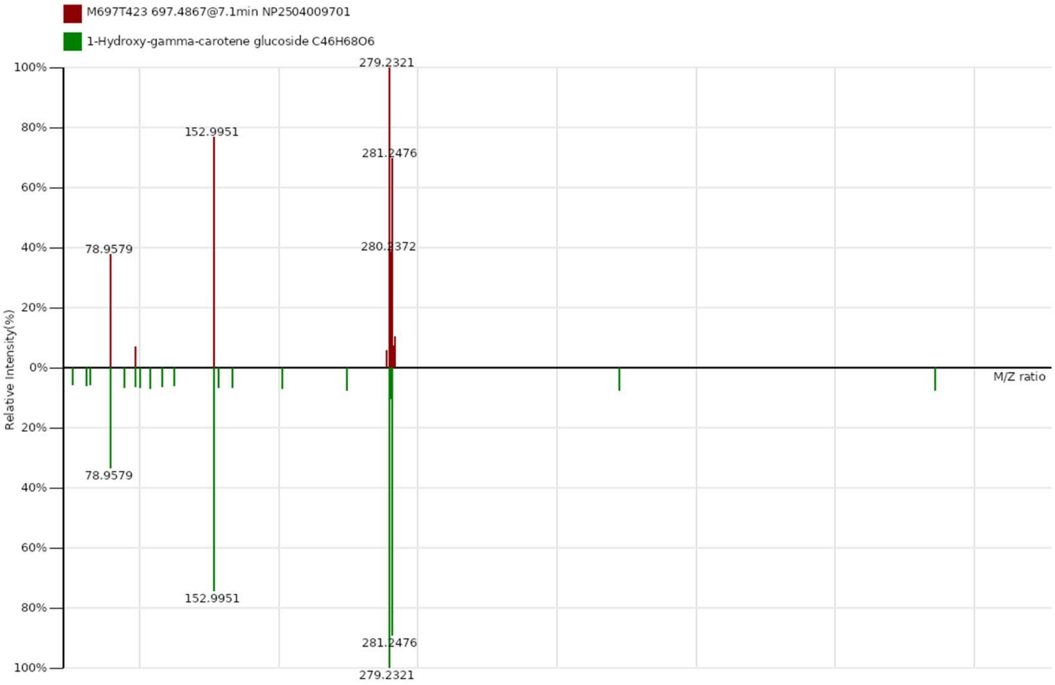
M697T423 697.4867@7.1min NP2504009701
1-Hydroxy-gamma-carotene glucoside C46H68O6
Relative Intensity(%)
M/Z ratio
279.2321
152.9951
281.2476
280.2372
78.9579
78.9579
152.9951
281.2476
279.2321
